# Supplementary material for: Disulfide-constrained peptide scaffolds enable a robust peptide-therapeutic discovery platform
Source: PLoS One. 2024 Mar 28;19(3):e0300135. doi: 10.1371/journal.pone.0300135 (PMC10977697; doi:10.1371/journal.pone.0300135)
Supplement: S1 File — A zip file contains 51 pdf files with filenames are the same as the “DCP name” listed in the tables. (ZIP) [file pone.0300135.s004.zip › Her3-Fc_CBD1.pdf]

Quality Control Record

Product: Her3-Fc_CBD1
Sequence: Gly-Pro-Thr-Gln-Ser-Trp-Gln-Gly-Arg-Cys-Gly-Gly-Ile-Gly-Tyr-Ser-Gly-Pro-Thr-Val-Cys-Ala-Ser-Gly-Thr-Thr-Cys-Gln-Val-Leu-Thr-Pro-Tyr-Trp-Ser-Glu-Cys-Leu-Pro-Gly-Ala-Ser

Note: Natural Oxidation

Product No.: GT1313 Expected M.W.: 4260.68 Found M.W.: 4260.90 Lot: X100

APPEARANCE:	White Powder
MOLECULAR WEIGHT VERIFICATION:	Confirmed
PURITY: Instrument: Agilent 1290<br>Condition: HPLC column in TFA System<br>Gradient: 25-55% Buffer B in 20 minutes<br>Buffer A: 0.1% TFA in H2O<br>Buffer B: 0.1% TFA in ACN<br>Wavelength: 214 nm<br>Column: Phenomenex Luna C18 5µm 100Å, 4.6 x 250 mm	96.76%
PEPTIDE CONTENT:<br>(By N Elemental Analysis)	82.9%
ELLMAN'S TEST:	Complies
SUGGESTIONS FOR PEPTIDE DISSOLUTION:	Acetonitrile / 0.1% TFA in Water
COUNTERIONS PRESENT:	TFA Salt
STORAGE:	All peptides should be stored dry at -20°C

This material is NOT FOR HUMAN USE. This material is not listed as hazardous by ∗NIOSH/RTECS. Therefore, no SAFETY DATA SHEET is required. However, the chemical, physical and toxicological properties of this product have not been thoroughly investigated. Therefore, please exercise due care when handling this material. This action is in compliance with State and Federal OSHA standards and regulations.

Quality Control: ______

Date: February 4, 2022

CSBio
20 Kelly Court, Menlo Park, CA 94025 USA
T: (650) 322 1111 • F: (650) 322 2278
www.csbio.com • peptides@csbio.com

Compound: GT1313 Her3-Fc_CBD1 Lot Number: X100 Expected M.W.: 4260.68 Found M.W.: 4260.90

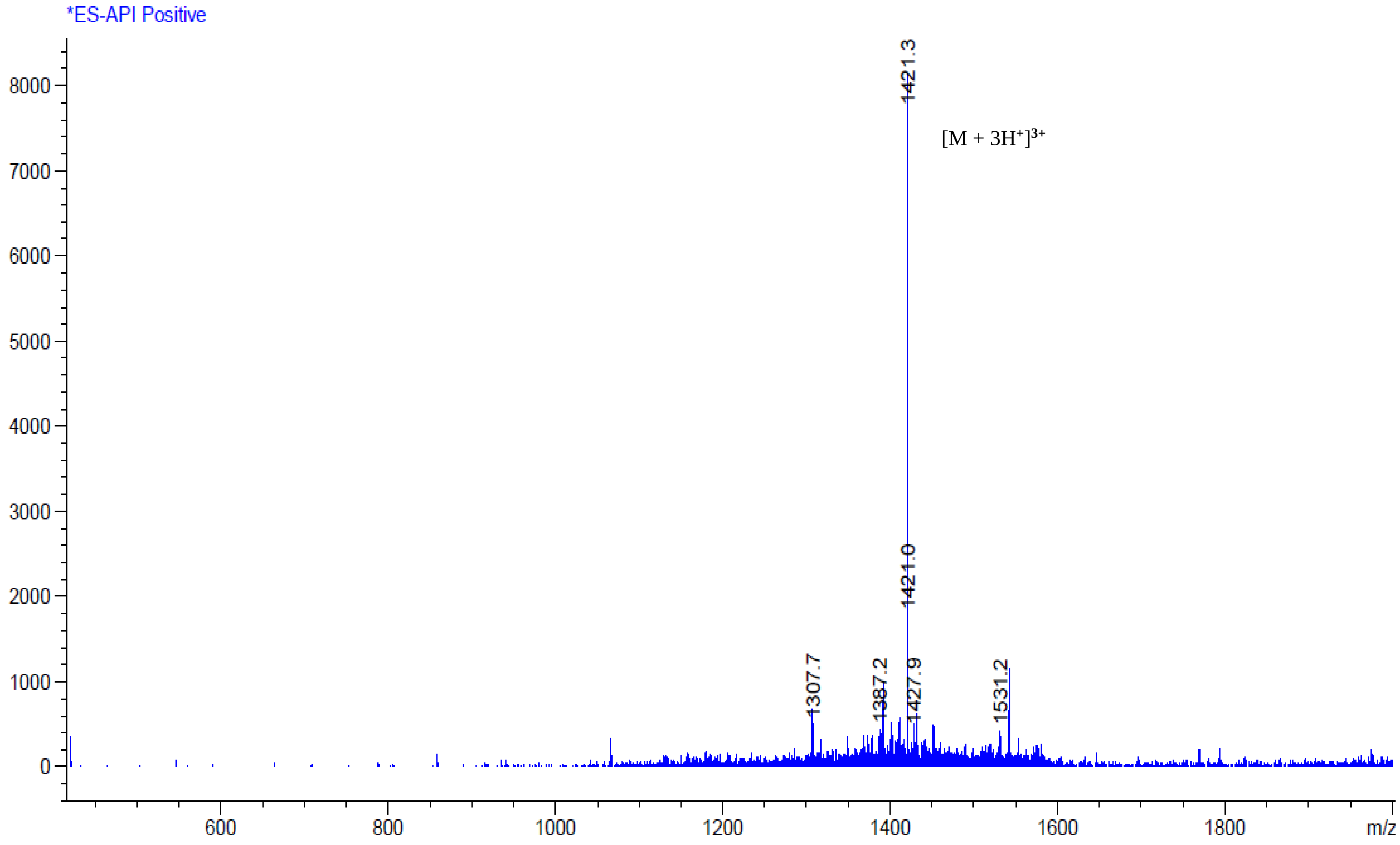

Sample Name: GT1313
Lot# X100
Instrument 1 Agilent 1290
Instrument ID: E195
Injection Date: 1/28/2022
Inj. Volume: 20.0 uL

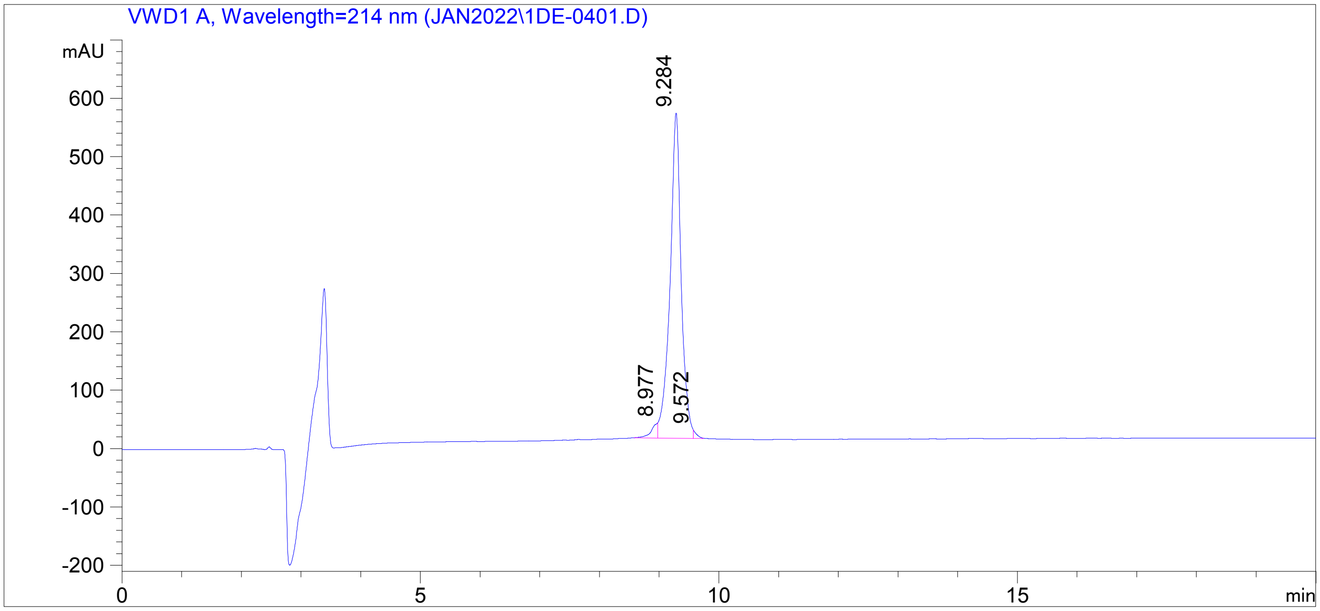

Data file name: C:\CHEM32\1\DATA\JAN2022\1DE-0401.D
Acq. Method: C:\Chem32\1\DATA\JAN2022\QC 2022-01-28 09-18-14\25-55-20-1-2.M

Column: Phenomenex Luna C18 (2) 5u 100A 250x4.6mm P/N: 00G-4252-E0
Buffer A: 0.1% TFA in H2O
Buffer B: 0.1% TFA in ACN
Wavelength: 214 nm
Flow Rate: 1 ml/minute
Column Temperature: 25C
Gradient: 25%-55% B in 20 minutes

Peak #	RT [min]	Area	Height	Area %
1	8.977	191.43	26.05	2.56
2	9.284	7242.51	557.05	96.76
3	9.572	51.43	14.43	0.69

Peptide Content with Elemental Analysis

Analysis: Determination of Peptide Content by Nitrogen Content
Instrument Model: Perkin Elmer Series II CHNS/O Analyser 2400
Sample Name: Her3-Fc_CBD1
Sample ID: GT1313
Lot Number: X100
Sample Testing Date: 2/4/2022

	N%
**Expected Content**	16.44
**Actual Content**	13.62
**Peptide Content (%)**	**82.9**

Performed by:

2/4/2022

Name　　Date

Reviewed by:

2/4/2022

Name　　Date

CSBio
20 Kelly Court, Menlo Park, CA 94025 USA
Tel: 650-322-1111 Fax: 650-322-2278 Web: www.csbio.com Email: peptides@csbio.com
