# Supplementary material for: Disulfide-constrained peptide scaffolds enable a robust peptide-therapeutic discovery platform
Source: PLoS One. 2024 Mar 28;19(3):e0300135. doi: 10.1371/journal.pone.0300135 (PMC10977697; doi:10.1371/journal.pone.0300135)
Supplement: S1 File — A zip file contains 51 pdf files with filenames are the same as the “DCP name” listed in the tables. (ZIP) [file pone.0300135.s004.zip › Her3-Fc_ChTx1.pdf]

# Quality Control Record

Product: Her3-Fc_ChTx1
Sequence: Val-Ser-Cys-Thr-Thr-Ser-Lys-Glu-Cys-Trp-Ser-Val-Cys-Gln-Arg-Leu-His-Asn-Thr-Ser-Trp-Gly-Glu-Gln-Cys-Val-Thr-Lys-Phe-Cys-Lys-Cys-Glu-Glu

Note: Natural Oxidation

Product No.: GT1314 Expected M.W.: 3935.47 Found M.W.: 3934.20 Lot: X221

| | |
|---|---|
| APPEARANCE: | White Powder |
| MOLECULAR WEIGHT VERIFICATION: | Confirmed |
| PURITY: Instrument: Agilent 1290<br>Condition: HPLC column in TFA System<br>Gradient: 15-45% Buffer B in 20 minutes<br>Buffer A: 0.1% TFA in H2O<br>Buffer B: 0.1% TFA in ACN<br>Wavelength: 214 nm<br>Column: Phenomenex Luna C18 5µm 100Å, 4.6 x 250 mm | 90.31% |
| PEPTIDE CONTENT:<br>(By N Elemental Analysis) | Pending |
| ELLMAN'S TEST: | Complies |
| SUGGESTIONS FOR PEPTIDE DISSOLUTION: | 0.1% TFA in Water |
| COUNTERIONS PRESENT: | TFA Salt |
| STORAGE: | All peptides should be stored dry at -20°C |

This material is NOT FOR HUMAN USE. This material is not listed as hazardous by ∗NIOSH/RTECS. Therefore, no SAFETY DATA SHEET is required. However, the chemical, physical and toxicological properties of this product have not been thoroughly investigated. Therefore, please exercise due care when handling this material. This action is in compliance with State and Federal OSHA standards and regulations.

Quality Control: ____________

Date: February 26, 2022

**CSBio**
20 Kelly Court, Menlo Park, CA 94025 USA
T: (650) 322 1111 • F: (650) 322 2278
www.csbio.com • peptides@csbio.com

Compound: GT1314 Her3-Fc_ChTx1 Lot Number: X221 Expected M.W.: 3935.47 Found M.W.: 3934.20

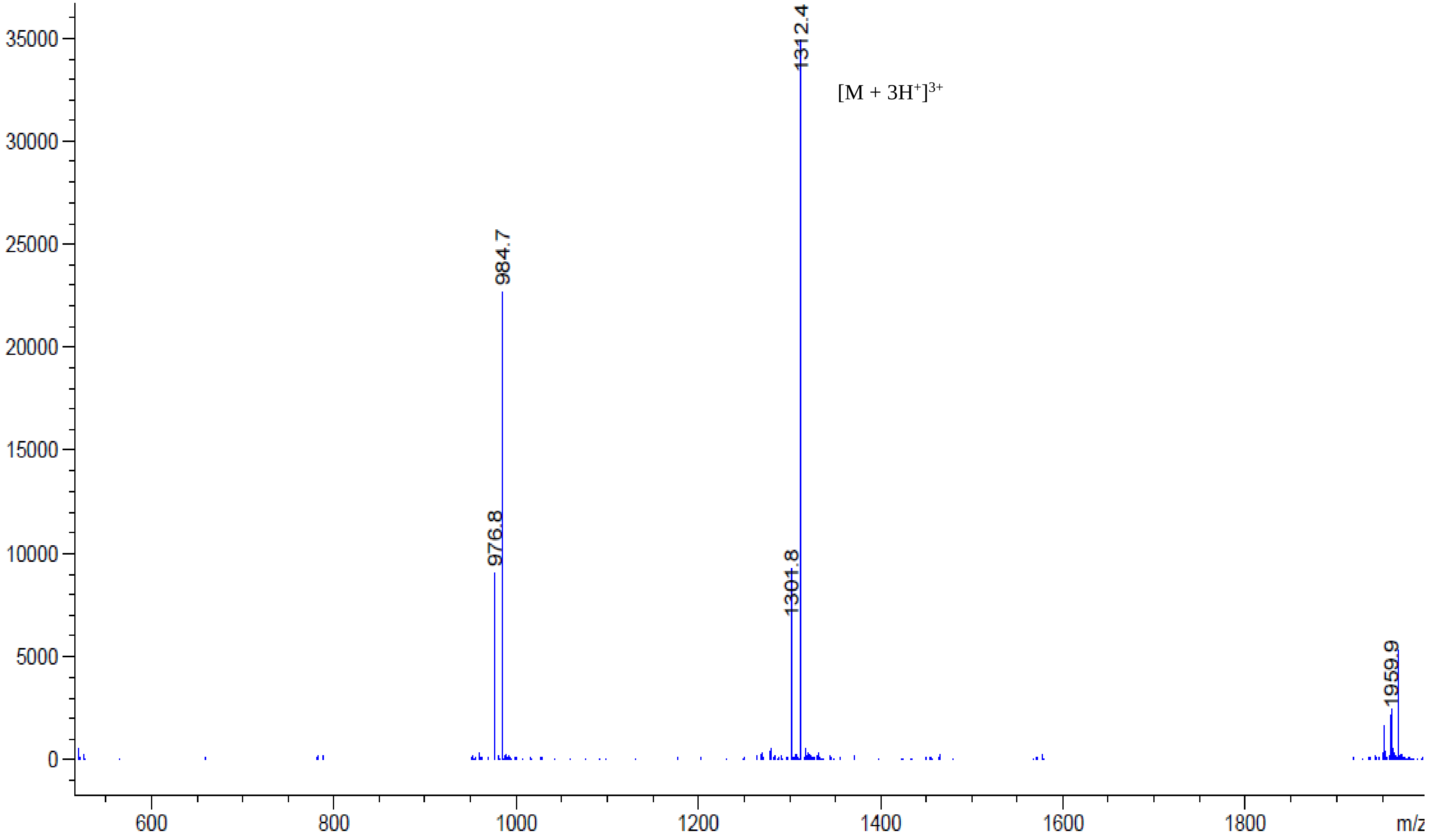

Sample Name: GT1314
Lot# X221
Instrument 1 Agilent 1290
Instrument ID: E195
Injection Date: 2/24/2022
Inj. Volume: 8.0 uL

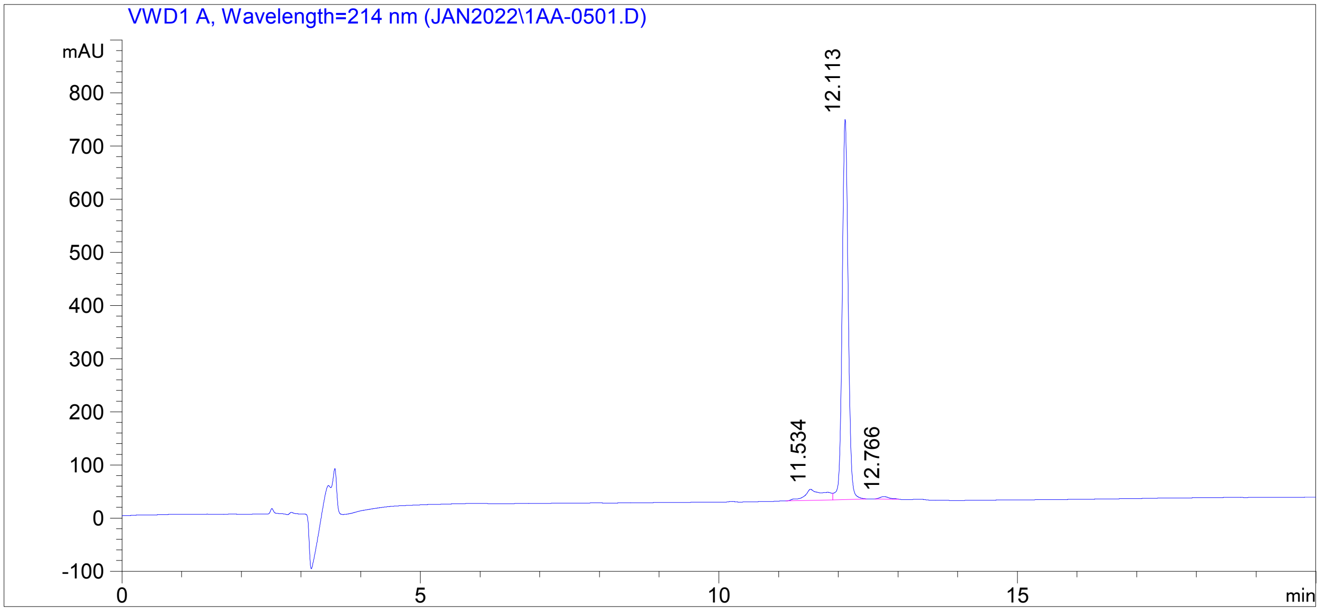


Data file name: C:\CHEM32\1\DATA\JAN2022\1AA-0501.D
Acq. Method: C:\Chem32\1\DATA\FEB2022\QC 2022-02-24 14-28-31\15-45-20.-1-2.M

Column: Phenomenex Luna C18 5u 100A 250x4.6mm
Buffer A: 0.1% TFA in Water
Buffer B: 0.1% TFA in ACN
Wavelength: 214 nm
Flow Rate: 1 ml/minute
Column Temperature: 25C
Gradient: 15% to 45% B in 20 minutes

| Peak # | RT [min] | Area | Height | Area % |
|---|---|---|---|---|
| 1 | 11.534 | 489.50 | 20.85 | 8.81 |
| 2 | 12.113 | 5015.04 | 715.86 | 90.31 |
| 3 | 12.766 | 48.82 | 4.54 | 0.88 |
