# Supplementary material for: Disulfide-constrained peptide scaffolds enable a robust peptide-therapeutic discovery platform
Source: PLoS One. 2024 Mar 28;19(3):e0300135. doi: 10.1371/journal.pone.0300135 (PMC10977697; doi:10.1371/journal.pone.0300135)
Supplement: S1 File — A zip file contains 51 pdf files with filenames are the same as the “DCP name” listed in the tables. (ZIP) [file pone.0300135.s004.zip › HtrA1_CBDwt46.pdf]

Quality Control Record

Product: HtrA1_CBDwt46
Sequence: Gly-Pro-Thr-Gln-Ser-His-Tyr-Gly-Gln-Cys-Gly-Gly-Trp-Gly-Tyr-Thr-Gly-Trp-Tyr-Gln-Cys-Ala-Ser-Gly-Thr-Thr-Cys-Gln-Gly-Pro-Arg-Ser-Arg-Leu-Ser-Gln-Cys-Leu-Pro-Gly-Ala-Ser

Note: Natural Oxidation

Product No.: GT1461 Expected M.W.: 4391.82 Found M.W.: 4390.80 Lot: X524

APPEARANCE:	White Powder
MOLECULAR WEIGHT VERIFICATION:	Confirmed
PURITY: Instrument: Agilent 1290<br>Condition: HPLC column in TFA System<br>Gradient: 20-50% Buffer B in 20 minutes<br>Buffer A: 0.1% TFA in H_2O<br>Buffer B: 0.1% TFA in ACN<br>Wavelength: 214 nm<br>Column: Phenomenex Luna C18 5µm 100Å, 4.6 x 250 mm	90.03%
PEPTIDE CONTENT:<br>(By N Elemental Analysis)	79.3%
ELLMAN'S TEST:	Complies
SUGGESTIONS FOR PEPTIDE DISSOLUTION:	0.1% TFA in Water
COUNTERIONS PRESENT:	TFA Salt
STORAGE:	All peptides should be stored dry at -20°C

This material is NOT FOR HUMAN USE. This material is not listed as hazardous by ∗NIOSH/RTECS. Therefore, no SAFETY DATA SHEET is required. However, the chemical, physical and toxicological properties of this product have not been thoroughly investigated. Therefore, please exercise due care when handling this material. This action is in compliance with State and Federal OSHA standards and regulations.

Quality Control: ____________

Date: June 7, 2022

CSBio
20 Kelly Court, Menlo Park, CA 94025 USA
T: (650) 322 1111 • F: (650) 322 2278
www.csbio.com • peptides@csbio.com

Compound: GT1461 HtrA1_CBDwt46 Lot Number: X524 Expected M.W.: 4391.82 Found M.W.: 4390.80

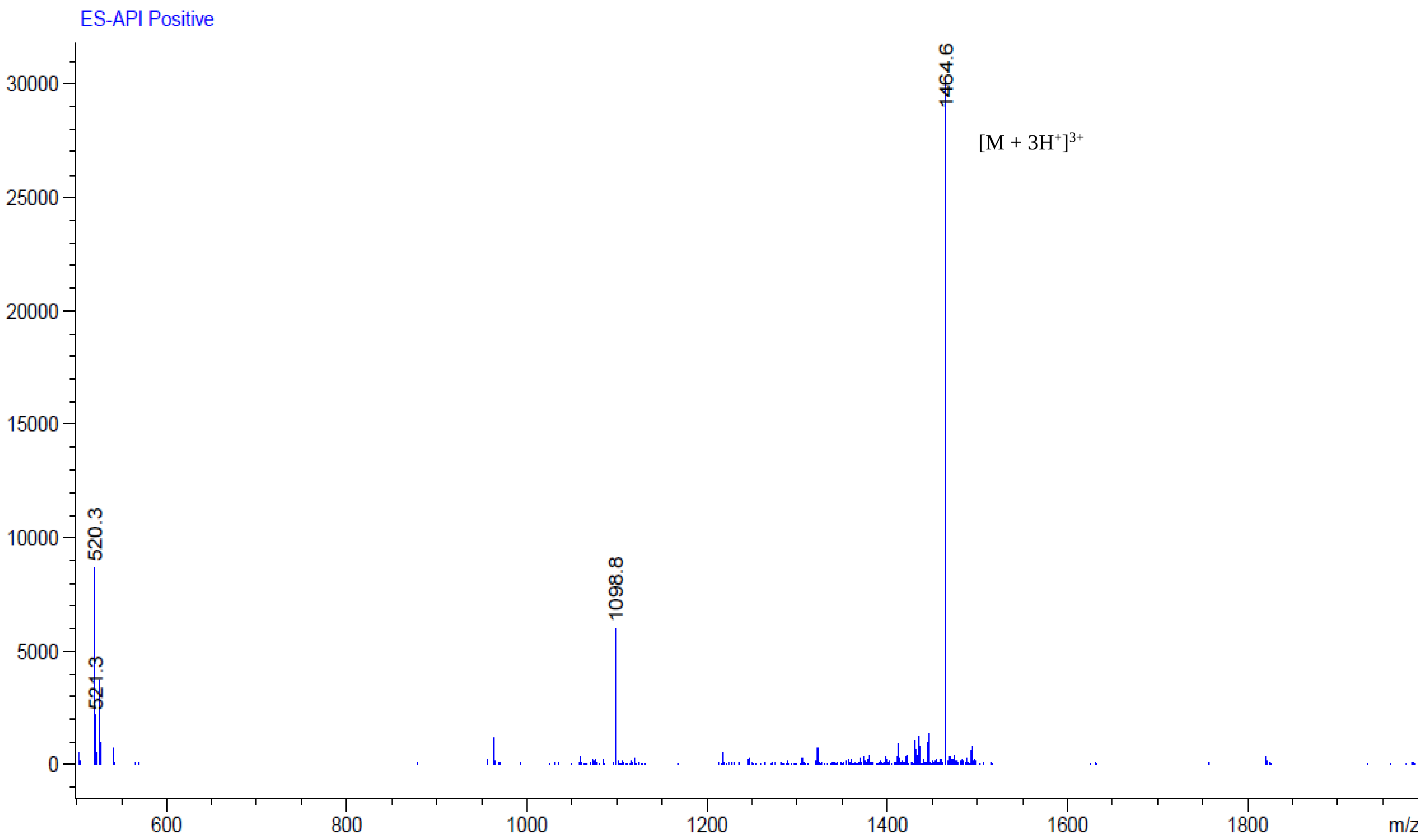

Sample Name: GT1461
Lot# X524
Instrument 1 Agilent 1290
Instrument ID: E195
Injection Date: 5/26/2022
Inj. Volume: 10.0 uL

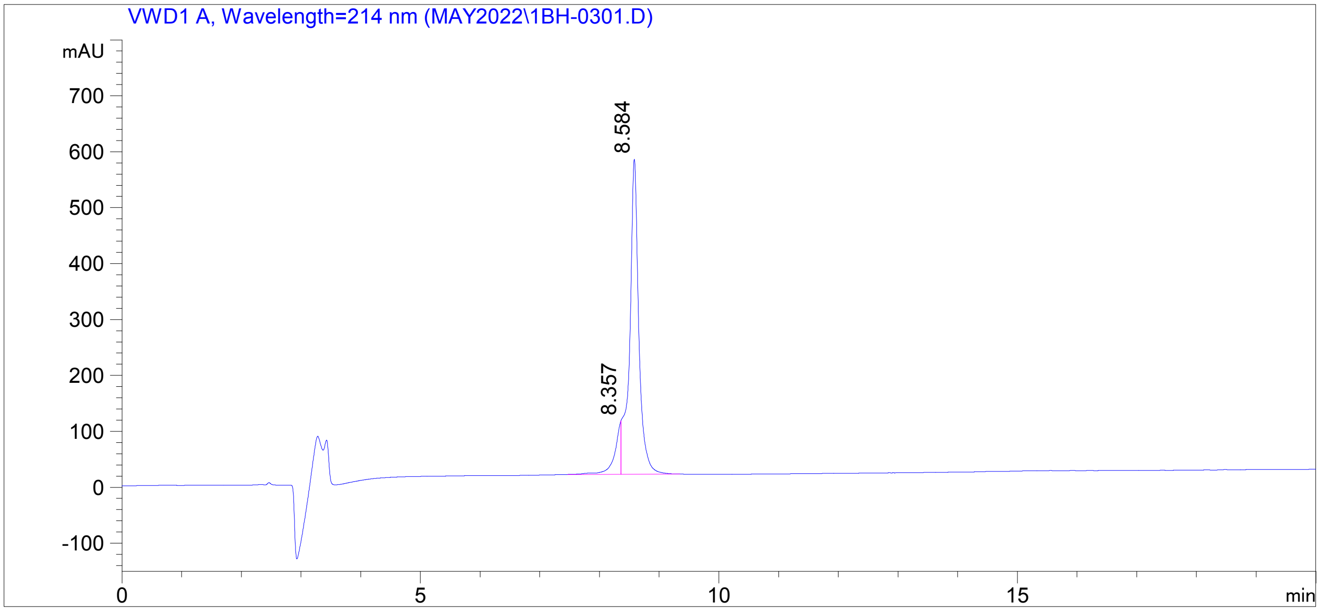

Data file name: C:\CHEM32\1\DATA\MAY2022\1BH-0301.D
Acq. Method: C:\Chem32\1\DATA\MAY2022\QC 2022-05-26 09-09-07\20-50-20-1-2.M

Column: Phenomenex Luna C18, 5um 250x4.6mm
Buffer A: 0.1% TFA in H2O
Buffer B: 0.1% TFA in ACN
Wavelength: 214 nm
Flow Rate: 1ml/minute
Column Temperature: 25C
Gradient: 20%-50% B in 20 minutes

Peak #	RT [min]	Area	Height	Area %
1	8.357	672.44	94.95	9.97
2	8.584	6072.28	563.31	90.03

Peptide Content with Elemental Analysis

Analysis: Determination of Peptide Content by Nitrogen Content
Instrument Model: Perkin Elmer Series II CHNS/O Analyser 2400
Sample Name: HtrA1_CBDwt46
Sample ID: GT1461
Lot Number: X524
Sample Testing Date: 6/6/2022

	N%
**Expected Content**	18.19
**Actual Content**	14.43
**Peptide Content (%)**	**79.3**

Performed by:

6/6/2022

Name Date

Reviewed by:

6/6/2022

Name Date

CSBio
20 Kelly Court, Menlo Park, CA 94025 USA
Tel: 650-322-1111 Fax: 650-322-2278 Web: www.csbio.com Email: peptides@csbio.com
