# Supplementary material for: Disulfide-constrained peptide scaffolds enable a robust peptide-therapeutic discovery platform
Source: PLoS One. 2024 Mar 28;19(3):e0300135. doi: 10.1371/journal.pone.0300135 (PMC10977697; doi:10.1371/journal.pone.0300135)
Supplement: S1 File — A zip file contains 51 pdf files with filenames are the same as the “DCP name” listed in the tables. (ZIP) [file pone.0300135.s004.zip › HtrA1_CirA38.pdf]

# Quality Control Record

Product: HtrA1_CirA38V
Sequence: Trp-Ile-Pro-Cys-Ile-Ser-Ala-Ala-Leu-Gly-Cys-Ser-Cys-Lys-Asn-Lys-Val-Cys-Tyr-Phe-Leu-Tyr-Gln-Ile-Cys-Gly-Glu-Ser-Cys-Val

Note: Natural Oxidation

Product No.: GT1495 Expected M.W.: 3296.98 Found M.W.: 3296.40 Lot: X545

APPEARANCE: White Powder

MOLECULAR WEIGHT VERIFICATION: Confirmed

PURITY: Instrument: Agilent 1200 — 90.03%
Condition: HPLC column in TFA System
Gradient: 30-60% Buffer B in 20 minutes
Buffer A: 0.1% TFA in $H_2O$
Buffer B: 0.1% TFA in ACN
Wavelength: 214 nm
Column: Phenomenex Luna C18 5µm 100Å, 4.6 x 250 mm

PEPTIDE CONTENT: 87.0%
(By N Elemental Analysis)

ELLMAN'S TEST: Complies

SUGGESTIONS FOR PEPTIDE DISSOLUTION: 0.1% TFA in Water

COUNTERIONS PRESENT: TFA Salt

STORAGE: All peptides should be stored dry at -20°C

This material is NOT FOR HUMAN USE. This material is not listed as hazardous by ∗NIOSH/RTECS. Therefore, no SAFETY DATA SHEET is required. However, the chemical, physical and toxicological properties of this product have not been thoroughly investigated. Therefore, please exercise due care when handling this material. This action is in compliance with State and Federal OSHA standards and regulations.

Quality Control: ____________________

Date: June 23, 2022

**CSBio**
20 Kelly Court, Menlo Park, CA 94025 USA
T: (650) 322 1111 • F: (650) 322 2278
www.csbio.com • peptides@csbio.com

Compound: GT1495 HtrA1_CirA38V Lot Number: X545 Expected M.W.: 3296.98 Found M.W.: 3296.40

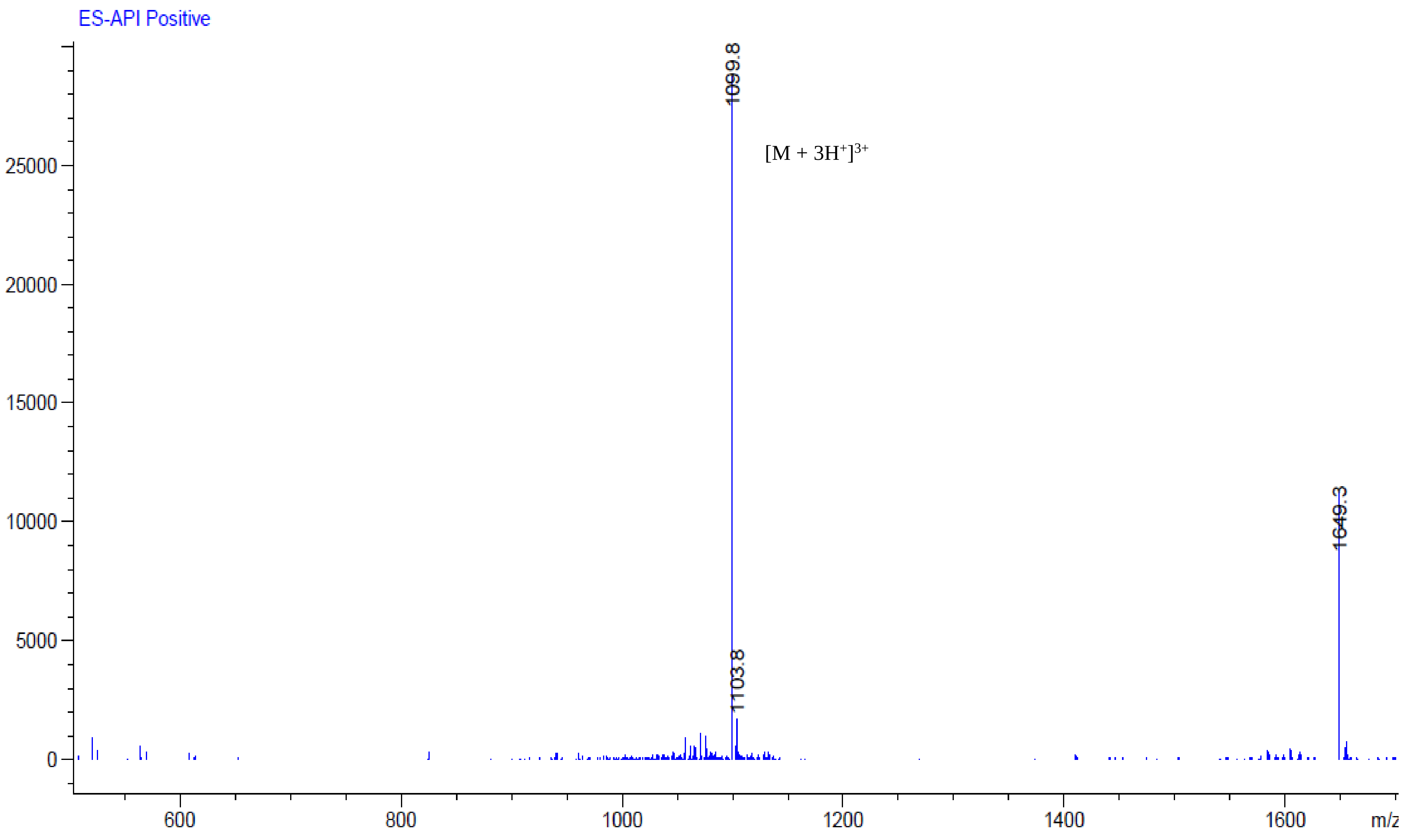

Sample Name: GT1495
Lot# X545
Instrument 1 Agilent 1200
Instrument ID: E645
Injection Date: 5/31/2022
Inj. Volume: 100.0uL

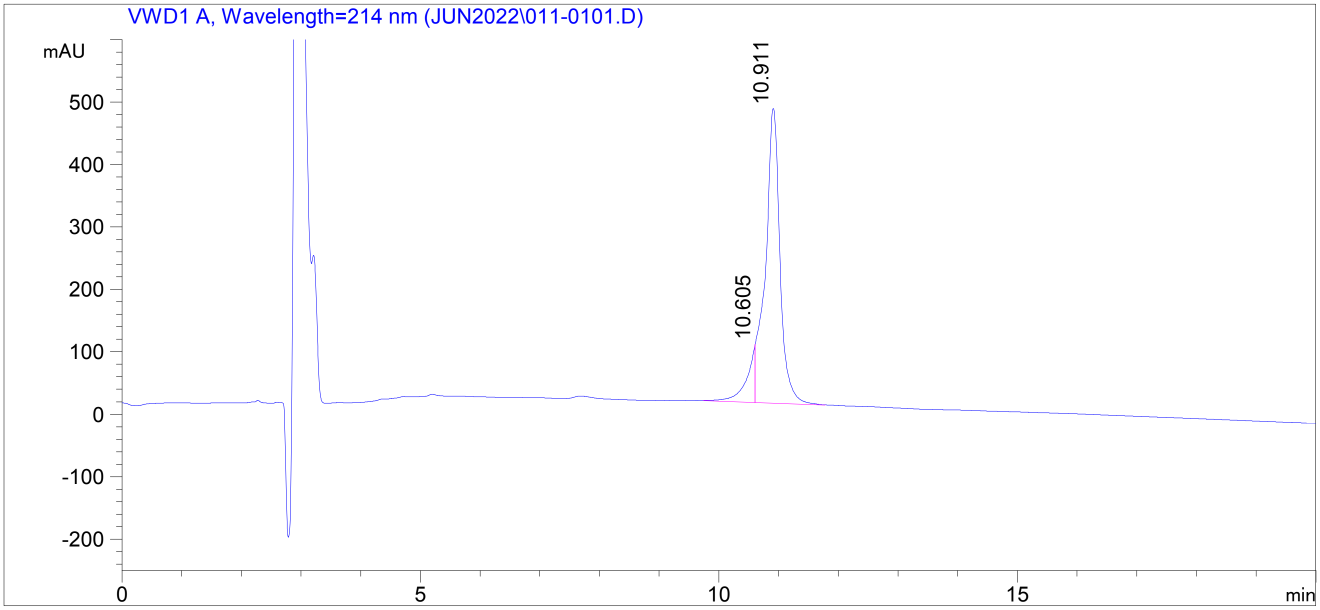


Data file name: C:\CHEM32\1\DATA\JUN2022\011-0101.D
Acq. Method: C:\Chem32\1\DATA\MAY2022\WG20202022-05-3109-42-41\30-60-20.M

Column: Phenomenex Luna C18 (2) 5u 100A, 250x4.6mm
Buffer A: 0.1% TFA in H2O
Buffer B: 0.1% TFA in ACN
Wavelength: 214 nm
Flow Rate: 1 ml/minute
Column Temperature: 25C
Gradient: 30-60% B in 20 minutes

| Peak # | RT [min] | Area | Height | Area % |
|---|---|---|---|---|
| 1 | 10.605 | 889.97 | 91.83 | 9.97 |
| 2 | 10.911 | 8038.98 | 472.22 | 90.03 |

# Peptide Content with Elemental Analysis

**Analysis:** Determination of Peptide Content by Nitrogen Content
**Instrument Model:** Perkin Elmer Series II CHNS/O Analyser 2400
**Sample Name:** HtrA1_CirA38V
**Sample ID:** GT1495
**Lot Number:** X545
**Sample Testing Date:** 6/11/2022

| | N% |
|---|---|
| **Expected Content** | 14.87 |
| **Actual Content** | 12.94 |
| **Peptide Content (%)** | **87.0** |

Performed by:

6/11/2022

Name Date

Reviewed by:

6/11/2022

Name Date

CSBio
20 Kelly Court, Menlo Park, CA 94025 USA
Tel: 650-322-1111 Fax: 650-322-2278 Web: www.csbio.com Email: peptides@csbio.com
