# Supplementary material for: Disulfide-constrained peptide scaffolds enable a robust peptide-therapeutic discovery platform
Source: PLoS One. 2024 Mar 28;19(3):e0300135. doi: 10.1371/journal.pone.0300135 (PMC10977697; doi:10.1371/journal.pone.0300135)
Supplement: S1 File — A zip file contains 51 pdf files with filenames are the same as the “DCP name” listed in the tables. (ZIP) [file pone.0300135.s004.zip › N2L-EET-03.pdf]

SAMPLE TEST REPORT

Product: N2L-EET-03 Gly-28-Gly
Sequence: Gly-Cys-Gly-Trp-Thr-Leu-Trp-His-Cys-Lys-Gln-Asp-Ser-Asp-Cys-Leu-Ala-Gly-Cys-Val-Cys-Glu-Pro-Ile-Trp-Trp-Cys-Gly

Note: Natural Oxidation

Product No.: GT0290 Expected M.W.: 3151.64 Found M.W.: 3152.96 Lot: U070

APPEARANCE:	White Powder
MOLECULAR WEIGHT VERIFICATION:	Confirmed
PURITY: Instrument: Agilent 1260 System<br>Condition: HPLC column in TFA System<br>Gradient: 20-50% Buffer B in 20 minutes<br>Buffer A: 0.1% TFA in H_2O<br>Buffer B: 0.1% TFA in ACN<br>Wavelength: 214 nm<br>Column: Phenomenex Luna C18 5µm 100Å, 4.6 x 250 mm	95.26%
ELLMAN'S TEST:	Complies
PEPTIDE CONTENT:<br>(By Amino Acid Analysis)	83.4%
SUGGESTIONS FOR PEPTIDE DISSOLUTION:	Acetonitrile / 0.1% TFA in Water
COUNTERIONS PRESENT:	TFA Salt
STORAGE:	All peptides should be stored dry at -20°C

This material is not listed as hazardous by ∗NIOSH/RTECS. Therefore, no SAFETY DATA SHEET is required. However, the chemical, physical and toxicological properties of this product have not been thoroughly investigated. Therefore, please exercise due care when handling this material. This action is in compliance with State and Federal OSHA standards and regulations.

Quality Control: Xiaosong Jin

Date: October 4, 2018

CS Bio Co.
20 Kelly Court, Menlo Park, CA 94025 USA
T: (650) 322 1111 • F: (650) 322 2278
www.csbio.com • peptides@csbio.com

Compound: GT0290 N2L-EET-03 Gly-28-Gly Lot Number: U070 Expected M.W.: 3151.64 Found M.W.: 3152.96

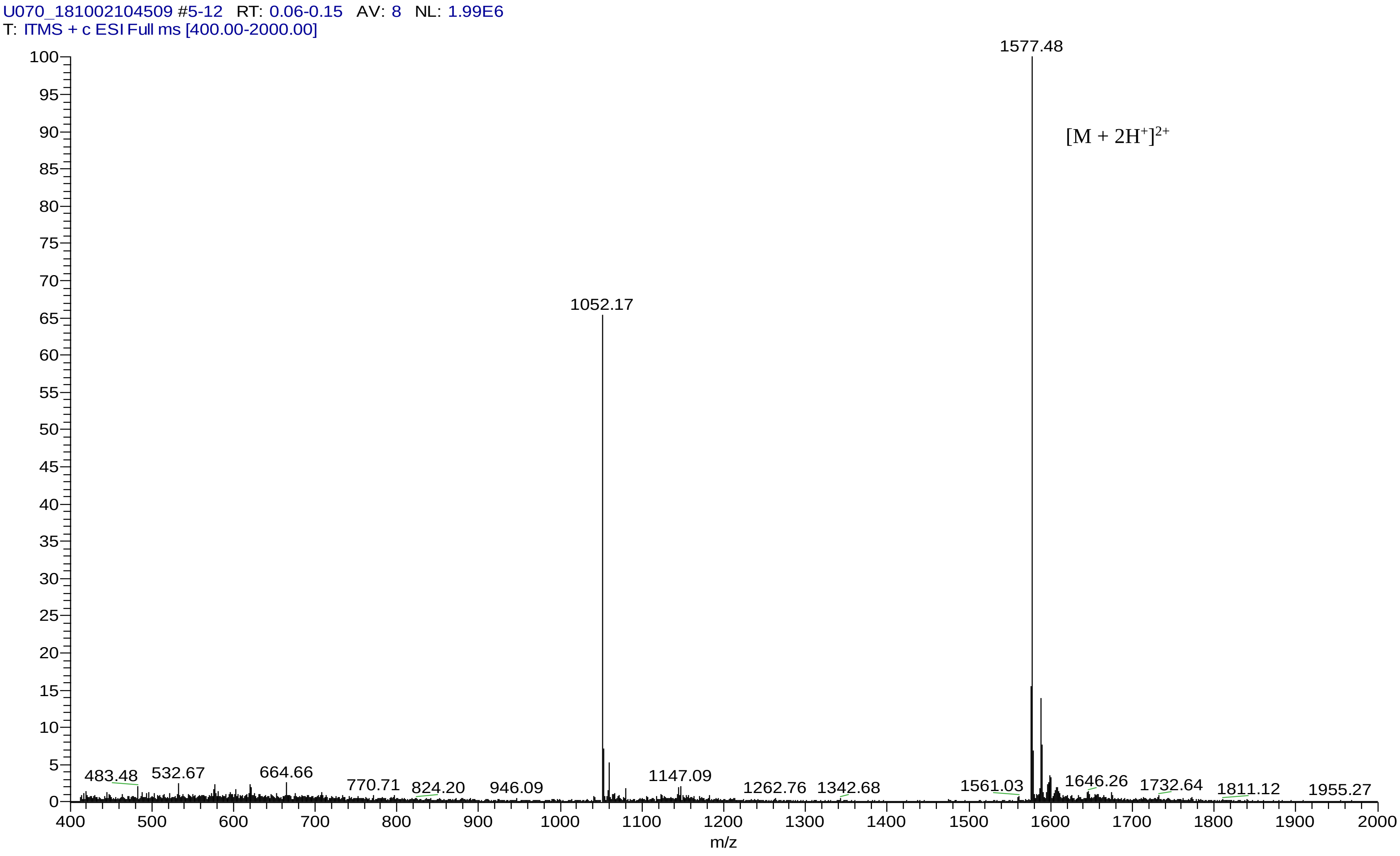

Sample Name: GT0290
Lot#: U070
Instrument 1 Agilent 1260
Instrument ID: RD-HPLC 1
Injection Date: 10/2/2018
Inj. Volume: 30.0 uL

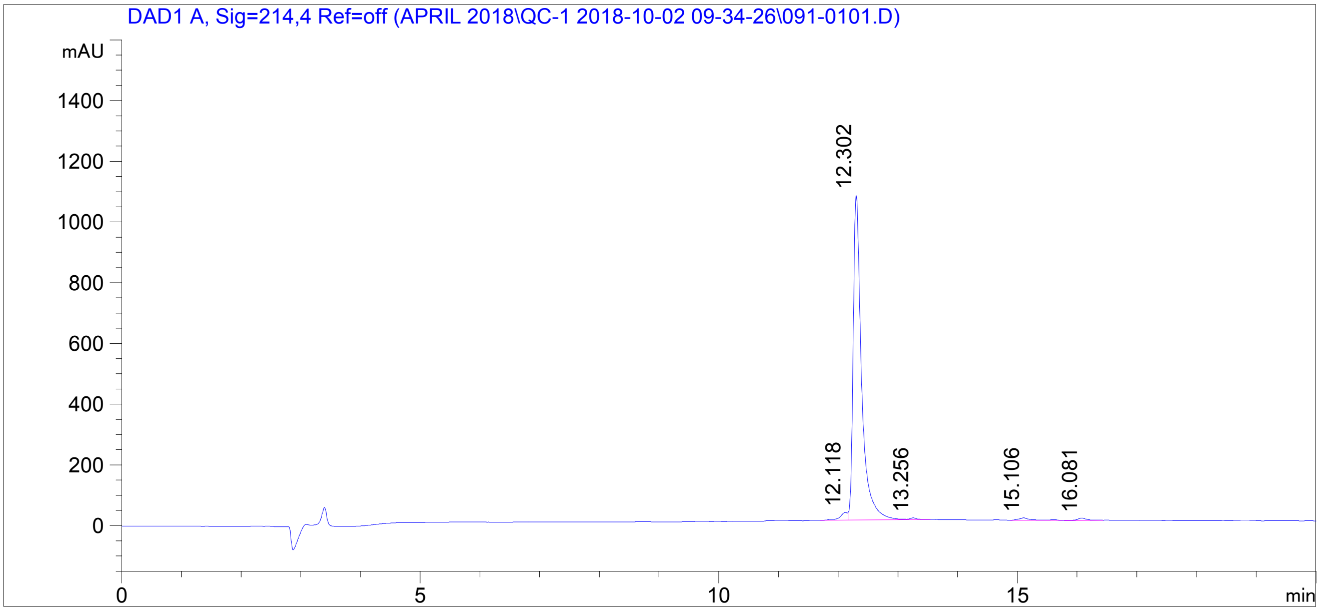

Data file name: C:\CHEM32\1\DATA\APRIL 2018\QC-1 2018-10-02 09-34-26\091-0101.D

Acq. Method: C:\Chem32\1\DATA\April 2018\QC-1 2018-10-02 09-34-26\20-50-20.M

Column: Phenomenex Luna C18, 5um 250 x 4.6mm
Buffer A: 0.1% TFA in H2O
Buffer B: 0.1% TFA in ACN
Flow Rate: 1ml/min
Gradient: 20-50% B in 20 min

Peak #	RT [min]	Area	Height	Area %
1	12.118	236.73	25.35	2.15
2	12.302	10474.88	1071.49	95.26
3	13.256	63.12	5.70	0.57
4	15.106	135.99	8.29	1.24
5	16.081	84.81	7.61	0.77

Peptide Content Determination by Amino Acid Analysis

Instrument Model: Waters H Class System
Sample Name: N2L-EET-03 Gly-28-Gly
Sample ID: GT0290
Lot No.: U070
Sample Testing Date: 10/04/2018

**Peptide Content (%)**	**83.4%**

Performed by:

Shilpa Patel 10/04/2018

Name Date

Reviewed by:

Xiaosong Jin 10/04/2018

Name Date
