# Supplementary material for: Disulfide-constrained peptide scaffolds enable a robust peptide-therapeutic discovery platform
Source: PLoS One. 2024 Mar 28;19(3):e0300135. doi: 10.1371/journal.pone.0300135 (PMC10977697; doi:10.1371/journal.pone.0300135)
Supplement: S1 File — A zip file contains 51 pdf files with filenames are the same as the “DCP name” listed in the tables. (ZIP) [file pone.0300135.s004.zip › N2L-EET-28.pdf]

SAMPLE TEST REPORT

Product: N2L-EET-28 Gly-30-Gly
Sequence: Gly-Cys-Lys-Gln-Phe-Gly-Asn-Gln-Trp-Ile-Cys-Lys-Gln-Asp-Ser-Asp-Cys-Leu-Ala-Gly-Cys-Val-Cys-Gly-Pro-Asn-Gly-Phe-Cys-Gly

Note: Natural Oxidation

Product No.: GT0291 Expected M.W.: 3133.58 Found M.W.: 3134.31 Lot: U072

APPEARANCE: White Powder

MOLECULAR WEIGHT VERIFICATION: Confirmed

PURITY: Instrument: Agilent 1260 System
Condition: HPLC column in TFA System
Gradient: 15-45% Buffer B in 20 minutes
Buffer A: 0.1% TFA in H_2O
Buffer B: 0.1% TFA in ACN
Wavelength: 214 nm
Column: Phenomenex Luna C18 5µm 100Å, 4.6 x 250 mm

90.93% (Before Lyophilization)
89.50% (After Lyophilization)

ELLMAN'S TEST: Complies

PEPTIDE CONTENT: 84.3%
(By Amino Acid Analysis)

SUGGESTIONS FOR PEPTIDE DISSOLUTION: Acetonitrile / 0.1% TFA in Water

COUNTERIONS PRESENT: TFA Salt

STORAGE: All peptides should be stored dry at -20°C

This material is not listed as hazardous by ∗NIOSH/RTECS. Therefore, no SAFETY DATA SHEET is required. However, the chemical, physical and toxicological properties of this product have not been thoroughly investigated. Therefore, please exercise due care when handling this material. This action is in compliance with State and Federal OSHA standards and regulations.

Quality Control: Xiaosong Jin

Date: October 8, 2018

CS Bio Co.
20 Kelly Court, Menlo Park, CA 94025 USA
T: (650) 322 1111 • F: (650) 322 2278
www.csbio.com • peptides@csbio.com

Compound: GT0291 N2L-EET-28 Gly-30-Gly Lot Number: U072 Expected M.W.: 3133.58 Found M.W.: 3134.31

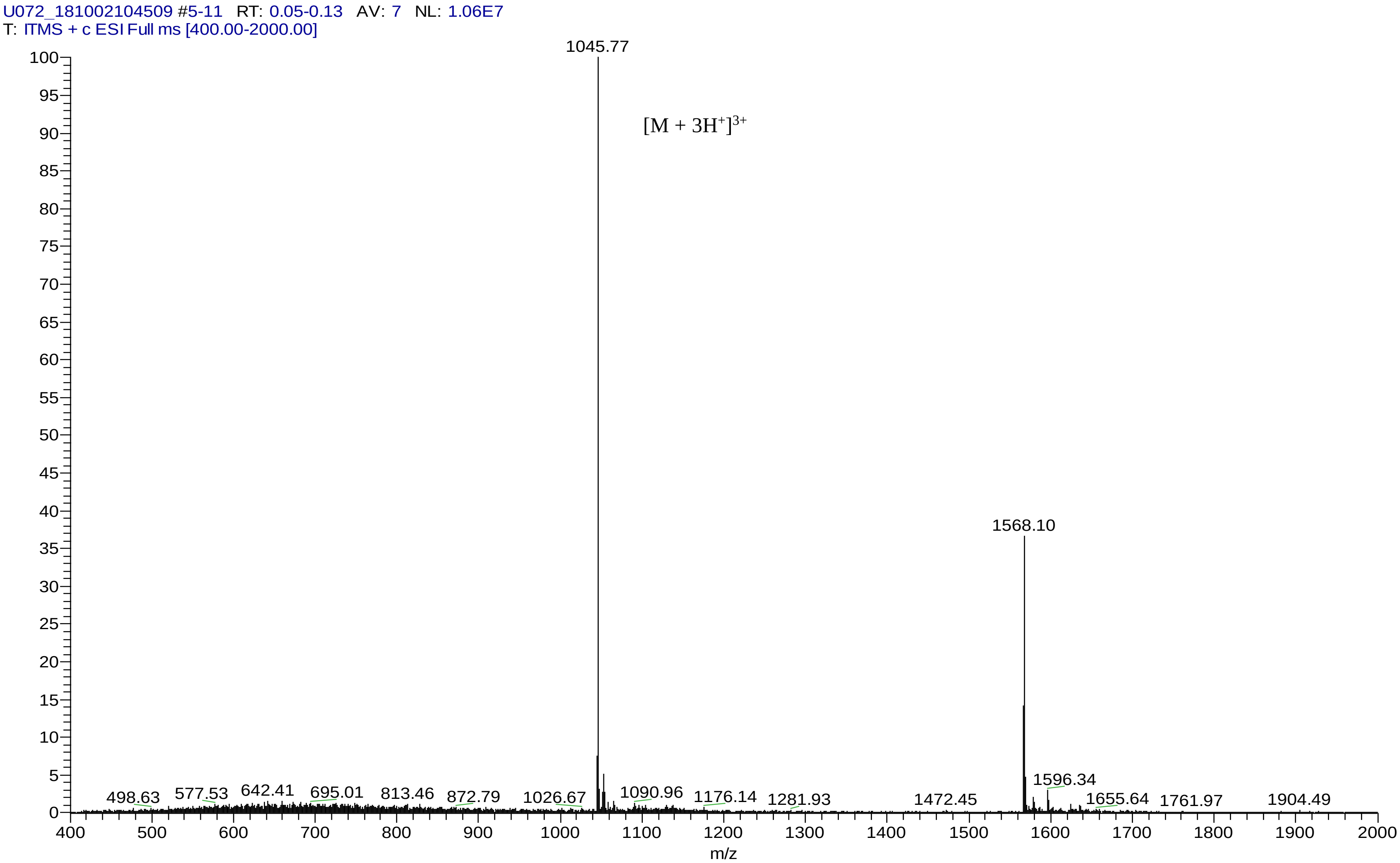

Sample Name: GT0291 (Before Lyophilization)
Lot#: U072
Instrument 1 Agilent 1260
Instrument ID: RD-HPLC 1
Injection Date: 9/29/2018
Inj. Volume: 10.0 uL

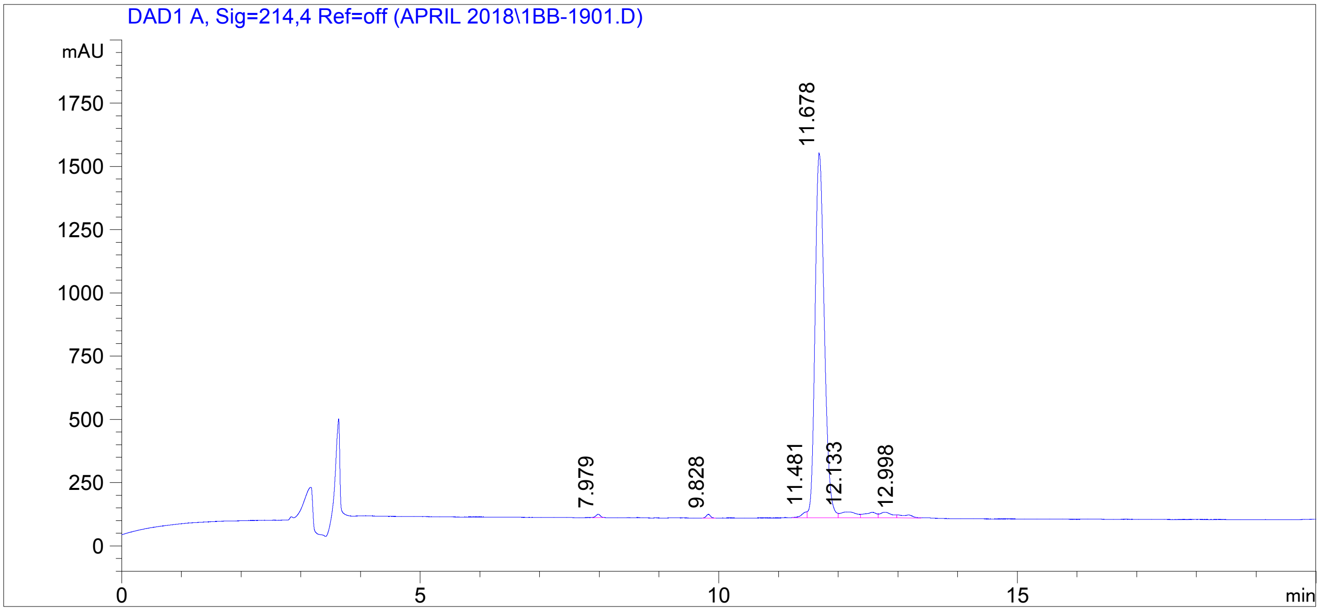

Data file name: C:\CHEM32\1\DATA\APRIL 2018\1BB-1901.D

Acq. Method: C:\Chem32\1\DATA\Sep2018\PURIFICATION 2018-09-29 11-30-03\15-45-20-1-6.M
Column: Phenomenex Luna C18 5u 100A 250x4.6mm P/N: 00G-4252-E0
Buffer A: 0.1% TFA in Water
Buffer B: 0.1% TFA in ACN
Flow Rate: 1 ml/min
Gradient: 15 to 45% B in 20 min

Peak #	RT [min]	Area	Height	Area %
1	7.979	75.07	13.62	0.44
2	9.828	74.79	15.18	0.44
3	11.481	147.00	24.00	0.86
4	11.678	15521.81	1443.19	90.93
5	12.133	441.88	23.46	2.59
6	12.577	309.03	22.32	1.81
7	12.783	314.87	22.59	1.84
8	12.998	186.49	12.25	1.09

Sample Name: GT0291
Lot#: U072
Instrument 1 Agilent 1260
Instrument ID: RD-HPLC 1
Injection Date: 10/2/2018
Inj. Volume: 30.0 uL

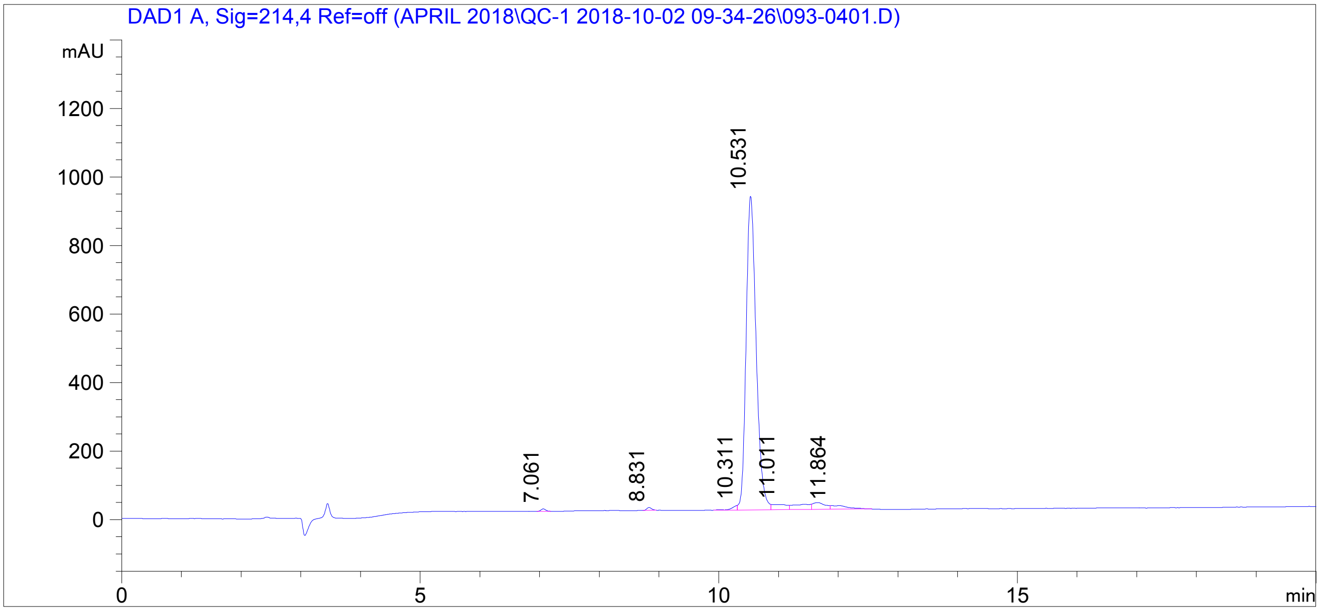

Data file name: C:\CHEM32\1\DATA\APRIL 2018\QC-1 2018-10-02 09-34-26\093-0401.D

Acq. Method: C:\Chem32\1\DATA\April 2018\QC-1 2018-10-02 09-34-26\15-45-20.M

Column: Phenomenex Luna C18 5u 100A 250x4.6mm P/N: 00G-4252-E0
Buffer A: 0.1% TFA in Water
Buffer B: 0.1% TFA in ACN
Flow Rate: 1 ml/min
Gradient: 15 to 45% B in 20 min

Peak #	RT [min]	Area	Height	Area %
1	7.061	42.54	7.32	0.36
2	8.831	49.07	8.55	0.41
3	10.311	88.35	14.03	0.74
4	10.531	10720.13	915.77	89.50
5	11.011	265.55	15.07	2.22
6	11.434	307.95	15.40	2.57
7	11.652	290.63	19.72	2.43
8	11.864	213.31	10.97	1.78

Peptide Content Determination by Amino Acid Analysis

Instrument Model: Waters H Class System
Sample Name: N2L-EET-28 Gly-30-Gly
Sample ID: GT0291
Lot No.: U072
Sample Testing Date: 10/04/2018

**Peptide Content (%)**	**84.3%**

Performed by:

Shilpa Patel. 10/04/2018

Name Date

Reviewed by:

Xiaosong Jin 10/04/2018

Name Date
