# Supplementary material for: Disulfide-constrained peptide scaffolds enable a robust peptide-therapeutic discovery platform
Source: PLoS One. 2024 Mar 28;19(3):e0300135. doi: 10.1371/journal.pone.0300135 (PMC10977697; doi:10.1371/journal.pone.0300135)
Supplement: S1 File — A zip file contains 51 pdf files with filenames are the same as the “DCP name” listed in the tables. (ZIP) [file pone.0300135.s004.zip › N2L-EET-29.pdf]

SAMPLE TEST REPORT

Product: N2L-EET-29 Gly-28-Gly
Sequence: Gly-Cys-Gly-Trp-Thr-Leu-Ala-His-Cys-Lys-Gln-Asp-Ser-Asp-Cys-Leu-Ala-Gly-Cys-Val-Cys-Gln-Pro-Arg-Trp-Val-Cys-Gly

Note: Natural Oxidation

Product No.: GT0292 Expected M.W.: 2991.47 Found M.W.: 2991.86 Lot: U089

APPEARANCE: White Powder

MOLECULAR WEIGHT VERIFICATION: Confirmed

PURITY: Instrument: Agilent 1260 System — 95.26% (Before Lyophilization)
Condition: HPLC column in TFA System
Gradient: 20-50% Buffer B in 20 minutes
Buffer A: 0.1% TFA in H_2O
Buffer B: 0.1% TFA in ACN
Wavelength: 214 nm
Column: Phenomenex Luna C18 5µm 100Å, 4.6 x 250 mm

PURITY: Instrument: Agilent 1260 System — 92.60% (After Lyophilization)
Condition: HPLC column in TFA System
Gradient: 20-50% Buffer B in 20 minutes
Buffer A: 0.1% TFA in H2O
Buffer B: 0.1% TFA in ACN
Wavelength: 214 nm
Column: Phenomenex Luna C18 5µm 100Å, 4.6 x 250 mm

ELLMAN’S TEST: Complies

CS Bio Co.
20 Kelly Court, Menlo Park, CA 94025 USA
T: (650) 322 1111 • F: (650) 322 2278
www.csbio.com • peptides@csbio.com

SAMPLE TEST REPORT

Product: N2L-EET-29 Gly-28-Gly

Sequence: Gly-Cys-Gly-Trp-Thr-Leu-Ala-His-Cys-Lys-Gln-Asp-Ser-Asp-Cys-Leu-Ala-Gly-Cys-Val-Cys-Gln-Pro-Arg-Trp-Val-Cys-Gly

Note: Natural Oxidation

Product No.: GT0292 Expected M.W.: 2991.47 Found M.W.: 2991.86 Lot: U089

PEPTIDE CONTENT: 82.8%
(By Amino Acid Analysis)

SUGGESTIONS FOR PEPTIDE DISSOLUTION: Acetonitrile / 0.1% TFA in Water

COUNTERIONS PRESENT: TFA Salt

STORAGE: All peptides should be stored dry at -20°C

This material is not listed as hazardous by *NIOSH/RTECS. Therefore, no SAFETY DATA SHEET is required. However, the chemical, physical and toxicological properties of this product have not been thoroughly investigated. Therefore, please exercise due care when handling this material. This action is in compliance with State and Federal OSHA standards and regulations.

Quality Control: Xiaosong Jin

Date: October 18, 2018

CS Bio Co.
20 Kelly Court, Menlo Park, CA 94025 USA
T: (650) 322 1111 • F: (650) 322 2278
www.csbio.com • peptides@csbio.com

Compound: GT0292 N2L-EET-29 Gly-28-Gly Lot Number: U089 Expected M.W.: 2991.47 Found M.W.: 2991.86

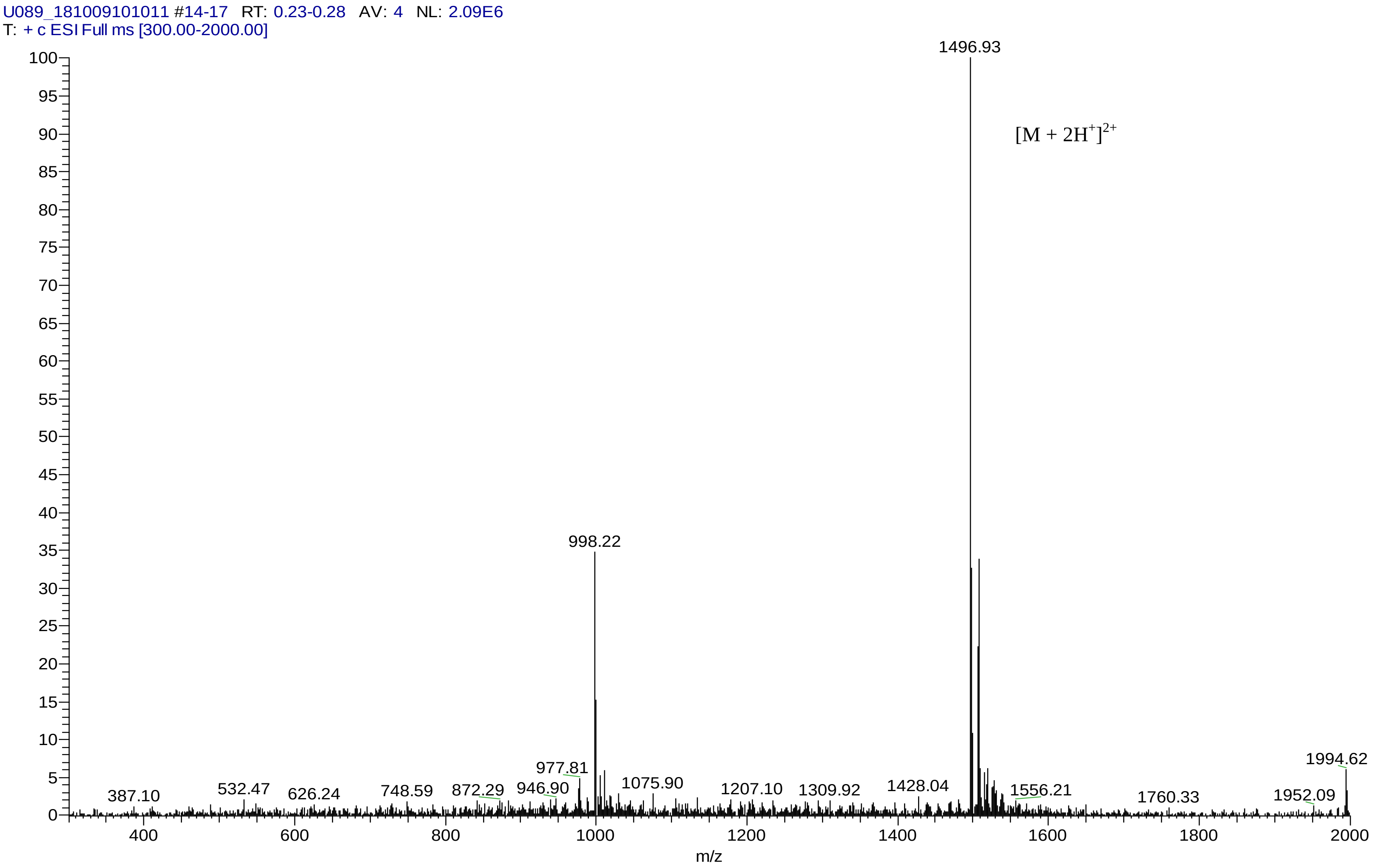

Sample Name: GT0292 (Before Lyophilization)
Lot#: U089
Instrument 1 Agilent 1260
Instrument ID: RD-HPLC 1
Injection Date: 10/6/2018
Inj. Volume: 12.0 uL

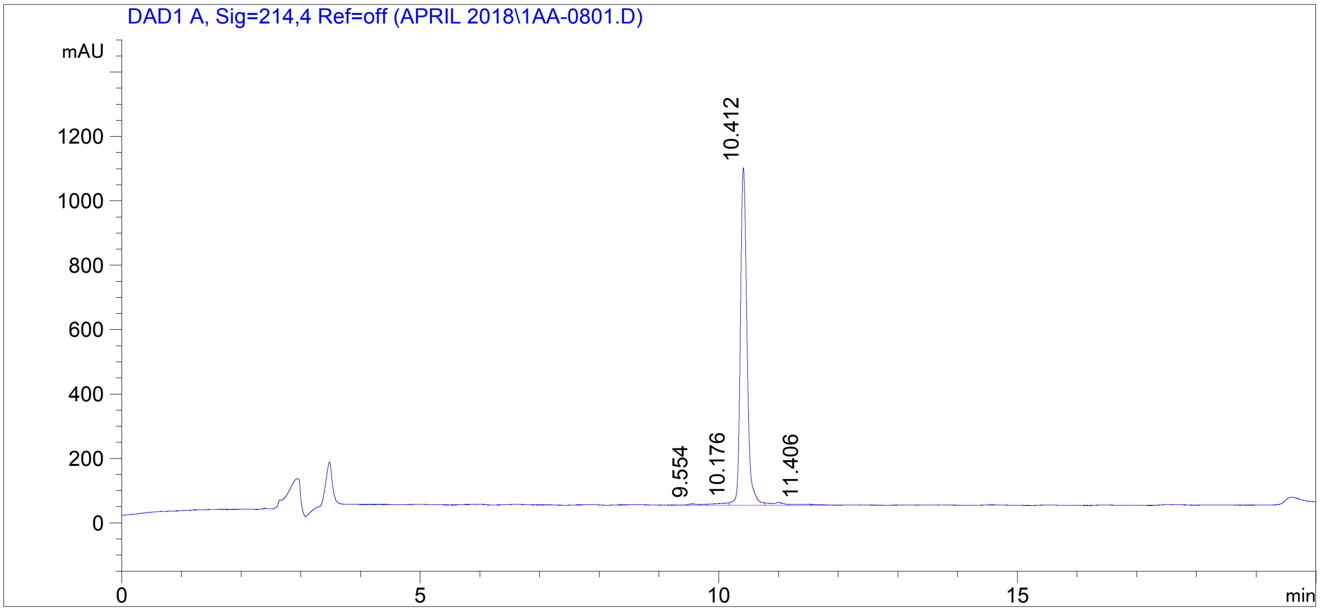

Data file name: C:\CHEM32\1\DATA\APRIL 2018\1AA-0801.D

Acq. Method: C:\Chem32\1\DATA\Oct2018\PURIFICATION 2018-10-06 08-47-05\20-50-20-1-6.M
Column: Phenomenex Luna C18, 5um 250 x 4.6mm
Buffer A: 0.1% TFA in H2O
Buffer B: 0.1% TFA in ACN
Flow Rate: 1ml/min
Gradient: 20-50% B in 20 min

Peak #	RT [min]	Area	Height	Area %
1	9.554	49.46	4.13	0.59
2	10.176	127.58	8.00	1.52
3	10.412	8012.36	1048.43	95.26
4	11.010	137.78	8.66	1.64
5	11.406	83.55	2.92	0.99

Sample Name: GT0292
Lot#: U089
Instrument 1 Agilent 1260
Instrument ID: RD-HPLC 1
Injection Date: 10/9/2018
Inj. Volume: 20.0 uL

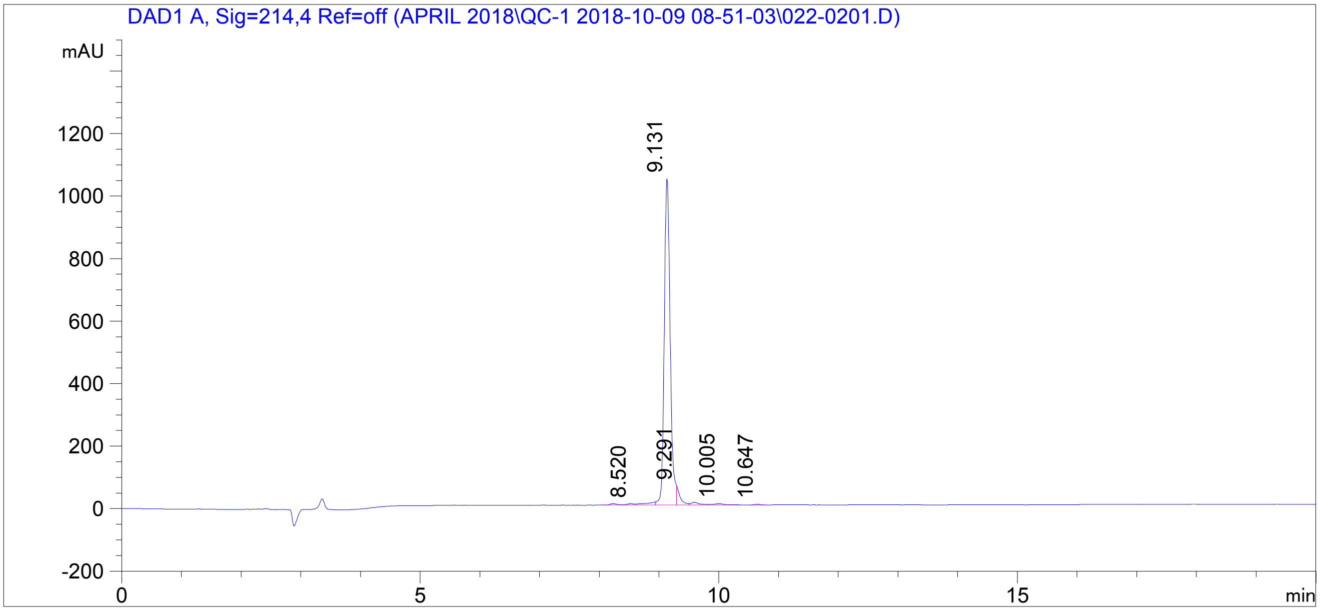

Data file name: C:\CHEM32\1\DATA\APRIL 2018\QC-1 2018-10-09 08-51-03\022-0201.D

Acq. Method: C:\Chem32\1\DATA\April 2018\QC-1 2018-10-09 08-51-03\20-50-20.M

Column: Phenomenex Luna C18, 5um 250 x 4.6mm
Buffer A: 0.1% TFA in H2O
Buffer B: 0.1% TFA in ACN
Flow Rate: 1ml/min
Gradient: 20-50% B in 20 min

Peak #	RT [min]	Area	Height	Area %
1	8.229	37.84	4.65	0.49
2	8.520	38.34	4.21	0.50
3	8.938	111.53	10.17	1.44
4	9.131	7169.36	1045.59	92.60
5	9.291	237.84	62.84	3.07
6	9.592	80.79	8.62	1.04
7	10.005	51.87	4.00	0.67
8	10.647	14.95	2.16	0.19

Peptide Content Determination by Amino Acid Analysis

**Instrument Model:**	Waters H Class System
**Sample Name:**	N2L-EET-29 Gly-28-Gly
**Sample ID:**	GT0292
**Lot No.:**	U089
**Sample Testing Date:**	10/15/2018

**Peptide Content (%)**	**82.8%**

Performed by:

Shilpa Patel. 10/15/2018

Name Date

Reviewed by:

Xiaosong Jin 10/15/2018

Name Date
