# Supplementary material for: Disulfide-constrained peptide scaffolds enable a robust peptide-therapeutic discovery platform
Source: PLoS One. 2024 Mar 28;19(3):e0300135. doi: 10.1371/journal.pone.0300135 (PMC10977697; doi:10.1371/journal.pone.0300135)
Supplement: S1 File — A zip file contains 51 pdf files with filenames are the same as the “DCP name” listed in the tables. (ZIP) [file pone.0300135.s004.zip › N2L-EET-36.pdf]

SAMPLE TEST REPORT

Product: N2L-EET-36 Gly-28-Gly
Sequence: Gly-Cys-Glu-Val-Val-Met-Glu-Arg-Cys-Lys-Gln-Asp-Ser-Asp-Cys-Leu-Ala-Gly-Cys-Val-Cys-Trp-Tyr-Trp-Thr-Ser-Cys-Gly

Note: Natural Oxidation

Product No.: GT0289 Expected M.W.: 3126.61 Found M.W.: 3215.90 Lot: U343

APPEARANCE: White Powder

MOLECULAR WEIGHT VERIFICATION: Confirmed

PURITY: Instrument: Agilent 1260 System — 80.70% (Before Lyophilization)
Condition: HPLC column in TFA System
Gradient: 20-50% Buffer B in 20 minutes
Buffer A: 0.1% TFA in H_2O
Buffer B: 0.1% TFA in ACN
Wavelength: 214 nm
Column: Phenomenex Luna C18 5µm 100Å, 4.6 x 250 mm
Temperature: 60°C

PURITY: Instrument: Agilent 1260 System — 47.58% (After Lyophilization)
Condition: HPLC column in TFA System
Gradient: 20-50% Buffer B in 20 minutes
Buffer A: 0.1% TFA in H2O
Buffer B: 0.1% TFA in ACN
Wavelength: 214 nm
Column: Phenomenex Luna C18 5µm 100Å, 4.6 x 250 mm

ELLMAN'S TEST: Complies

CS Bio Co.
20 Kelly Court, Menlo Park, CA 94025 USA
T: (650) 322 1111 • F: (650) 322 2278
www.csbio.com • peptides@csbio.com

SAMPLE TEST REPORT

Product: N2L-EET-36 Gly-28-Gly
Sequence: Gly-Cys-Glu-Val-Val-Met-Glu-Arg-Cys-Lys-Gln-Asp-Ser-Asp-Cys-Leu-Ala-Gly-Cys-Val-Cys-Trp-Tyr-Trp-Thr-Ser-Cys-Gly

Note: Natural Oxidation

Product No.: GT0289 Expected M.W.: 3126.61 Found M.W.: 3215.90 Lot: U343

SUGGESTIONS FOR PEPTIDE DISSOLUTION: Acetonitrile / 0.1% TFA in Water

COUNTERIONS PRESENT: TFA Salt

STORAGE: All peptides should be stored dry at -20°C

This material is not listed as hazardous by ∗NIOSH/RTECS. Therefore, no SAFETY DATA SHEET is required. However, the chemical, physical and toxicological properties of this product have not been thoroughly investigated. Therefore, please exercise due care when handling this material. This action is in compliance with State and Federal OSHA standards and regulations.

Quality Control: ______________________

Date: January 24, 2019

CS Bio Co.
20 Kelly Court, Menlo Park, CA 94025 USA
T: (650) 322 1111 • F: (650) 322 2278
www.csbio.com • peptides@csbio.com

Compound: GT0289 N2L-EET-36 Gly-28-Gly Lot Number: U343 Expected M.W.: 3126.61 Found M.W.: 3125.90

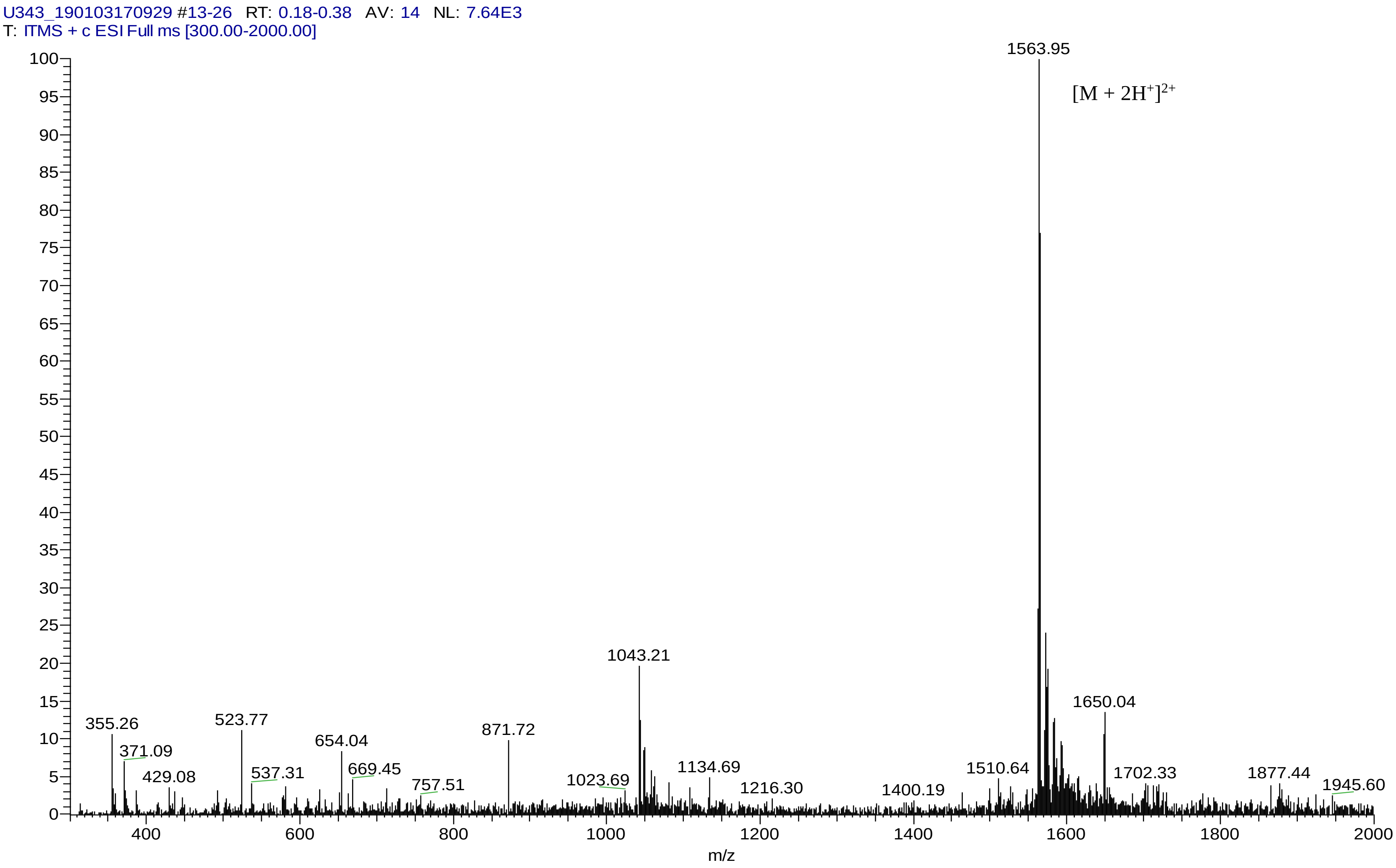

Sample Name: GT0289 (Before Lyophilization)
Lot#: U343
Instrument 1 Agilent 1260
Instrument ID: RD-HPLC 1
Injection Date: 1/2/2019
Inj. Volume: 20.0 uL

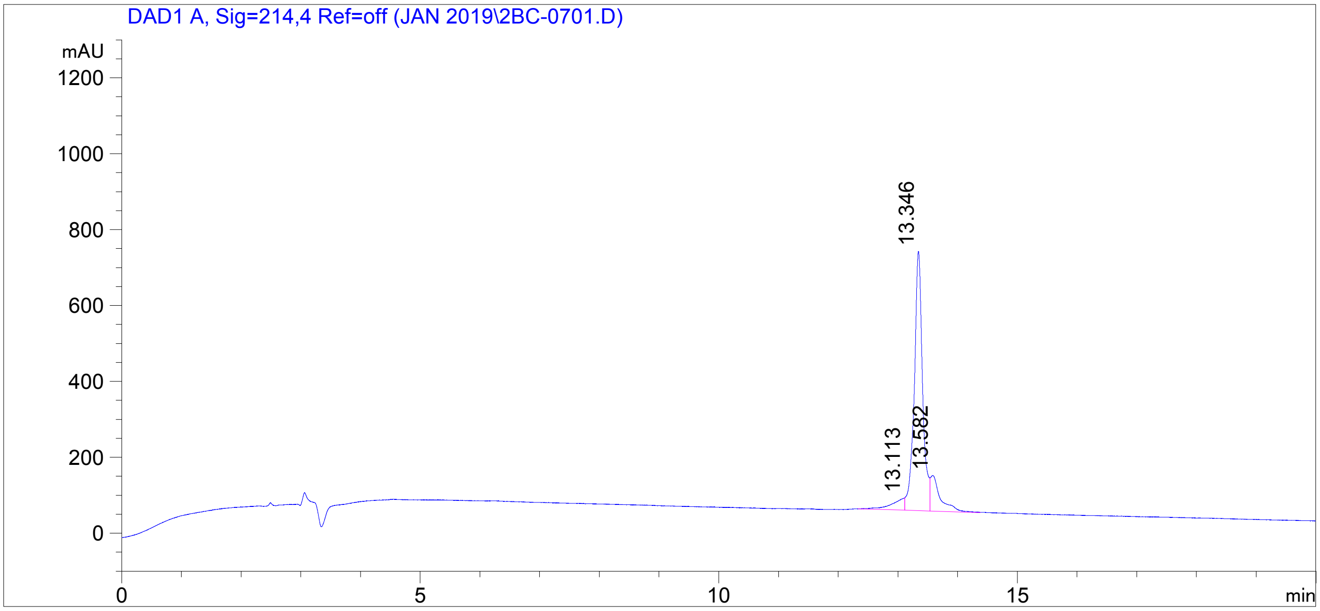

Data file name: C:\CHEM32\1\DATA\JAN 2019\2BC-0701.D

Acq. Method: C:\Chem32\1\DATA\Jan2019\PURIFICATION 2019-01-02 14-39-36\20-50-20-1-6.M
Column: Phenomenex Luna C18 (2) 5u 100A 250x4.6mm P/N: 00G-4252-E0
Buffer A: 0.1% TFA in H2O
Buffer B: 0.1% TFA in ACN
Flow Rate: 1ml/min
Gradient: 20-50% in 20 min
Temperature: 60C

Peak #	RT [min]	Area	Height	Area %
1	13.113	463.31	32.60	5.61
2	13.346	6661.63	683.54	80.70
3	13.582	1130.09	94.21	13.69

Sample Name: GT0289
Lot#: U343
Instrument 1 Agilent 1260
Instrument ID: RD-HPLC 1
Injection Date: 1/3/2019
Inj. Volume: 20.0 uL

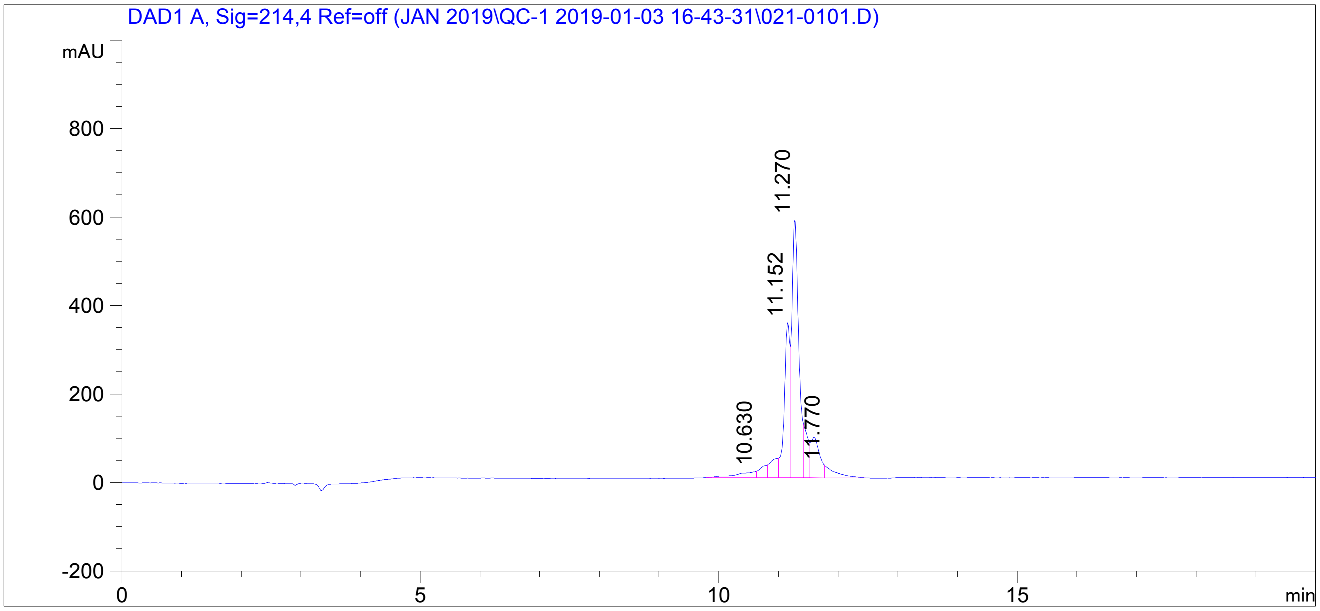

Data file name: C:\CHEM32\1\DATA\JAN 2019\QC-1 2019-01-03 16-43-31\021-0101.D

Acq. Method: C:\Chem32\1\DATA\JAN 2019\QC-1 2019-01-03 16-43-31\20-50-20.M

Column: Phenomenex Luna C18, 5um 250 x 4.6mm
Buffer A: 0.1% TFA in H2O
Buffer B: 0.1% TFA in ACN
Flow Rate: 1ml/min
Gradient: 20-50% B in 20 min

Peak #	RT [min]	Area	Height	Area %
1	10.630	314.71	14.52	3.18
2	10.817	256.90	29.03	2.60
3	10.997	431.87	44.22	4.37
4	11.152	2250.72	350.71	22.76
5	11.270	4704.32	583.30	47.58
6	11.416	638.74	121.50	6.46
7	11.595	947.36	91.82	9.58
8	11.770	342.63	28.25	3.47
