# Supplementary material for: Disulfide-constrained peptide scaffolds enable a robust peptide-therapeutic discovery platform
Source: PLoS One. 2024 Mar 28;19(3):e0300135. doi: 10.1371/journal.pone.0300135 (PMC10977697; doi:10.1371/journal.pone.0300135)
Supplement: S1 File — A zip file contains 51 pdf files with filenames are the same as the “DCP name” listed in the tables. (ZIP) [file pone.0300135.s004.zip › N2L-EET-57.pdf]

SAMPLE TEST REPORT

Product: N2L-EET-n7 Gly-32-Gly
Sequence: Gly-Cys-Asp-Gln-Gly-His-Ser-Ser-Gly-Trp-Gln-Lys-Cys-Lys-Gln-Asp-Ser-Asp-Cys-Leu-Ala-Gly-Cys-Val-Cys-Trp-Phe-Arg-Trp-His-Cys-Gly

Note: Natural Oxidation

Product No.: GT0281 Expected M.W.: 3583.00 Found M.W.: 3585.36 Lot: U117

APPEARANCE:	White Powder
MOLECULAR WEIGHT VERIFICATION:	Confirmed
PURITY: Instrument: Waters H Class System<br>Condition: HPLC column in TFA System<br>Gradient: 15-45% Buffer B in 20 minutes<br>Buffer A: 0.1% TFA in H_2O<br>Buffer B: 0.1% TFA in ACN<br>Wavelength: 214 nm<br>Column: Phenomenex Luna C18 5µm 100Å, 4.6 x 250 mm	96.30%
ELLMAN'S TEST:	Complies
PEPTIDE CONTENT:<br>(By Amino Acid Analysis)	80.7%
SUGGESTIONS FOR PEPTIDE DISSOLUTION:	Water
COUNTERIONS PRESENT:	TFA Salt
STORAGE:	All peptides should be stored dry at -20°C

This material is not listed as hazardous by ∗NIOSH/RTECS. Therefore, no SAFETY DATA SHEET is required. However, the chemical, physical and toxicological properties of this product have not been thoroughly investigated. Therefore, please exercise due care when handling this material. This action is in compliance with State and Federal OSHA standards and regulations.

Quality Control: ____________________

Date: October 25, 2018

CS Bio Co.
20 Kelly Court, Menlo Park, CA 94025 USA
T: (650) 322 1111 • F: (650) 322 2278
www.csbio.com • peptides@csbio.com

Compound: GT0281 N2L-EET-n7 Gly-32-Gly Lot Number: U117 Expected M.W.: 3583.00 Found M.W.: 3585.36

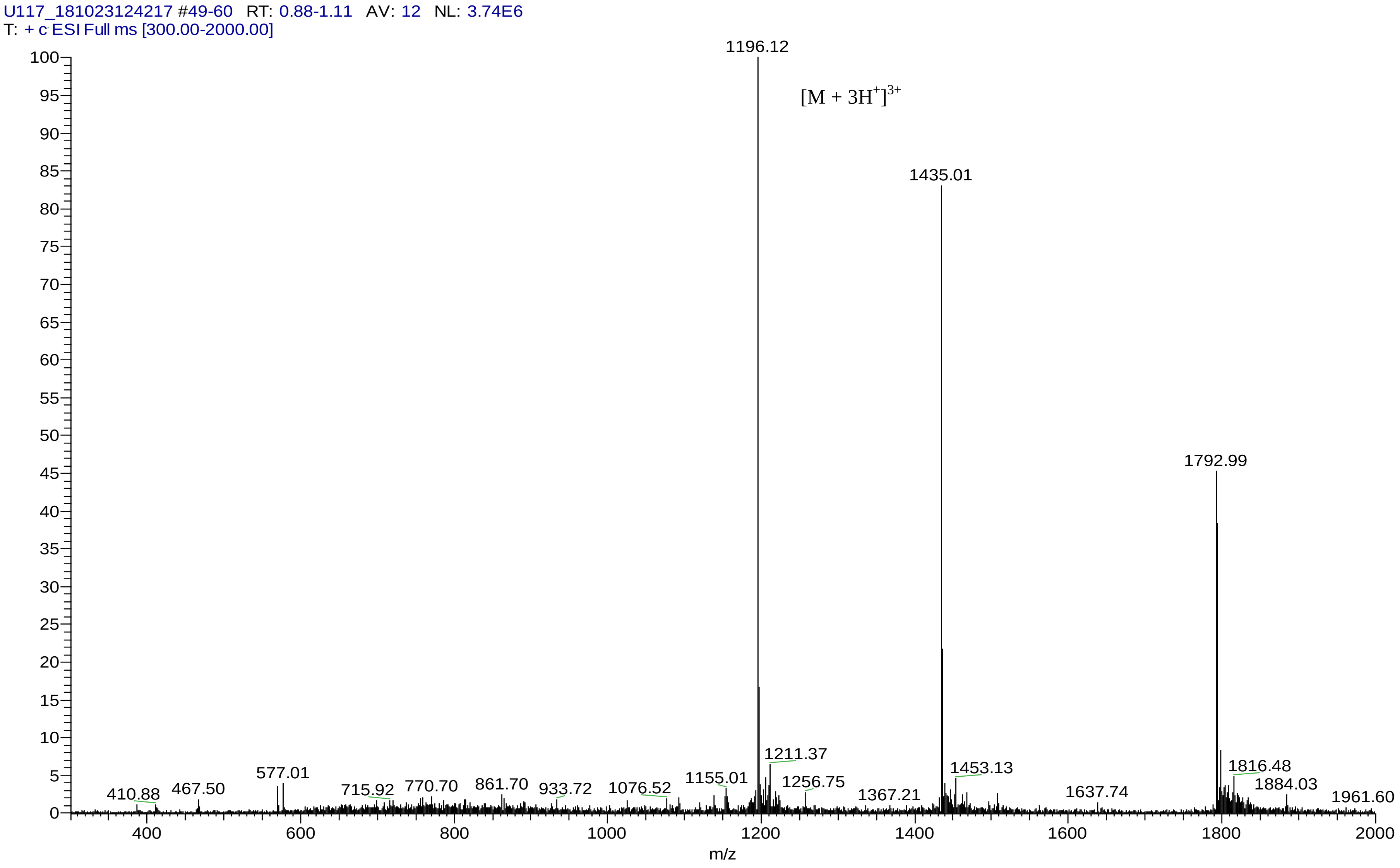

SAMPLE INFORMATION

Sample Name:	GT0281 U117	Acquired By:	RDQC
Sample Type:	Unknown	Sample Set Name	QC102218
Vial:	1:A,1	Acq. Method Set:	15_45_20_214nm
Injection #:	1	Processing Method	RD QC
Injection Volume:	20.00 ul	Channel Name:	PDA Ch1 214nm@4.8nm
Run Time:	20.0 Minutes		PDA Ch1 214nm@4.8nm
Column	Phenomenex, Luna, C18(2), 5u 100A 250 x 4.6mm		
Date Acquired:	10/23/2018 11:29:05 AM PDT		
Date Processed:	10/23/2018 12:26:46 PM PDT		
Buffer:	A: 0.1% TFA in Water; B: 0.1% TFA in Acetonitrile		
Flow Rate:	1.0mL/min		

Auto-Scaled Chromatogram

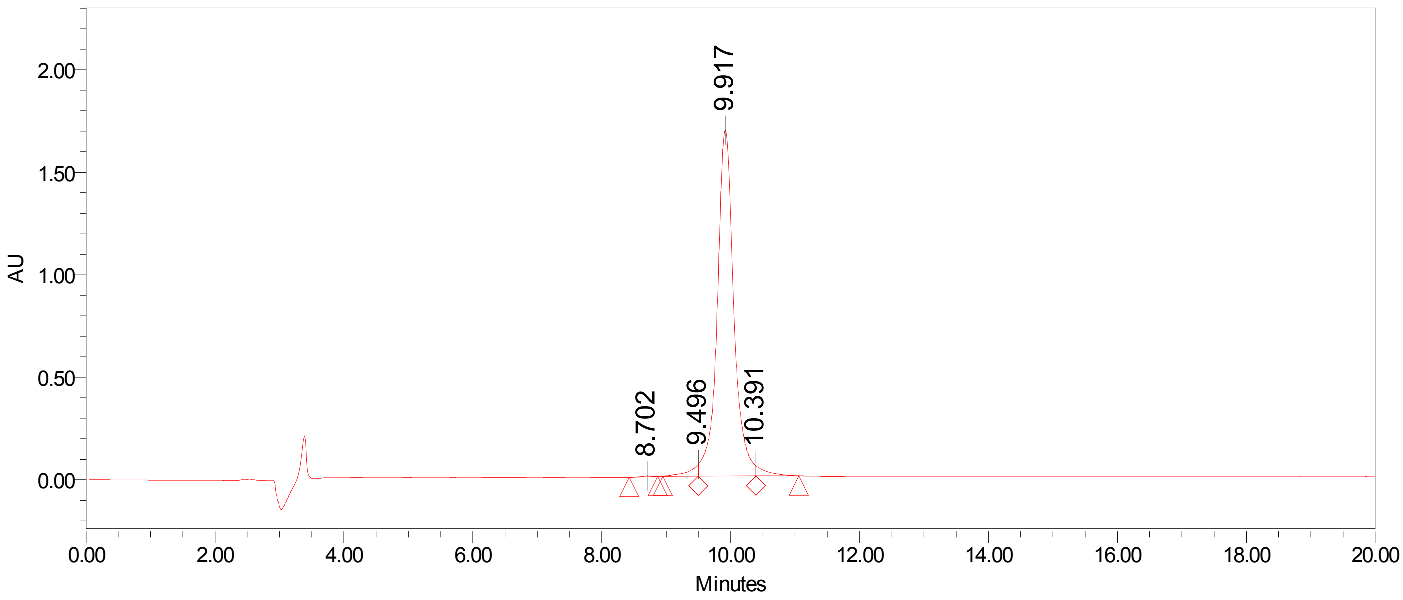

Peak Results

		8.702	39739	3951	0.13
		9.496	554558	56973	1.77
		9.917	30233474	1686214	96.30
		10.391	568334	49788	1.81

Peptide Content Determination by Amino Acid Analysis

Instrument Model: Waters H Class System
Sample Name: N2L-EET-n7 Gly-32-Gly
Sample ID: GT0281
Lot No.: U117
Sample Testing Date: 10/25/2018

**Peptide Content (%)**	**80.7%**

Performed by:

Shilpa Patel. 10/25/2018

Name Date

Reviewed by:

[illegible] 10/25/2018

Name Date
