# Supplementary material for: Disulfide-constrained peptide scaffolds enable a robust peptide-therapeutic discovery platform
Source: PLoS One. 2024 Mar 28;19(3):e0300135. doi: 10.1371/journal.pone.0300135 (PMC10977697; doi:10.1371/journal.pone.0300135)
Supplement: S1 File — A zip file contains 51 pdf files with filenames are the same as the “DCP name” listed in the tables. (ZIP) [file pone.0300135.s004.zip › N2L-EET-62.pdf]

SAMPLE TEST REPORT

Product: N2L-EET-62 Gly-30-Gly

Sequence: Gly-Cys-Gly-Gln-Thr-Thr-Ala-Trp-Glu-Pro-Cys-Lys-Gln-Asp-Ser-Asp-Cys-Leu-Ala-Gly-Cys-Val-Cys-Phe-Met-Arg-Trp-His-Cys-Gly

Note: Natural Oxidation

Product No.: GT0283   Expected M.W.: 3287.77   Found M.W.: 3287.88   Lot: U243

APPEARANCE:	White Powder
MOLECULAR WEIGHT VERIFICATION:	Confirmed
PURITY: Instrument: Waters H Class System<br>Condition: HPLC column in TFA System<br>Gradient: 20-50% Buffer B in 20 minutes<br>Buffer A: 0.1% TFA in H_2O<br>Buffer B: 0.1% TFA in ACN<br>Wavelength: 214 nm<br>Column: Phenomenex Luna C18 5μm 100Å, 4.6 x 250 mm	95.26%
ELLMAN'S TEST:	Complies
SUGGESTIONS FOR PEPTIDE DISSOLUTION:	Water
COUNTERIONS PRESENT:	TFA Salt
STORAGE:	All peptides should be stored dry at -20°C

This material is not listed as hazardous by ∗NIOSH/RTECS. Therefore, no SAFETY DATA SHEET is required. However, the chemical, physical and toxicological properties of this product have not been thoroughly investigated. Therefore, please exercise due care when handling this material. This action is in compliance with State and Federal OSHA standards and regulations.

Quality Control: ______________

Date: December 13, 2018

CS Bio Co.
20 Kelly Court, Menlo Park, CA 94025 USA
T: (650) 322 1111 • F: (650) 322 2278
www.csbio.com • peptides@csbio.com

Compound: GT0283 N2L-EET-62 Gly-30-Gly Lot Number: U243 Expected M.W.: 3287.77 Found M.W.: 3287.88

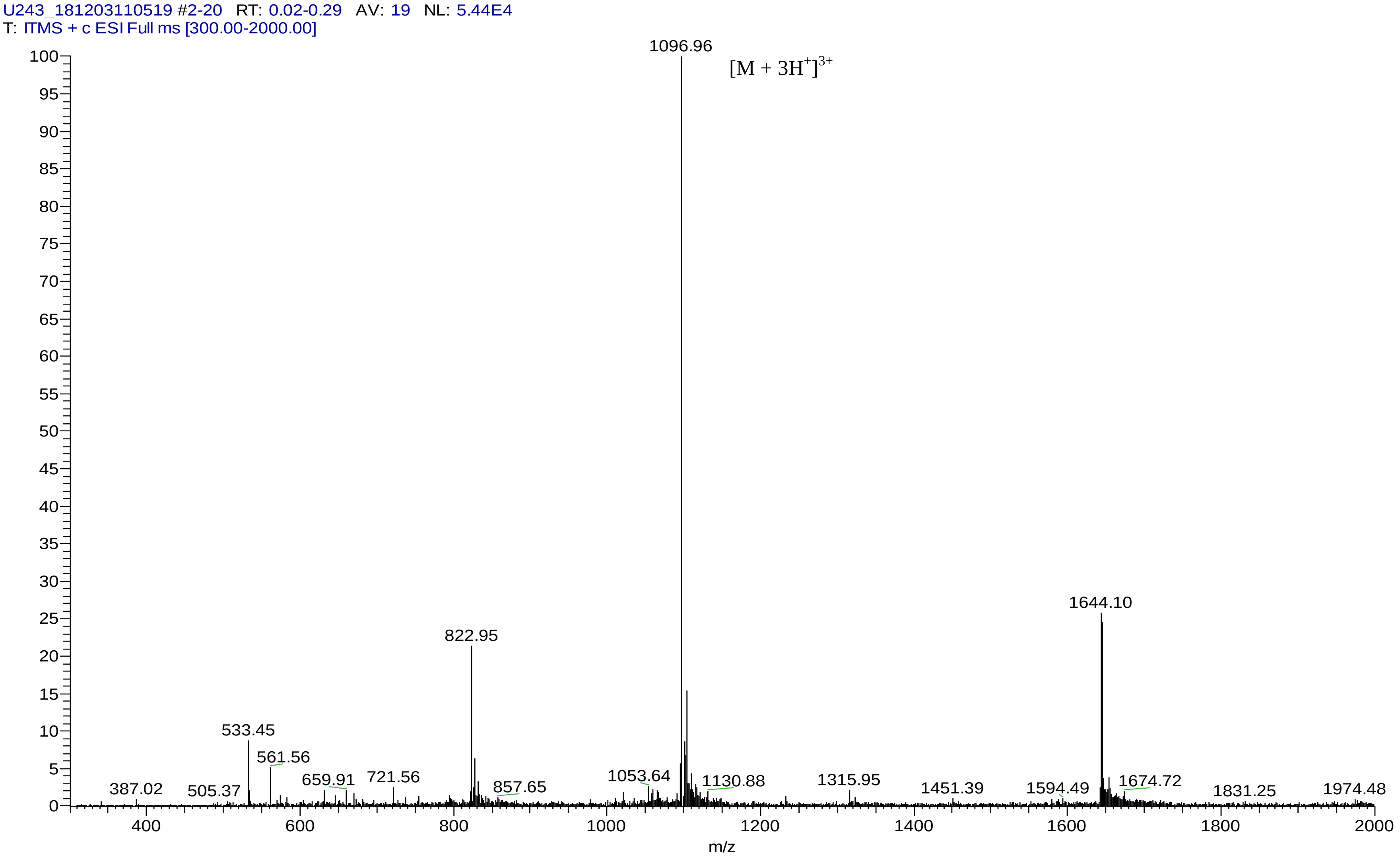

SAMPLE INFORMATION

Sample Name:	GT0283 U243	Acquired By:	RDQC
Sample Type:	Unknown	Sample Set Name	QC120318
Vial:	1:A,1	Acq. Method Set:	20_50_20_214nm
Injection #:	1	Processing Method	RD QC
Injection Volume:	40.00 ul	Channel Name:	PDA Ch1 214nm@4.8nm
Run Time:	20.0 Minutes		PDA Ch1 214nm@4.8nm
Column	Phenomenex, Luna, C18(2), 5u 100A 250 x 4.6mm		
Date Acquired:	12/3/2018 9:35:16 AM PST		
Date Processed:	12/3/2018 10:03:14 AM PST		
Buffer:	A: 0.1% TFA in Water; B: 0.1% TFA in Acetonitrile		
Flow Rate:	1.0mL/min		

Auto-Scaled Chromatogram

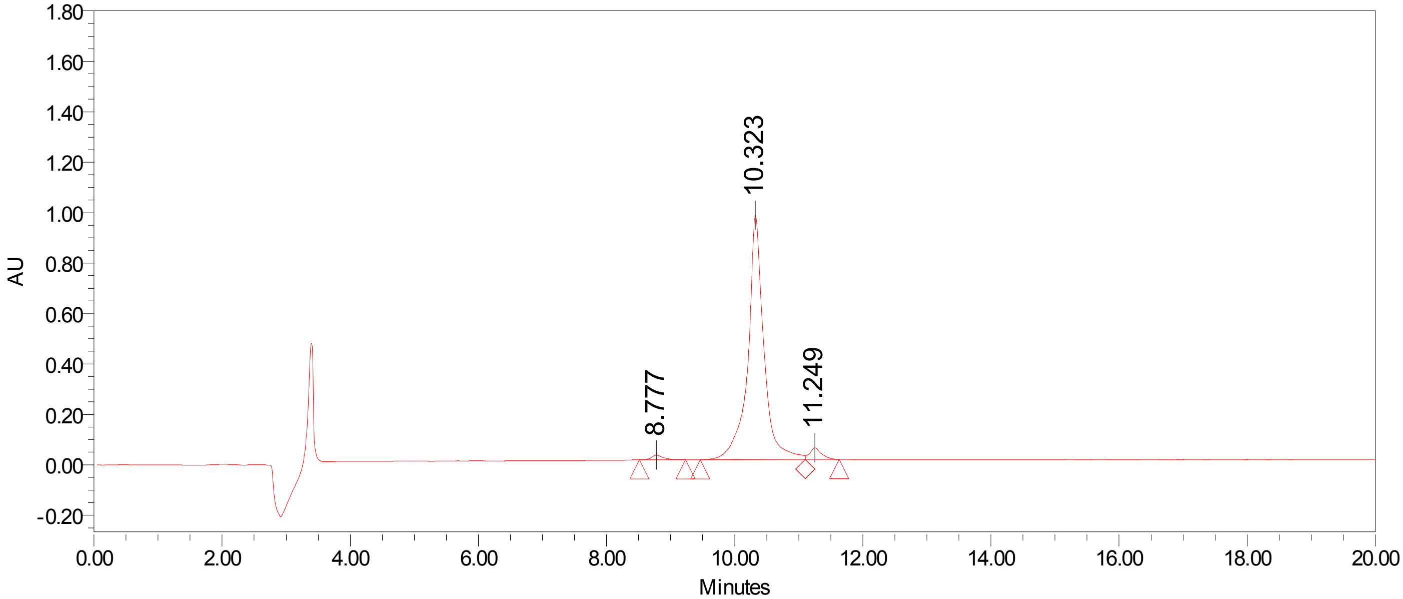

Peak Results

[redacted]	[redacted]	[redacted]	[redacted]	[redacted]	[redacted]
[redacted]		8.777	237760	18565	1.34
[redacted]		10.323	16893776	968676	95.26
[redacted]		11.249	602962	47048	3.40
