# Supplementary material for: Disulfide-constrained peptide scaffolds enable a robust peptide-therapeutic discovery platform
Source: PLoS One. 2024 Mar 28;19(3):e0300135. doi: 10.1371/journal.pone.0300135 (PMC10977697; doi:10.1371/journal.pone.0300135)
Supplement: S1 File — A zip file contains 51 pdf files with filenames are the same as the “DCP name” listed in the tables. (ZIP) [file pone.0300135.s004.zip › N2L-EET-63.pdf]

SAMPLE TEST REPORT

Product: N2L-EET-63 Gly-30-Gly
Sequence: Gly-Cys-Glu-Gln-Ser-Thr-Trp-Trp-Ala-Pro-Cys-Lys-Gln-Asp-Ser-Asp-Cys-Leu-Ala-Gly-Cys-Val-Cys-Tyr-Gln-Arg-Trp-His-Cys-Gly

Note: Natural Oxidation

Product No.: GT0261 Expected M.W.: 3415.82 Found M.W.: 3415.71 Lot: T984

APPEARANCE:	White Powder
MOLECULAR WEIGHT VERIFICATION:	Confirmed
PURITY: Instrument: Agilent 1260 System<br>Condition: HPLC column in TFA System<br>Gradient: 20-50% Buffer B in 20 minutes<br>Buffer A: 0.1% TFA in H_2O<br>Buffer B: 0.1% TFA in ACN<br>Wavelength: 214 nm<br>Column: Phenomenex Luna C18 5µm 100Å, 4.6 x 250 mm	95.70%
ELLMAN'S TEST:	Complies
SUGGESTIONS FOR PEPTIDE DISSOLUTION:	Acetonitrile / 0.1% TFA in Water
COUNTERIONS PRESENT:	TFA Salt
STORAGE:	All peptides should be stored dry at -20°C

This material is not listed as hazardous by ∗NIOSH/RTECS. Therefore, no SAFETY DATA SHEET is required. However, the chemical, physical and toxicological properties of this product have not been thoroughly investigated. Therefore, please exercise due care when handling this material. This action is in compliance with State and Federal OSHA standards and regulations.

Quality Control: Xiaoshy Jin

Date: September 2, 2018

CS Bio Co.
20 Kelly Court, Menlo Park, CA 94025 USA
T: (650) 322 1111 • F: (650) 322 2278
www.csbio.com • peptides@csbio.com

Compound: GT0261 N2L-EET-63 Gly-30-Gly Lot Number: T984 Expected M.W.: 3415.82 Found M.W.: 3415.71

T984_180831171913 #41-55 RT: 0.68-0.92 AV: 15 NL: 1.92E7
T: + c ESI Full ms [300.00-2000.00]

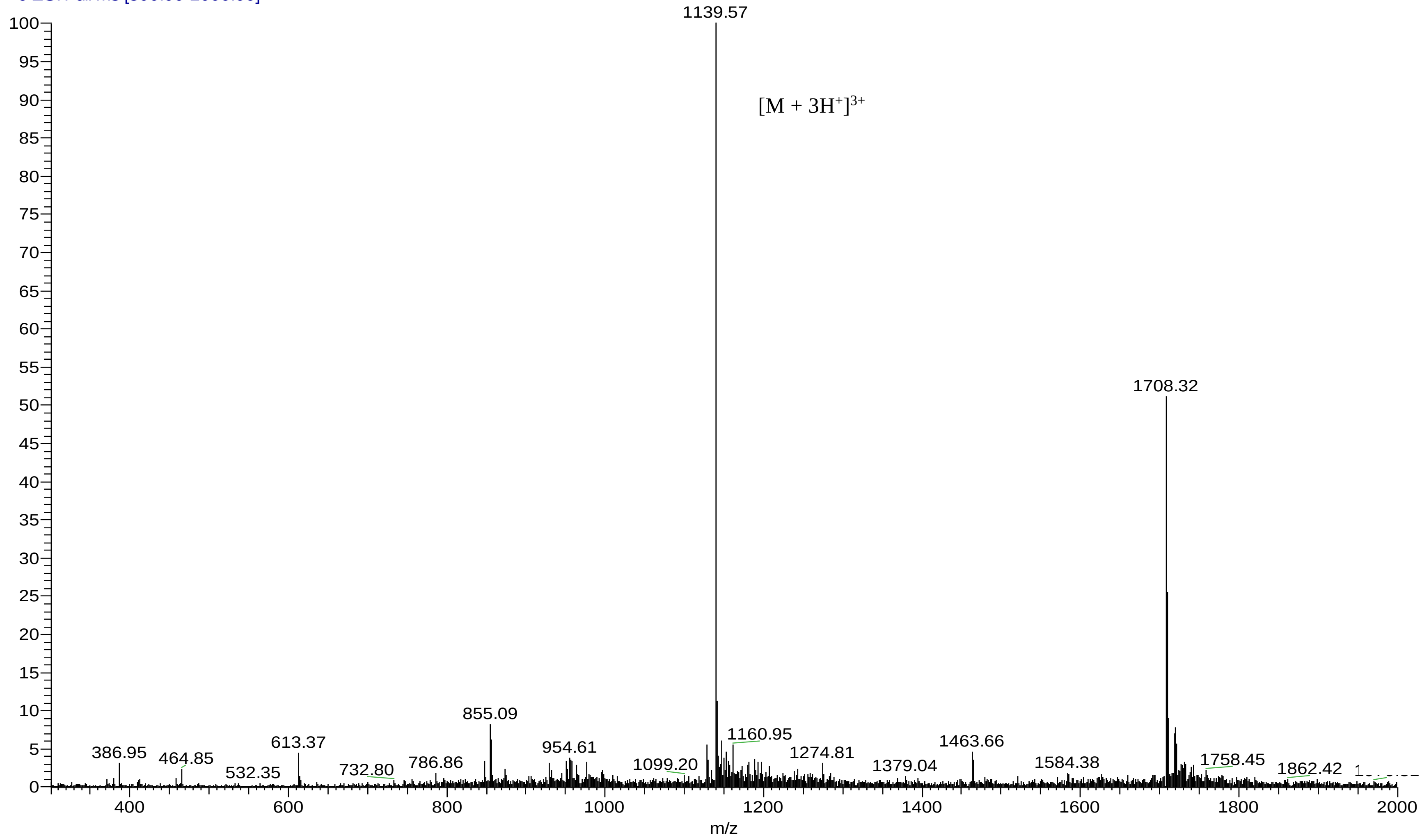

Sample Name: GT0261
Lot#: T984
Instrument 1 Agilent 1260
Instrument ID: RD-HPLC 1
Injection Date: 9/2/2018
Inj. Volume: 100.0uL

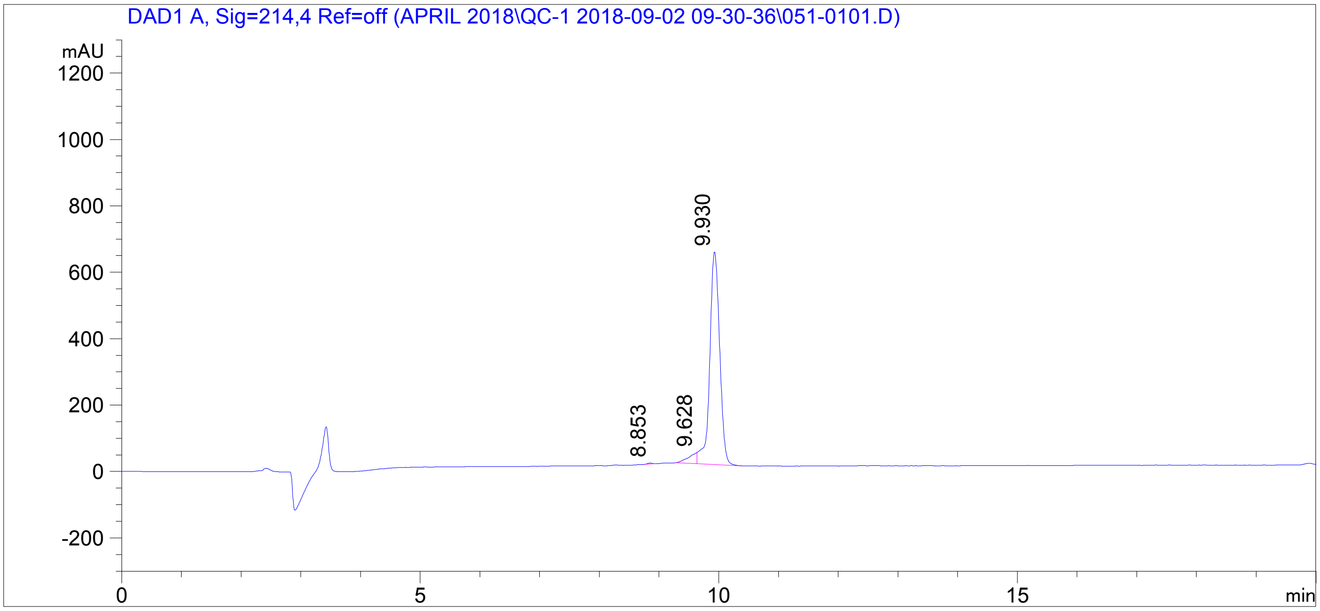

Data file name: C:\CHEM32\1\DATA\APRIL 2018\QC-1 2018-09-02 09-30-36\051-0101.D

Acq. Method: C:\Chem32\1\DATA\April 2018\QC-1 2018-09-02 09-30-36\20-50-20.M

Column: Phenomenex Luna C18, 5um 250 x 4.6mm
Buffer A: 0.1% TFA in H2O
Buffer B: 0.1% TFA in ACN
Flow Rate: 1ml/min
Gradient: 20-50% B in 20 min

Peak #	RT [min]	Area	Height	Area %
1	8.853	17.68	2.95	0.23
2	9.628	316.64	32.85	4.07
3	9.930	7438.86	640.49	95.70
