# Supplementary material for: Disulfide-constrained peptide scaffolds enable a robust peptide-therapeutic discovery platform
Source: PLoS One. 2024 Mar 28;19(3):e0300135. doi: 10.1371/journal.pone.0300135 (PMC10977697; doi:10.1371/journal.pone.0300135)
Supplement: S1 File — A zip file contains 51 pdf files with filenames are the same as the “DCP name” listed in the tables. (ZIP) [file pone.0300135.s004.zip › N2L-EET-83.pdf]

SAMPLE TEST REPORT

Product: N2L-EET-83 Gly-30-Gly
Sequence: Gly-Cys-Glu-Gln-Ser-Thr-Trp-Trp-Ala-Pro-Cys-Lys-Gln-Asp-Ser-Asp-Cys-Leu-Ala-Gly-Cys-Val-Cys-Tyr-Leu-Arg-Trp-His-Cys-Gly

Note: Natural Oxidation

Product No.: GT0282 Expected M.W.: 3400.86 Found M.W.: 3401.58 Lot: U157

APPEARANCE:	White Powder
MOLECULAR WEIGHT VERIFICATION:	Confirmed
PURITY: Instrument: Agilent 1260 System<br>Condition: HPLC column in TFA System<br>Gradient: 25-55% Buffer B in 20 minutes<br>Buffer A: 0.1% TFA in H_2O<br>Buffer B: 0.1% TFA in ACN<br>Wavelength: 214 nm<br>Column: Phenomenex Luna C18 5µm 100Å, 4.6 x 250 mm	86.25% (Before Lyophilization)
PURITY: Instrument: Waters H Class System<br>Condition: HPLC column in TFA System<br>Gradient: 25-55% Buffer B in 20 minutes<br>Buffer A: 0.1% TFA in H2O<br>Buffer B: 0.1% TFA in ACN<br>Wavelength: 214 nm<br>Column: Phenomenex Luna C18 5µm 100Å, 4.6 x 250 mm	78.61% (After Lyophilization)
ELLMAN'S TEST:	Complies

CS Bio Co.
20 Kelly Court, Menlo Park, CA 94025 USA
T: (650) 322 1111 • F: (650) 322 2278
www.csbio.com • peptides@csbio.com

SAMPLE TEST REPORT

Product: N2L-EET-83 Gly-30-Gly
Sequence: Gly-Cys-Glu-Gln-Ser-Thr-Trp-Trp-Ala-Pro-Cys-Lys-Gln-Asp-Ser-Asp-Cys-Leu-Ala-Gly-Cys-Val-Cys-Tyr-Leu-Arg-Trp-His-Cys-Gly

Note: Natural Oxidation

Product No.: GT0282 Expected M.W.: 3400.86 Found M.W.: 3401.58 Lot: U157

PEPTIDE CONTENT: 83.2%
(By Amino Acid Analysis)

SUGGESTIONS FOR PEPTIDE DISSOLUTION: Water

COUNTERIONS PRESENT: TFA Salt

STORAGE: All peptides should be stored dry at -20°C

This material is not listed as hazardous by ∗NIOSH/RTECS. Therefore, no SAFETY DATA SHEET is required. However, the chemical, physical and toxicological properties of this product have not been thoroughly investigated. Therefore, please exercise due care when handling this material. This action is in compliance with State and Federal OSHA standards and regulations.

Quality Control: ____________________

Date: November 8, 2018

CS Bio Co.
20 Kelly Court, Menlo Park, CA 94025 USA
T: (650) 322 1111 • F: (650) 322 2278
www.csbio.com • peptides@csbio.com

Compound: GT0282 N2L-EET-83 Gly-30-Gly Lot Number: U157 Expected M.W.: 3400.86 Found M.W.: 3401.58

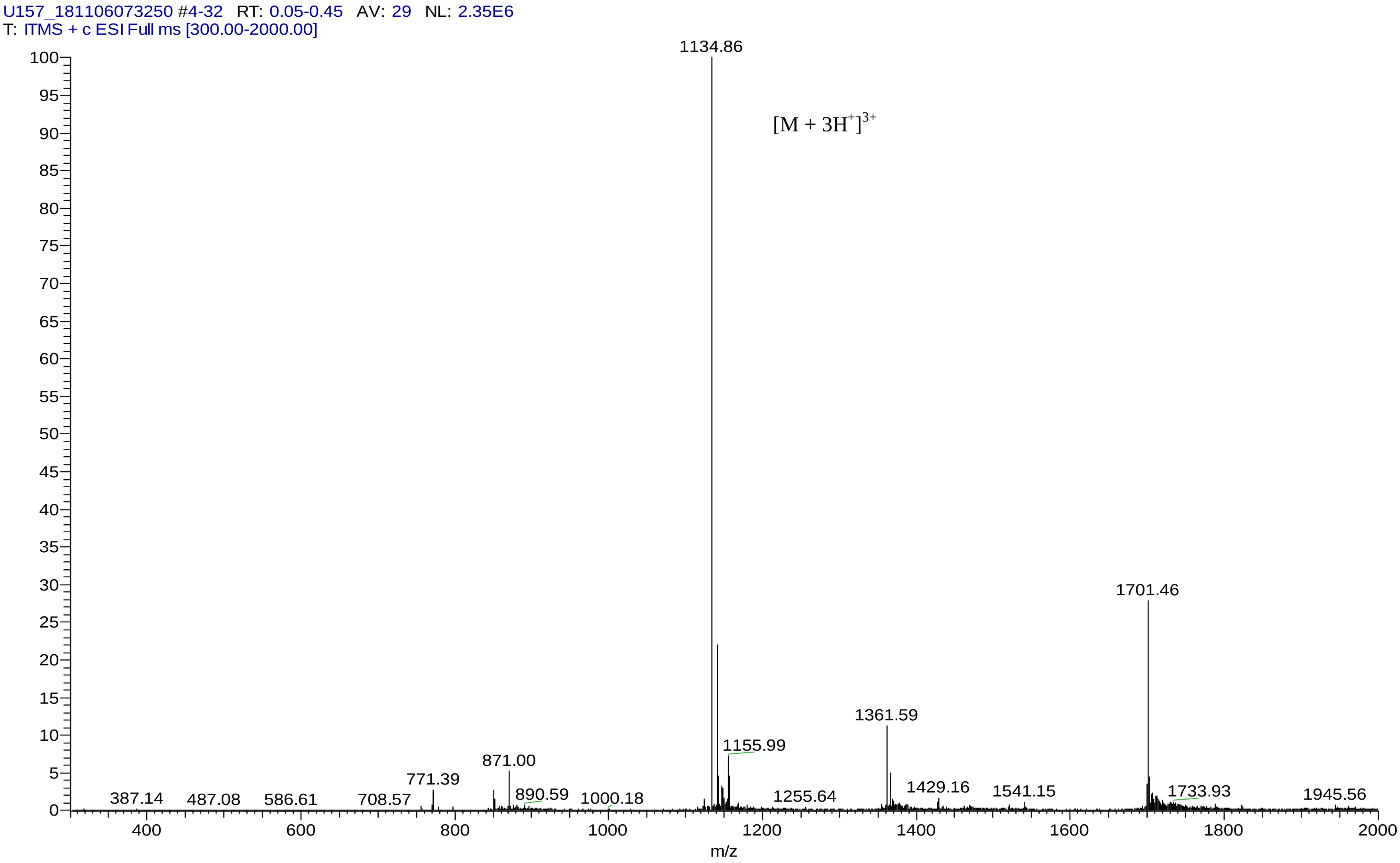

Sample Name: GT0282 (Before Lyophilization)
Lot#: U157
Instrument 1 Agilent 1260
Instrument ID: RD-HPLC 1
Injection Date: 11/2/2018
Inj. Volume: 20.0 uL

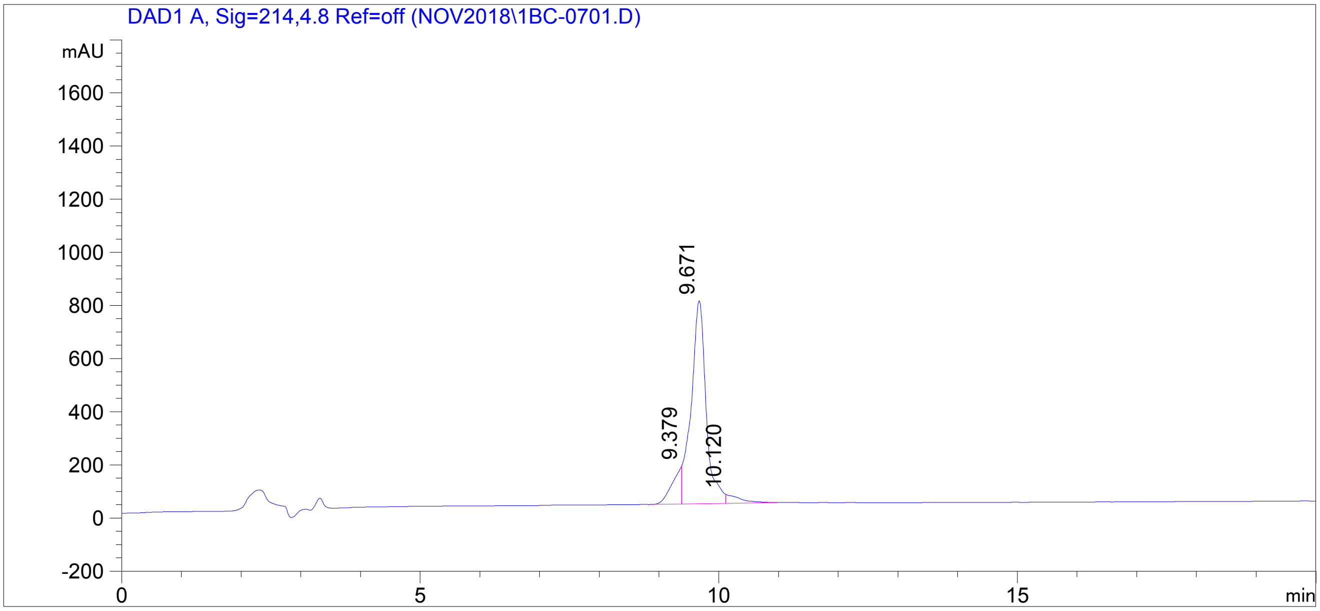

Data file name: C:\CHEM32\1\DATA\NOV2018\1BC-0701.D

Acq. Method: C:\Chem32\1\DATA\Nov2018\PURIFICATION 2018-11-02 14-16-03\25-55-20-1-6.M
Column: Phenomenex Luna C18 (2) 5u 100A 250x4.6mm P/N: 00G-4252-E0
Buffer A: 0.1% TFA in H2O
Buffer B: 0.1% TFA in ACN
Flow Rate: 1ml/min
Gradient: 25-55% B in 20 min

Peak #	RT [min]	Area	Height	Area %
1	9.379	1586.18	144.30	10.31
2	9.671	13262.73	764.37	86.25
3	10.120	528.67	33.03	3.44

Empower® 3 SOFTWARE

QC_1

SAMPLE INFORMATION

Sample Name:	GT0282 U157	Acquired By:	RDQC
Sample Type:	Unknown	Sample Set Name	QC110518
Vial:	1:A.1	Acq. Method Set:	25_55_20_214nm
Injection #:	1	Processing Method	RD QC
Injection Volume:	30.00 ul	Channel Name:	PDA Ch1 214nm@4.8nm
Run Time:	20.0 Minutes		PDA Ch1 214nm@4.8nm
Column	Phenomenex, Luna, C18(2), 5u 100A 250 x 4.6mm		
Date Acquired:	11/5/2018 3:24:32 PM PST		
Date Processed:	11/8/2018 12:10:52 PM PST		
Buffer:	A: 0.1% TFA in Water; B: 0.1% TFA in Acetonitrile		
Flow Rate:	1.0mL/min		

Auto-Scaled Chromatogram

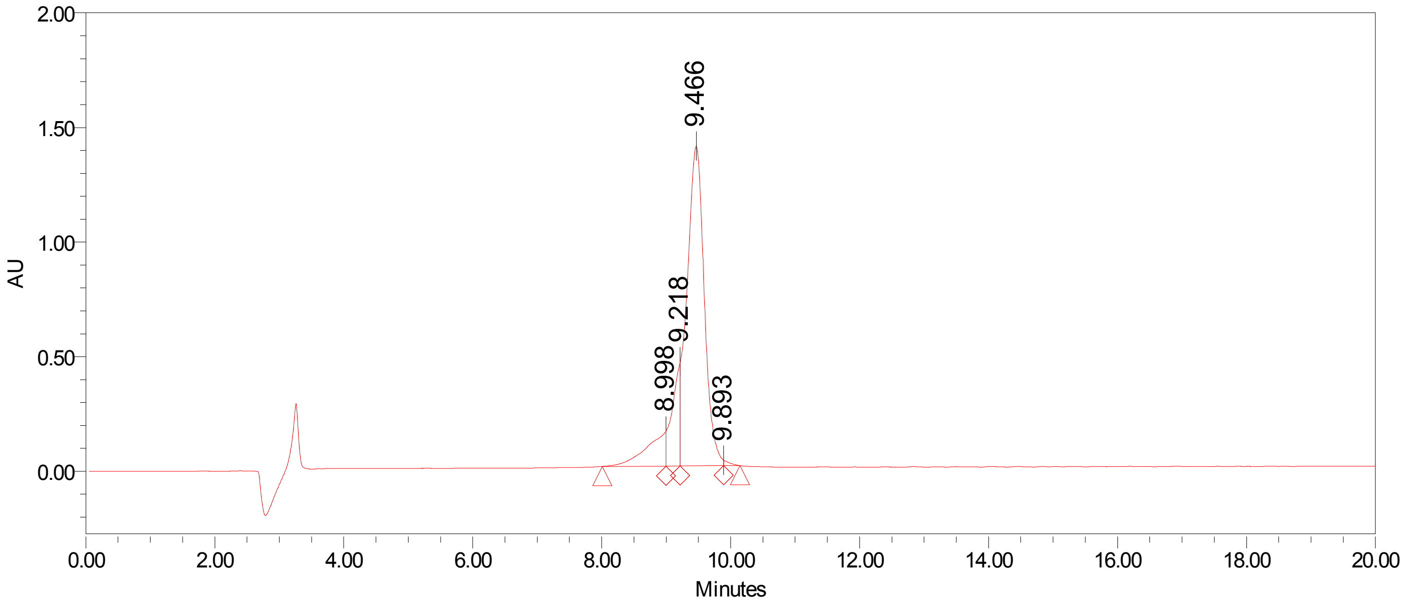

Peak Results

[redacted]					
		8.998	3153024	153369	9.54
		9.218	3776145	454956	11.42
		9.466	25994464	1395839	78.61
		9.893	143018	23772	0.43

Peptide Content Determination by Amino Acid Analysis

Instrument Model: Waters H Class System
Sample Name: N2L-EET-83 Gly-30-Gly
Sample ID: GT0282
Lot No.: U157
Sample Testing Date: 11/08/2018

**Peptide Content (%)**	**83.2%**

Performed by:

Shilpa Patel. 11/08/2018

Name Date

Reviewed by:

11/08/2018

Name Date
