# Supplementary material for: Disulfide-constrained peptide scaffolds enable a robust peptide-therapeutic discovery platform
Source: PLoS One. 2024 Mar 28;19(3):e0300135. doi: 10.1371/journal.pone.0300135 (PMC10977697; doi:10.1371/journal.pone.0300135)
Supplement: S1 File — A zip file contains 51 pdf files with filenames are the same as the “DCP name” listed in the tables. (ZIP) [file pone.0300135.s004.zip › N2L.EET03.58.pdf]

SAMPLE TEST REPORT

Product: N2L.EET03.L5.58 Gly-28-Gly
Sequence: Gly-Cys-Gly-Trp-Thr-Leu-Trp-His-Cys-Lys-Gln-Asp-Ser-Asp-Cys-Leu-Ala-Gly-Cys-Val-Cys-Glu-Pro-Lys-Trp-Phe-Cys-Gly

Note: Natural Oxidation

Product No.: GT0399 Expected M.W.: 3127.62 Found M.W.: 3127.58 Lot: U747

APPEARANCE: White Powder

MOLECULAR WEIGHT VERIFICATION: Confirmed

PURITY: Instrument: Agilent 1260 System
Condition: HPLC column in TFA System
Gradient: 20-50% Buffer B in 20 minutes
Buffer A: 0.1% TFA in H_2O
Buffer B: 0.1% TFA in ACN
Wavelength: 214 nm
Column: Phenomenex Luna C18 5µm 100Å, 4.6 x 250 mm

97.00% (Before Lyophilization)
90.33% (After Lyophilization)

PEPTIDE CONTENT: 77.1%
(By N Elemental Analysis)

ELLMAN'S TEST: Complies

SUGGESTIONS FOR PEPTIDE DISSOLUTION: Water

COUNTERIONS PRESENT: TFA Salt

STORAGE: All peptides should be stored dry at -20°C

This material is not listed as hazardous by ∗NIOSH/RTECS. Therefore, no SAFETY DATA SHEET is required. However, the chemical, physical and toxicological properties of this product have not been thoroughly investigated. Therefore, please exercise due care when handling this material. This action is in compliance with State and Federal OSHA standards and regulations.

Quality Control: ____________________

Date: May 24, 2019

CSBio
20 Kelly Court, Menlo Park, CA 94025 USA
T: (650) 322 1111 • F: (650) 322 2278
www.csbio.com • peptides@csbio.com

Compound: GT0399 N2L.EET03.L5.58 Gly-28-Gly Lot Number: U747 Expected M.W.: 3127.62 Found M.W.: 3127.58

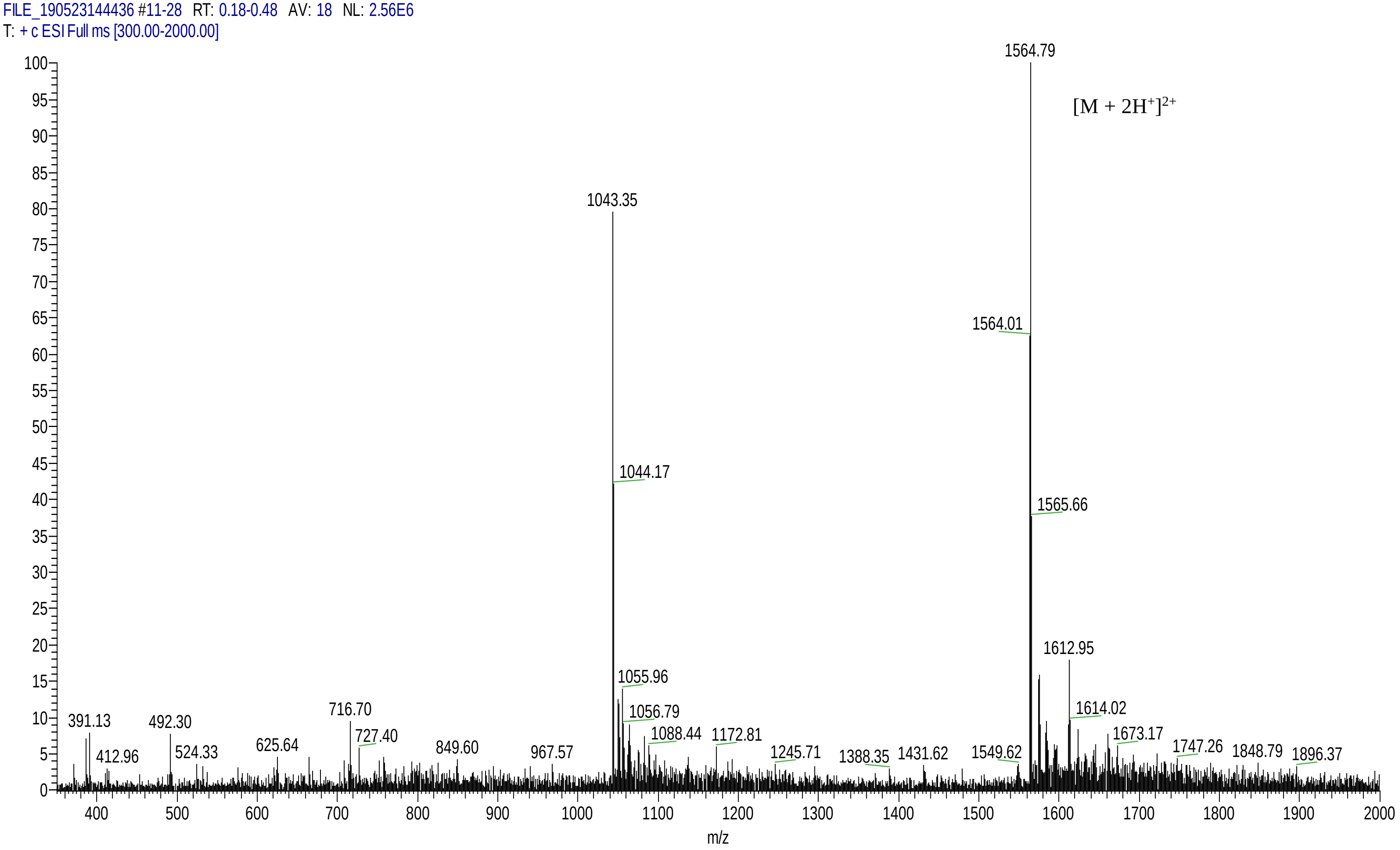

Sample Name: GT0399 (Before Lyophilization)
Lot#: U747
Instrument 1 Agilent 1260
Instrument ID: RD-HPLC 1
Injection Date: 5/21/2019
Inj. Volume: 8.0 uL

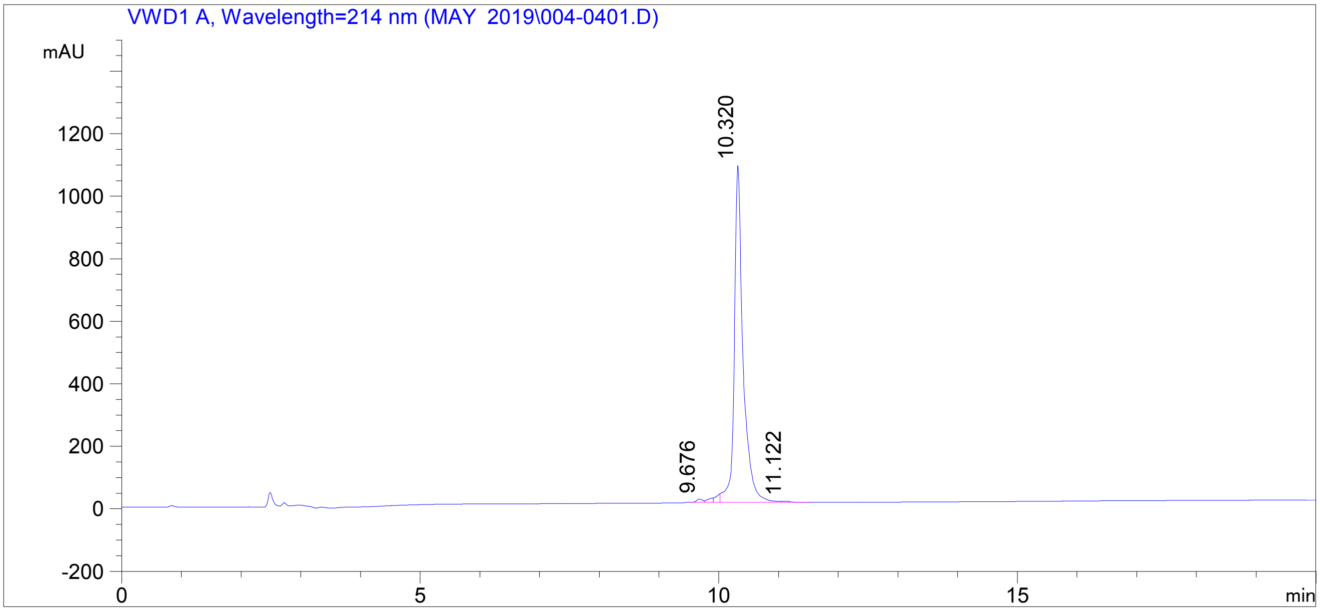

Data file name: C:\CHEM32\1\DATA\MAY 2019\004-0401.D

Acq. Method: C:\Chem32\1\DATA\MAY2019\WG20192019-05-2108-06-17\20-50-20.M

Column: Phenomenex Luna C18, 5um 250 x 4.6mm
Buffer A: 0.1% TFA in H2O
Buffer B: 0.1% TFA in ACN
Flow Rate: 1ml/min
Gradient: 20-50% B in 20 min

Peak #	RT [min]	Area	Height	Area %
1	9.676	75.18	10.48	0.66
2	9.906	89.89	14.25	0.79
3	10.022	142.51	27.99	1.25
4	10.320	11016.49	1077.81	97.00
5	11.122	33.41	3.63	0.29

Sample Name: GT0399
Lot#: U747
Instrument 1 Agilent 1260
Instrument ID: RD-HPLC 1
Injection Date: 5/22/2019
Inj. Volume: 10.0 uL

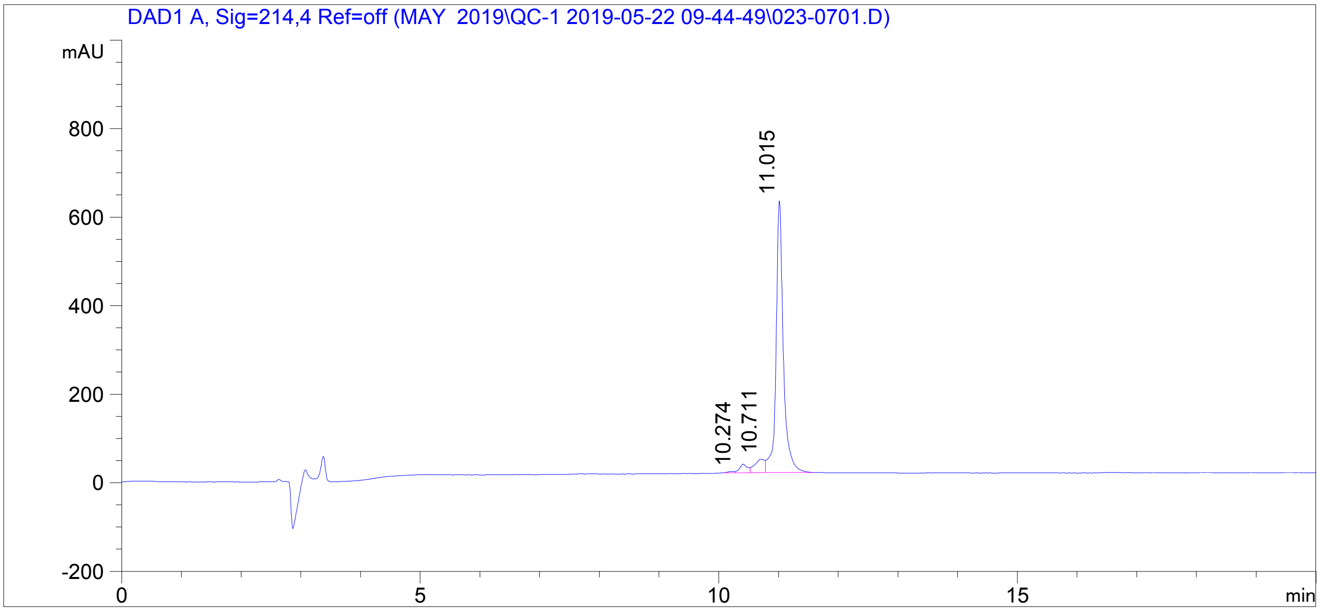

Data file name: C:\CHEM32\1\DATA\MAY 2019\QC-1 2019-05-22 09-44-49\023-0701.D

Acq. Method: C:\Chem32\1\DATA\May 2019\QC-1 2019-05-22 09-44-49\20-50-20.M

Column: Phenomenex Luna C18, 5um 250 x 4.6mm
Buffer A: 0.1% TFA in H2O
Buffer B: 0.1% TFA in ACN
Flow Rate: 1ml/min
Gradient: 20-50% B in 20 min

Peak #	RT [min]	Area	Height	Area %
1	10.274	27.19	3.13	0.47
2	10.409	179.27	19.28	3.10
3	10.711	353.12	30.41	6.10
4	11.015	5226.18	614.69	90.33

Peptide Content with Elemental Analysis

Analysis: Determination of Peptide Content by Nitrogen Content
Instrument Model: CE-440 Elemental Analyzer
Sample Name: N2L.EET03.L5.58 Gly-28-Gly
Sample ID: GT0399
Lot Number: U747
Sample Testing Date: 5/22/2019

	N%
**Expected Content**	16.12
**Actual Content**	12.43
**Peptide Content (%)**	**77.1**

Performed by:

5/22/2019

Name Date

Reviewed by:

5/22/2019

Name Date

CSBio
20 Kelly Court, Menlo Park, CA 94025 USA
Tel: 650-322-1111 Fax: 650-322-2278 Web: www.csbio.com Email: peptides@csbio.com
