# Supplementary material for: Disulfide-constrained peptide scaffolds enable a robust peptide-therapeutic discovery platform
Source: PLoS One. 2024 Mar 28;19(3):e0300135. doi: 10.1371/journal.pone.0300135 (PMC10977697; doi:10.1371/journal.pone.0300135)
Supplement: S1 File — A zip file contains 51 pdf files with filenames are the same as the “DCP name” listed in the tables. (ZIP) [file pone.0300135.s004.zip › N2L.EET03.63.pdf]

SAMPLE TEST REPORT

Product: N2L.EET03.L15.63 Gly-28-Gly
Sequence: Gly-Cys-Gly-Trp-Thr-Leu-Arg-His-Cys-Lys-Gln-Asp-Ser-Asp-Cys-Leu-Ala-Gly-Cys-Val-Cys-Glu-Pro-Lys-Trp-Trp-Cys-Gly

Note: Natural Oxidation

Product No.: GT0396 Expected M.W.: 3136.63 Found M.W.: 3136.92 Lot: U737

APPEARANCE:	White Powder
MOLECULAR WEIGHT VERIFICATION:	Confirmed
PURITY: Instrument: Agilent 1260 System<br>Condition: HPLC column in TFA System<br>Gradient: 20-50% Buffer B in 20 minutes<br>Buffer A: 0.1% TFA in H_2O<br>Buffer B: 0.1% TFA in ACN<br>Wavelength: 214 nm<br>Column: Phenomenex Luna C18 5µm 100Å, 4.6 x 250 mm	95.30%
PEPTIDE CONTENT:<br>(By N Elemental Analysis)	79.0%
ELLMAN'S TEST:	Complies
SUGGESTIONS FOR PEPTIDE DISSOLUTION:	Water
COUNTERIONS PRESENT:	TFA Salt
STORAGE:	All peptides should be stored dry at -20°C

This material is not listed as hazardous by ∗NIOSH/RTECS. Therefore, no SAFETY DATA SHEET is required. However, the chemical, physical and toxicological properties of this product have not been thoroughly investigated. Therefore, please exercise due care when handling this material. This action is in compliance with State and Federal OSHA standards and regulations.

Quality Control: ____________________

Date: May 20, 2019

CSBio
20 Kelly Court, Menlo Park, CA 94025 USA
T: (650) 322 1111 • F: (650) 322 2278
www.csbio.com • peptides@csbio.com

Compound: GT0396 N2L.EET03.L15.63 Gly-28-Gly Lot Number: U737 Expected M.W.: 3136.63 Found M.W.: 3136.92

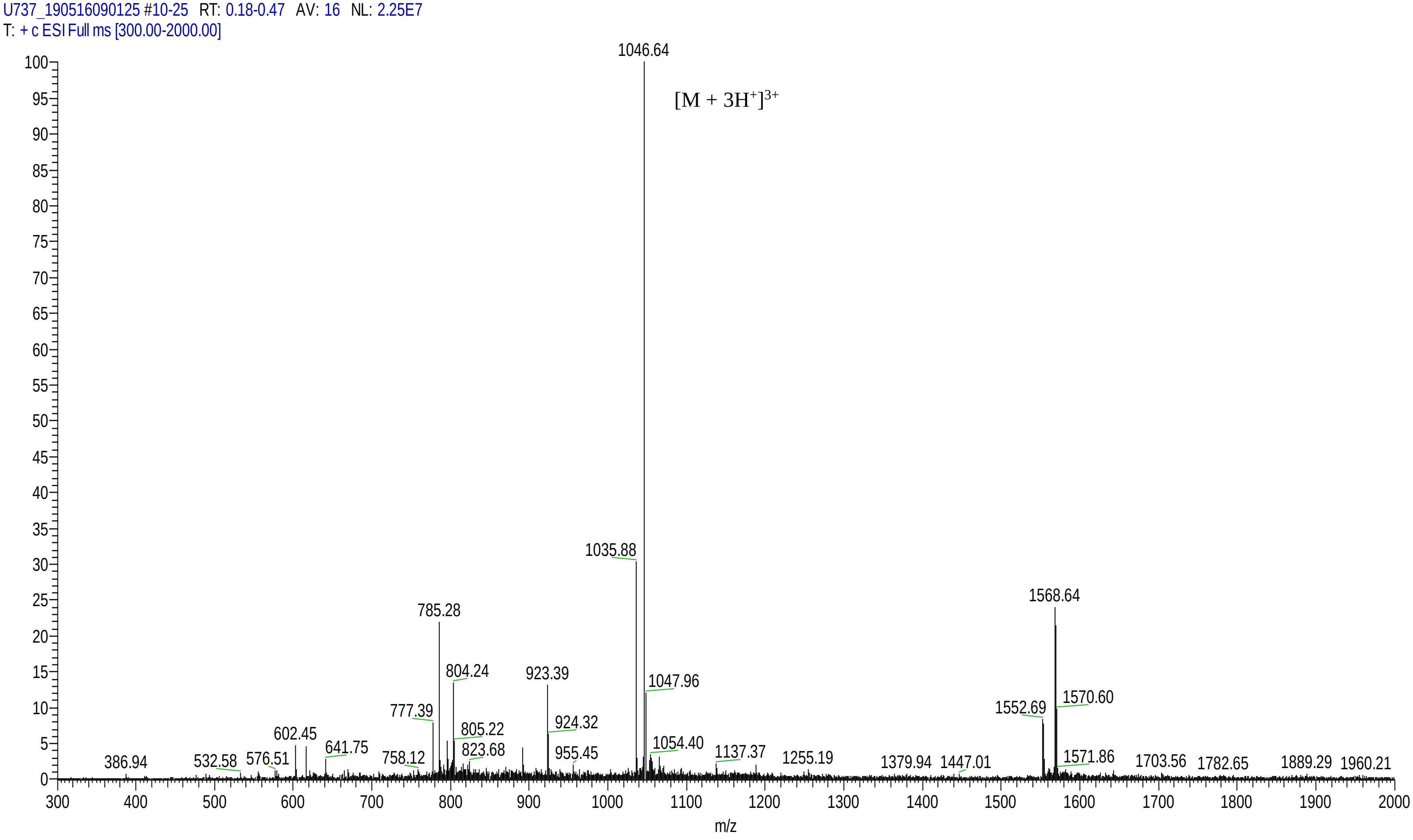

Sample Name: GT0396
Lot#: U737
Instrument 1 Agilent 1260
Instrument ID: RD-HPLC 1
Injection Date: 5/15/2019
Inj. Volume: 20.0 uL

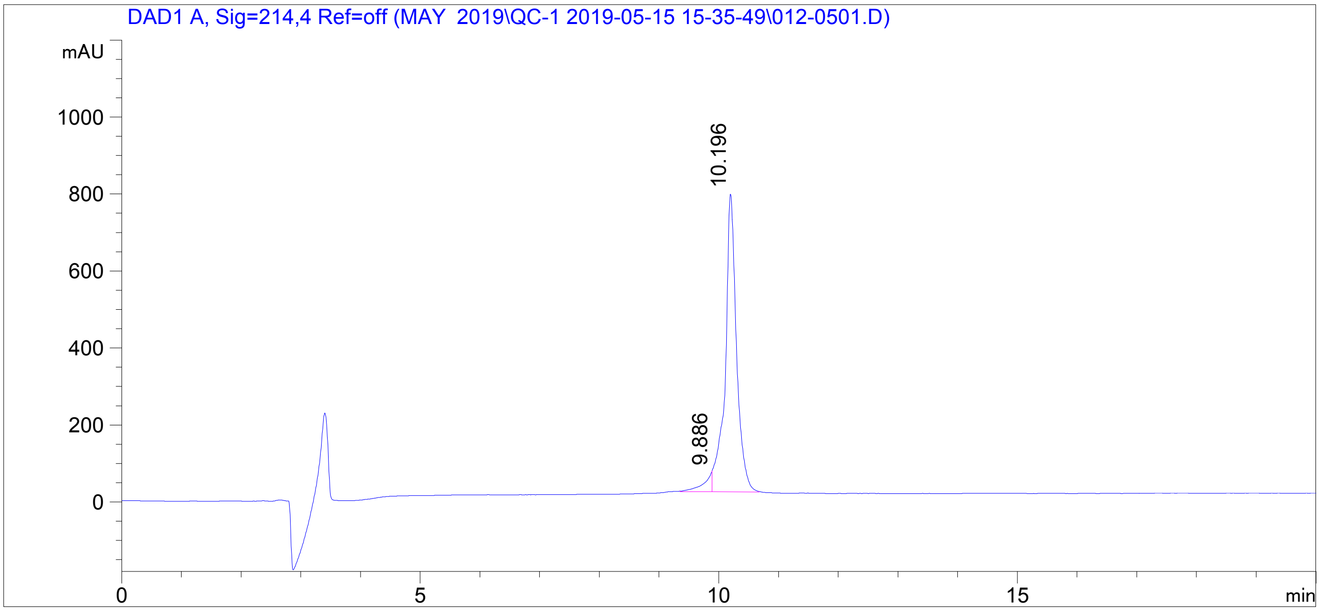

Data file name: C:\CHEM32\1\DATA\MAY 2019\QC-1 2019-05-15 15-35-49\012-0501.D

Acq. Method: C:\Chem32\1\DATA\May 2019\QC-1 2019-05-15 15-35-49\20-50-20.M

Column: Phenomenex Luna C18, 5um 250 x 4.6mm
Buffer A: 0.1% TFA in H2O
Buffer B: 0.1% TFA in ACN
Flow Rate: 1ml/min
Gradient: 20-50% B in 20 min

Peak #	RT [min]	Area	Height	Area %
1	9.886	503.96	53.92	4.70
2	10.196	10213.63	775.10	95.30

Peptide Content with Elemental Analysis

Analysis: Determination of Peptide Content by Nitrogen Content
Instrument Model: CE-440 Elemental Analyzer
Sample Name: N2L.EET03.L15.63 Gly-28-Gly
Sample ID: GT0396
Lot Number: U737
Sample Testing Date: 5/16/2019

	N%
**Expected Content**	17.42
**Actual Content**	13.75
**Peptide Content (%)**	**79.0**

Performed by:

5/16/2019

Name Date

Reviewed by:

5/16/2019

Name Date

CSBio
20 Kelly Court, Menlo Park, CA 94025 USA
Tel: 650-322-1111 Fax: 650-322-2278 Web: www.csbio.com Email: peptides@csbio.com
