# Supplementary material for: Disulfide-constrained peptide scaffolds enable a robust peptide-therapeutic discovery platform
Source: PLoS One. 2024 Mar 28;19(3):e0300135. doi: 10.1371/journal.pone.0300135 (PMC10977697; doi:10.1371/journal.pone.0300135)
Supplement: S1 File — A zip file contains 51 pdf files with filenames are the same as the “DCP name” listed in the tables. (ZIP) [file pone.0300135.s004.zip › N2L.EET03.n5.pdf]

SAMPLE TEST REPORT

Product: N2L.EET03.L1.n5 Gly-28-Gly
Sequence: Gly-Cys-Gly-Trp-Thr-Leu-Met-His-Cys-Lys-Gln-Asp-Ser-Asp-Cys-Leu-Ala-Gly-Cys-Val-Cys-Glu-Pro-Ile-Trp-Trp-Cys-Gly

Note: Natural Oxidation

Product No.: GT0443 Expected M.W.: 3096.63 Found M.W.: 3095.44 Lot: U845

APPEARANCE:	White Powder
MOLECULAR WEIGHT VERIFICATION:	Confirmed
PURITY: Instrument: Waters H Class<br>Condition: HPLC column in TFA System<br>Gradient: 20-50% Buffer B in 20 minutes<br>Buffer A: 0.1% TFA in H_2O<br>Buffer B: 0.1% TFA in ACN<br>Wavelength: 214 nm<br>Column: Phenomenex Luna C18 5µm 100Å, 4.6 x 250 mm	95.83%
PEPTIDE CONTENT:<br>(By N Elemental Analysis)	71.2%
ELLMAN’S TEST:	Complies
SUGGESTIONS FOR PEPTIDE DISSOLUTION:	Acetonitrile / Water
COUNTERIONS PRESENT:	TFA Salt
STORAGE:	All peptides should be stored dry at -20°C

This material is not listed as hazardous by ∗NIOSH/RTECS. Therefore, no SAFETY DATA SHEET is required. However, the chemical, physical and toxicological properties of this product have not been thoroughly investigated. Therefore, please exercise due care when handling this material. This action is in compliance with State and Federal OSHA standards and regulations.

Quality Control: ____________________

Date: July 17, 2019

CSBio
20 Kelly Court, Menlo Park, CA 94025 USA
T: (650) 322 1111 • F: (650) 322 2278
www.csbio.com • peptides@csbio.com

Compound: GT0443 N2L.EET03.L1.n5 Gly-28-Gly Lot Number: U845 Expected M.W.: 3096.63 Found M.W.: 3095.44

U845_190715072321 #70-82 RT: 1.43-1.68 AV: 13 NL: 5.85E5
T: + c ESI Full ms [300.00-2000.00]

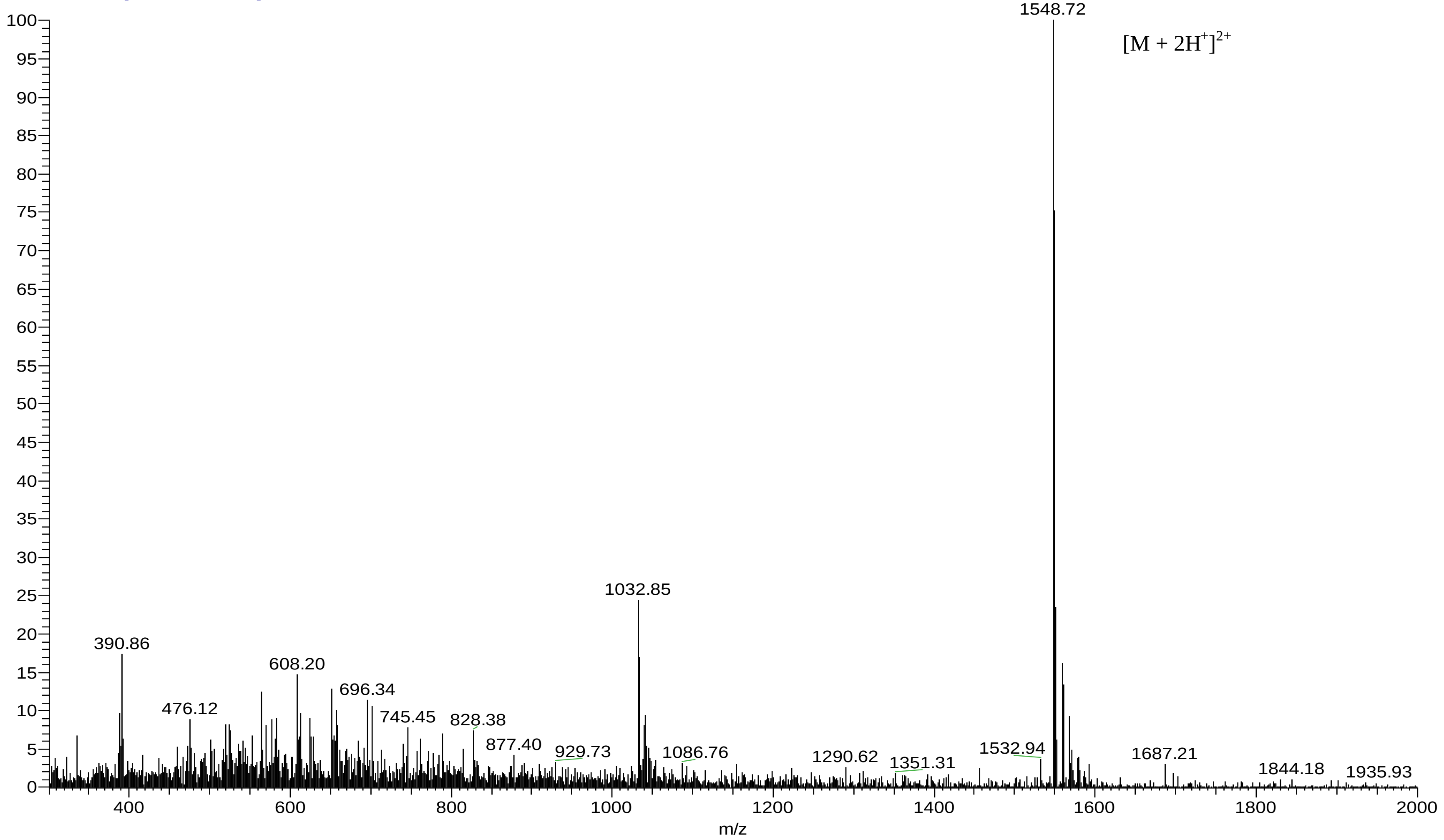

SAMPLE INFORMATION

Sample Name:	GT0443 U845	Acquired By:	RDQC
Sample Type:	Unknown	Sample Set Name	QC071019
Vial:	1:A.1	Acq. Method Set:	20_50_20min_214nm
Injection #:	1	Processing Method	RD QC
Injection Volume:	5.00 ul	Channel Name:	PDA Ch1 214nm@4.8nm
Run Time:	20.0 Minutes		PDA Ch1 214nm@4.8nm
Column	Phenomenex, Luna, C18(2), 5u 100A 250 x 4.6mm		
Date Acquired:	7/11/2019 7:46:46 PM PDT		
Date Processed:	7/12/2019 8:22:16 AM PDT		
Buffer:	A: 0.1% TFA in Water; B: 0.1% TFA in Acetonitrile		
Flow Rate:	1.0mL/min		

Auto-Scaled Chromatogram

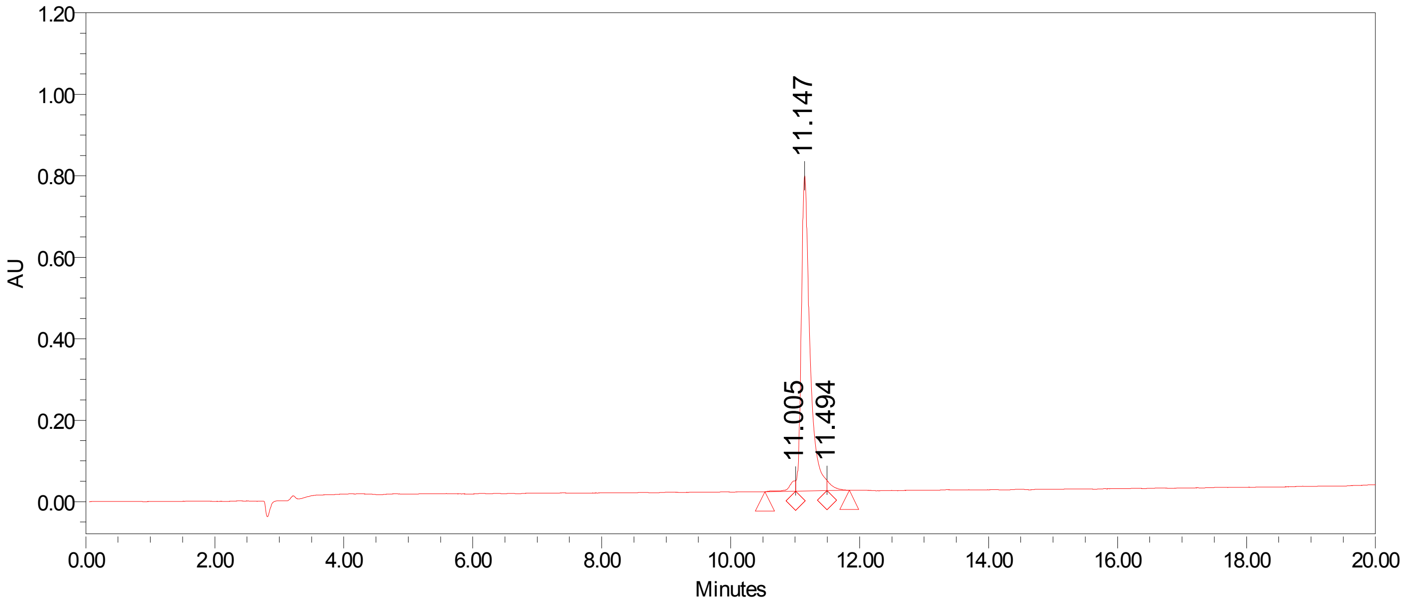

Peak Results

[redacted]					
[redacted]		11.005	172213	25235	2.29
		11.147	7208378	773892	95.83
		11.494	141456	25235	1.88

Peptide Content with Elemental Analysis

Analysis: Determination of Peptide Content by Nitrogen Content
Instrument Model: CE-440 Elemental Analyzer
Sample Name: N2L.EET03.L1.n5 Gly-28-Gly
Sample ID: GT0443
Lot Number: U845
Sample Testing Date: 7/15/2019

	N%
**Expected Content**	15.83
**Actual Content**	11.27
**Peptide Content (%)**	**71.2**

Performed by:

7/15/2019

Name Date

Reviewed by:

Shilpa Patel 7/15/2019

Name Date

CSBio
20 Kelly Court, Menlo Park, CA 94025 USA
Tel: 650-322-1111 Fax: 650-322-2278 Web: www.csbio.com Email: peptides@csbio.com
