# Supplementary material for: Disulfide-constrained peptide scaffolds enable a robust peptide-therapeutic discovery platform
Source: PLoS One. 2024 Mar 28;19(3):e0300135. doi: 10.1371/journal.pone.0300135 (PMC10977697; doi:10.1371/journal.pone.0300135)
Supplement: S1 File — A zip file contains 51 pdf files with filenames are the same as the “DCP name” listed in the tables. (ZIP) [file pone.0300135.s004.zip › N2L.EET31.32.13.pdf]

Quality Control Record

Product: N2.31.L2.32.L2.13 Gly-28-Gly
Sequence: Gly-Cys-Ile-Lys-Ser-His-Leu-Trp-Cys-Pro-Arg-Asp-Ser-Asp-Cys-Leu-Ala-Gly-Cys-Val-Cys-Glu-Val-Trp-Ile-Gln-Cys-Gly

Note: Natural Oxidation

Product No.: GT0622 Expected M.W.: 3076.61 Found M.W.: 3075.90 Lot: V450

APPEARANCE: White Powder

MOLECULAR WEIGHT VERIFICATION: Confirmed

PURITY: Instrument: Agilent 1260 — 84.15% (After Lyophilization)
Condition: HPLC column in TFA System
Gradient: 20-50% Buffer B in 20 minutes
Buffer A: 0.1% TFA in H_2O
Buffer B: 0.1% TFA in ACN
Wavelength: 214 nm
Column: Phenomenex Luna C18 5µm 100Å, 4.6 x 250 mm

PEPTIDE CONTENT: (By N Elemental Analysis) 86.7%

ELLMAN'S TEST: Complies

SUGGESTIONS FOR PEPTIDE DISSOLUTION: TFA / Acetonitrile / Water

COUNTERIONS PRESENT: TFA Salt

STORAGE: All peptides should be stored dry at -20°C

This material is not listed as hazardous by ∗NIOSH/RTECS. Therefore, no SAFETY DATA SHEET is required. However, the chemical, physical and toxicological properties of this product have not been thoroughly investigated. Therefore, please exercise due care when handling this material. This action is in compliance with State and Federal OSHA standards and regulations.

Quality Control: ______

Date: August 19, 2020

CSBio
20 Kelly Court, Menlo Park, CA 94025 USA
T: (650) 322 1111 • F: (650) 322 2278
www.csbio.com • peptides@csbio.com

Compound: GT0622 N2.31.L2.32.L2.13 Gly-28-Gly Lot Number: V450 Expected M.W.: 3076.61 Found M.W.: 3075.90

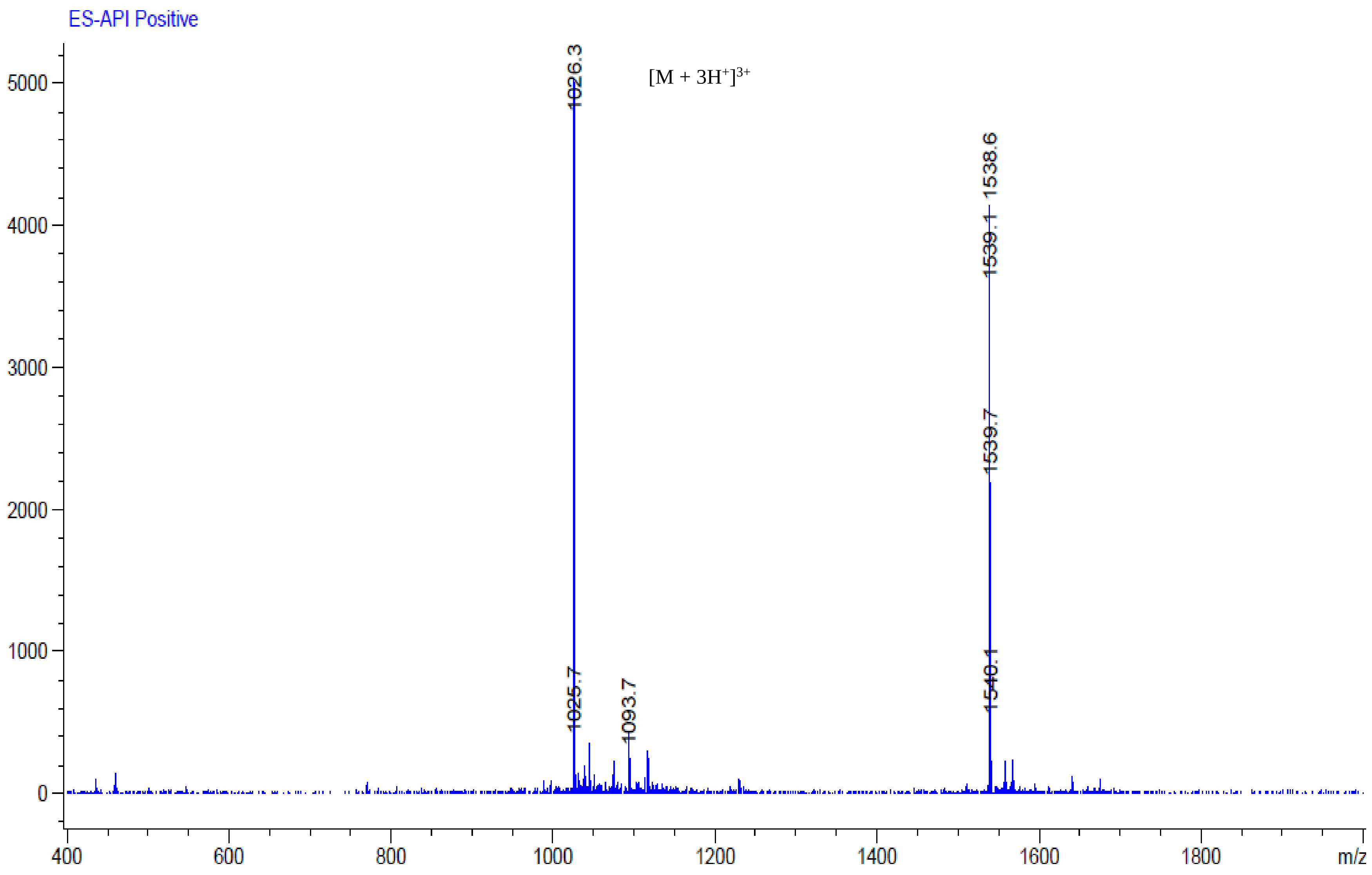

Sample Name: GT0622
Lot# V450
Instrument 1 Agilent 1260
Instrument ID: A004
Injection Date: 8/11/2020
Inj. Volume: 35.0 uL

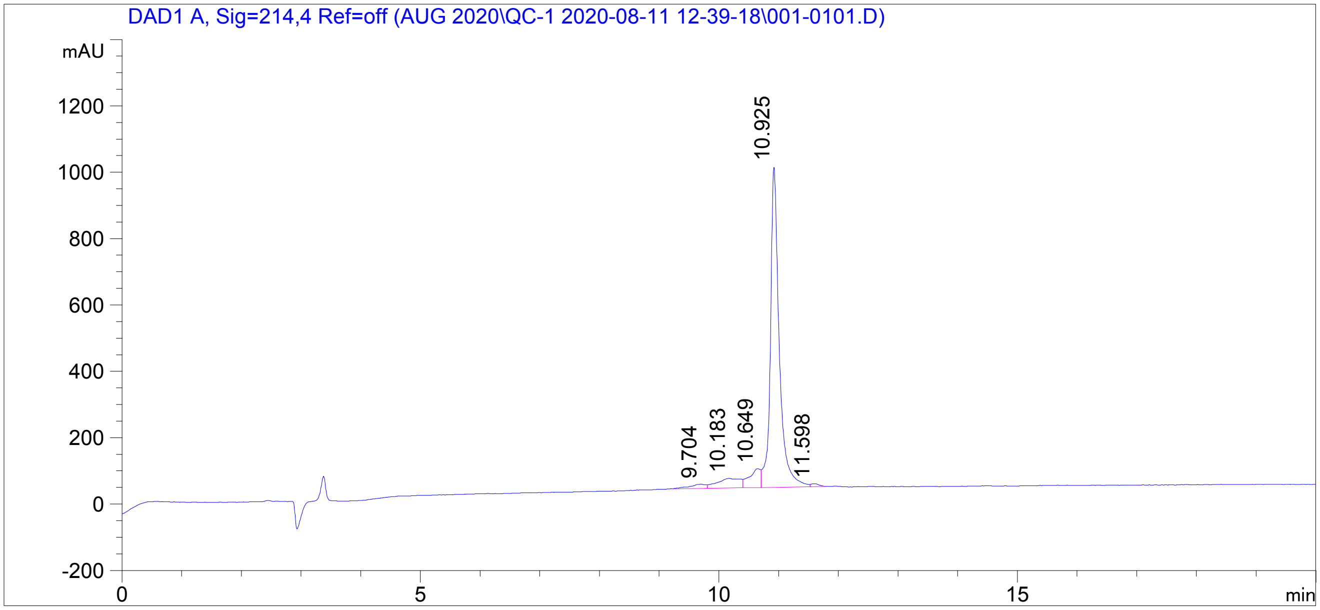

Data file name: C:\CHEM32\1\DATA\AUG 2020\QC-1 2020-08-11 12-39-18\001-0101.D
Acq. Method: C:\Chem32\1\DATA\AUG 2020\QC-1 2020-08-11 12-39-18\20-50-20.M

Column: Phenomenex Luna C18, 5um 250 x 4.6mm P/N: 00G-4252-E0
Buffer A: 0.1% TFA in H2O
Buffer B: 0.1% TFA in ACN
Wavelength: 214 nm
Flow Rate: 1ml/minute
Column Temperature: 25c
Gradient: 20%-50% B in 20 minutes

Peak #	RT [min]	Area	Height	Area %
1	9.704	247.86	12.62	2.10
2	10.183	783.93	28.77	6.65
3	10.649	748.51	56.98	6.35
4	10.925	9914.81	966.82	84.15
5	11.598	87.88	9.10	0.75

Peptide Content with Elemental Analysis

Analysis: Determination of Peptide Content by Nitrogen Content
Instrument Model: Perkin Elmer Series II CHNS/O Analyser
Sample Name: N2.31.L2.32.L2.13 Gly-28-Gly
Sample ID: GT0622
Lot Number: V450
Sample Testing Date: 8/14/2020

	N%
**Expected Content**	16.85
**Actual Content**	14.60
**Peptide Content (%)**	**86.7**

Performed by:

8/14/2020

Name Date

Reviewed by:

8/14/2020

Name Date

CSBio
20 Kelly Court, Menlo Park, CA 94025 USA
Tel: 650-322-1111 Fax: 650-322-2278 Web: www.csbio.com Email: peptides@csbio.com
