# Supplementary material for: Disulfide-constrained peptide scaffolds enable a robust peptide-therapeutic discovery platform
Source: PLoS One. 2024 Mar 28;19(3):e0300135. doi: 10.1371/journal.pone.0300135 (PMC10977697; doi:10.1371/journal.pone.0300135)
Supplement: S1 File — A zip file contains 51 pdf files with filenames are the same as the “DCP name” listed in the tables. (ZIP) [file pone.0300135.s004.zip › N2L.EET31.43.24.pdf]

Quality Control Record

Product: N2.31.L34.43.L2.24 Gly-28-Gly
Sequence: Gly-Cys-Ile-Lys-Ser-His-Leu-Trp-Cys-Pro-Lys-Thr-Ser-Asp-Cys-Glu-Ala-Gly-Cys-Ile-Cys-Glu-Val-Trp-Ile-Gln-Cys-Gly

Note: Natural Oxidation

Product No.: GT0617 Expected M.W.: 3064.16 Found M.W.: 3063.99 Lot: V442

APPEARANCE:	White Powder
MOLECULAR WEIGHT VERIFICATION:	Confirmed
PURITY: Instrument: Agilent 1260<br>Condition: HPLC column in Base System<br>Gradient: 0-60% Buffer B in 20 minutes<br>Buffer A: 0.1% NH_4OH in H_2O<br>Buffer B: 100% ACN<br>Wavelength: 214 nm<br>Column: Phenomenex Luna C18 5µm 100Å, 4.6 x 250 mm	90.34%
PEPTIDE CONTENT:<br>(By N Elemental Analysis)	Pending
ELLMAN'S TEST:	Complies
SUGGESTIONS FOR PEPTIDE DISSOLUTION:	Acetonitrile/0.1% Ammonium Hydroxide in Water
COUNTERIONS PRESENT:	TFA Salt
STORAGE:	All peptides should be stored dry at -20°C

This material is not listed as hazardous by ∗NIOSH/RTECS. Therefore, no SAFETY DATA SHEET is required. However, the chemical, physical and toxicological properties of this product have not been thoroughly investigated. Therefore, please exercise due care when handling this material. This action is in compliance with State and Federal OSHA standards and regulations.

Quality Control: ______________

Date: August 12, 2020

CSBio
20 Kelly Court, Menlo Park, CA 94025 USA
T: (650) 322 1111 • F: (650) 322 2278
www.csbio.com • peptides@csbio.com

Compound: GT0617 N2.31.L34.43.L2.24 Gly-28-Gly Lot Number: V442 Expected M.W.: 3064.16 Found M.W.: 3063.99

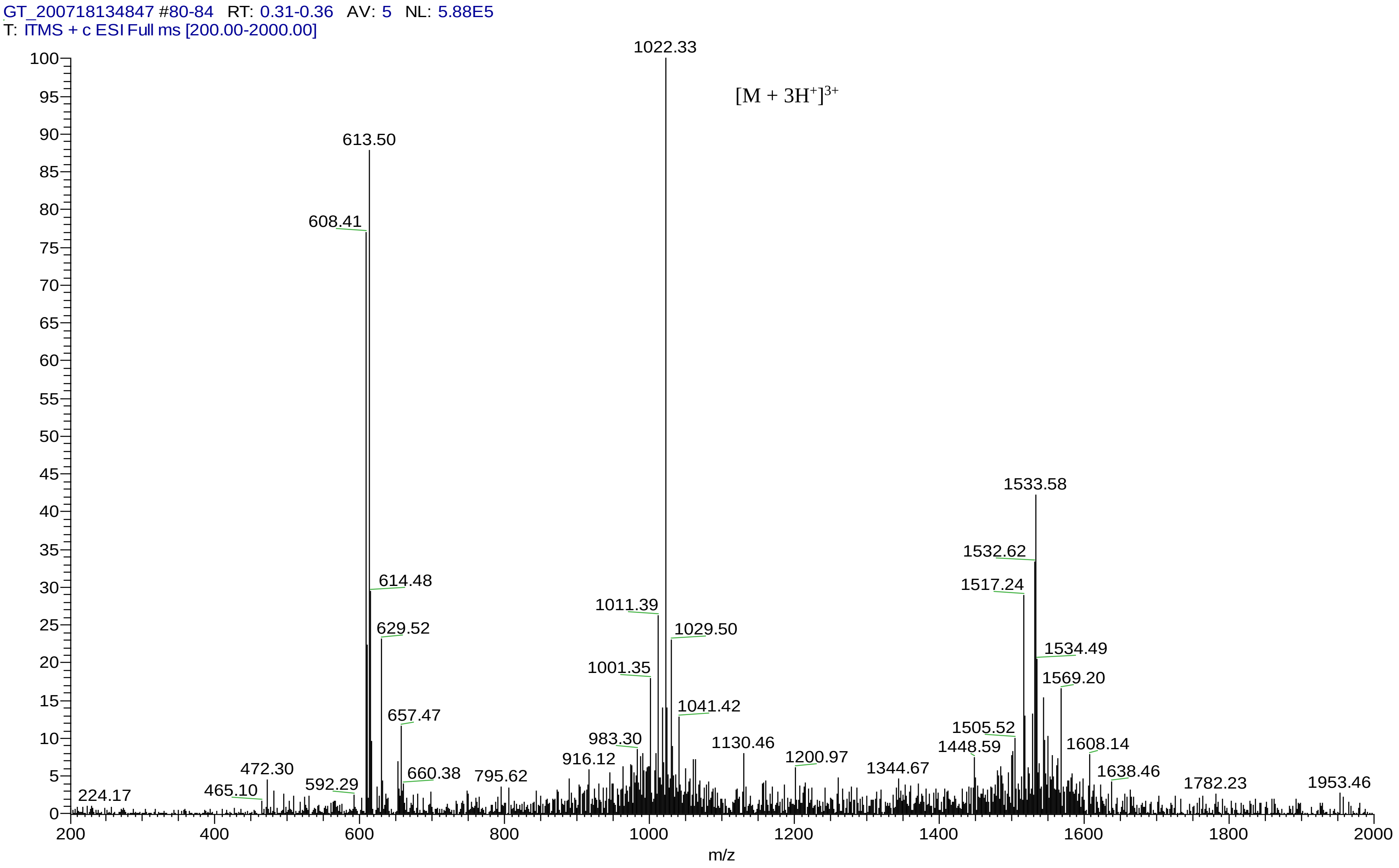

Sample Name: GT0617
Lot# V442
Instrument 1 Agilent 1260
Instrument ID: A004
Injection Date: 7/18/2020
Inj. Volume: 100.0uL

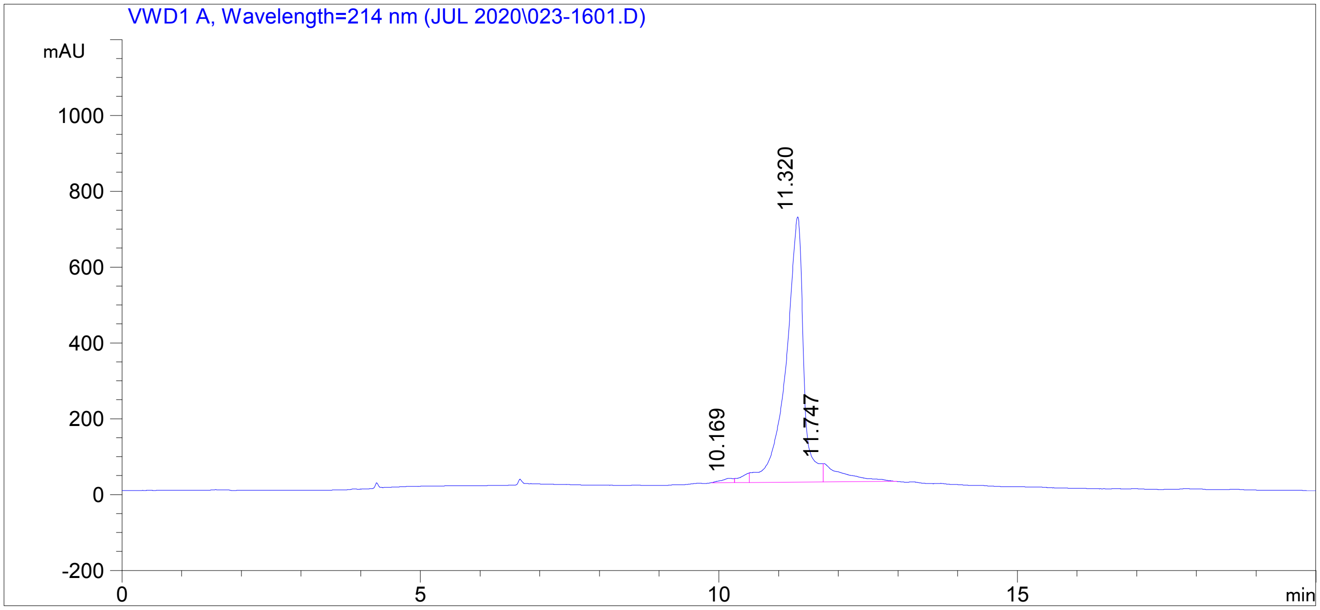

Data file name: C:\CHEM32\1\DATA\JUL 2020\023-1601.D
Acq. Method: C:\Chem32\1\DATA\JUL2020\WG20202020-07-1809-08-33\CD0-60-20.M

Column: Phenomenex Luna C18 5µm 100Å, 250x4.6mm
Buffer A: 0.1% NH4OH in H2O
Buffer B: 100% ACN
Wavelength: 214nm
Flow Rate: 1ml/minute
Column Temperature: 25c
Gradient: 0%-60% B in 20 minutes

Peak #	RT [min]	Area	Height	Area %
1	10.169	162.28	11.37	1.01
2	10.503	245.71	25.03	1.53
3	11.320	14500.30	699.55	90.34
4	11.747	1142.21	48.58	7.12
