# Supplementary material for: Disulfide-constrained peptide scaffolds enable a robust peptide-therapeutic discovery platform
Source: PLoS One. 2024 Mar 28;19(3):e0300135. doi: 10.1371/journal.pone.0300135 (PMC10977697; doi:10.1371/journal.pone.0300135)
Supplement: S1 File — A zip file contains 51 pdf files with filenames are the same as the “DCP name” listed in the tables. (ZIP) [file pone.0300135.s004.zip › N2L.EET31.43.43.pdf]

Quality Control Record

Product: N2.31.L34.43.L15.43 Gly-28-Gly
Sequence: Gly-Cys-Ile-Arg-Ser-His-Val-Trp-Cys-Lys-Gln-Asp-Ser-Asp-Cys-Glu-Ala-Gly-Cys-Ile-Cys-Glu-Ile-Trp-Ile-Gln-Cys-Gly

Note: Natural Oxidation

Product No.: GT0632 Expected M.W.: 3137.61 Found M.W.: 3136.46 Lot: V321

APPEARANCE:	White Powder
MOLECULAR WEIGHT VERIFICATION:	Confirmed
PURITY: Instrument: Agilent 1260<br>Condition: HPLC column in TFA System<br>Gradient: 20-50% Buffer B in 20 minutes<br>Buffer A: 0.1% TFA in H_2O<br>Buffer B: 0.1% TFA in ACN<br>Wavelength: 214 nm<br>Column: Phenomenex Luna C18 5µm 100Å, 4.6 x 250 mm	87.45% (After Lyophilization)
PEPTIDE CONTENT:<br>(By N Elemental Analysis)	81.6%
ELLMAN'S TEST:	Complies
SUGGESTIONS FOR PEPTIDE DISSOLUTION:	Acetonitrile / Water
COUNTERIONS PRESENT:	TFA Salt
STORAGE:	All peptides should be stored dry at -20°C

This material is not listed as hazardous by ∗NIOSH/RTECS. Therefore, no SAFETY DATA SHEET is required. However, the chemical, physical and toxicological properties of this product have not been thoroughly investigated. Therefore, please exercise due care when handling this material. This action is in compliance with State and Federal OSHA standards and regulations.

Quality Control: ____________

Date: June 2, 2020

CSBio
20 Kelly Court, Menlo Park, CA 94025 USA
T: (650) 322 1111 • F: (650) 322 2278
www.csbio.com • peptides@csbio.com

Compound: GT0632 N2.31.L34.43.L15.43 Gly-28-Gly Lot Number: V321 Expected M.W.: 3137.61 Found M.W.: 3136.46

V321_200529072752 #2-10 RT: 0.01-0.11 AV: 9 NL: 1.35E4
T: ITMS + c ESI Full ms [300.00-2000.00]

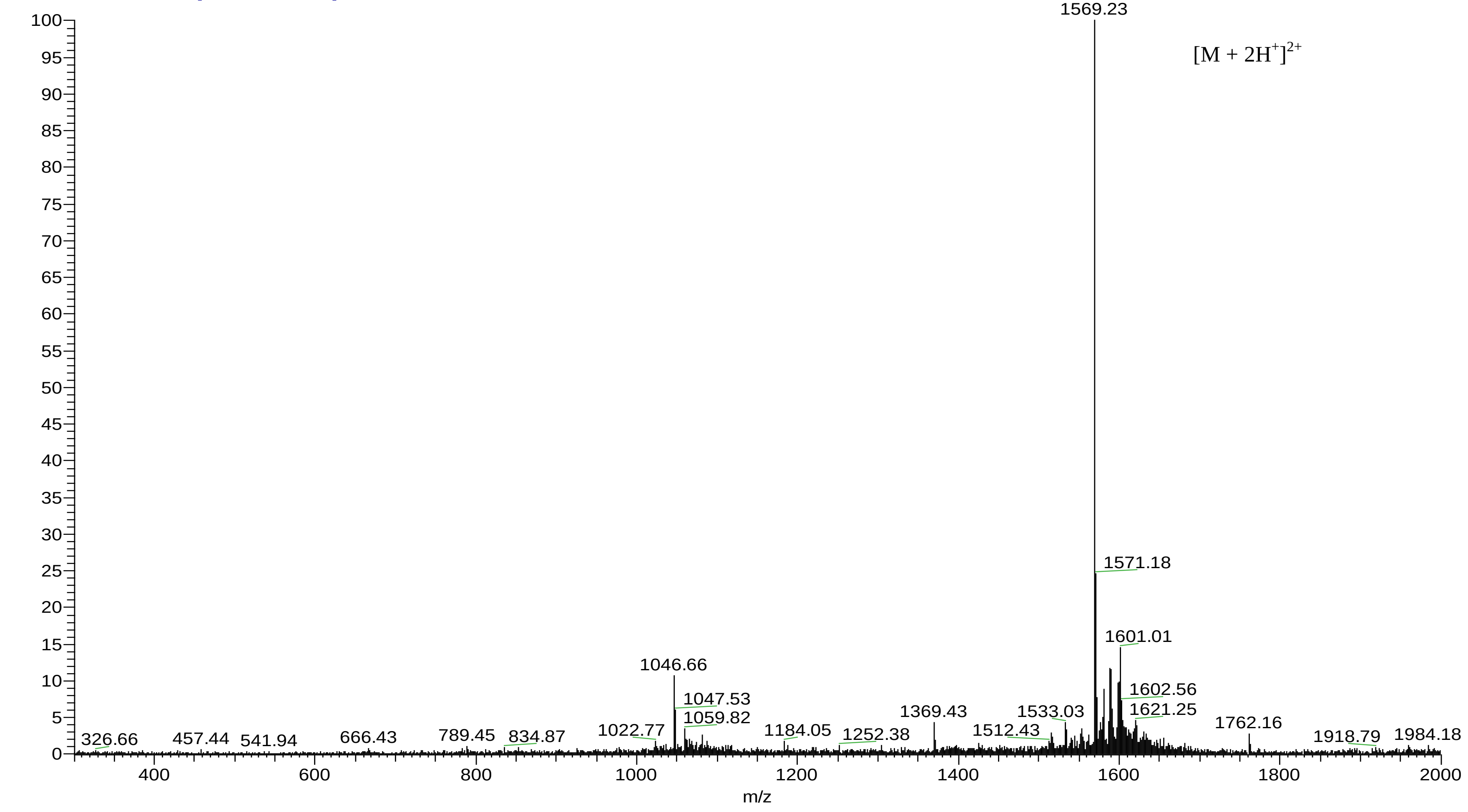

Sample Name: GT0632
Lot# V321
Instrument 1 Agilent 1260
Instrument ID: A004
Injection Date: 5/28/2020
Inj. Volume: 35.0 uL

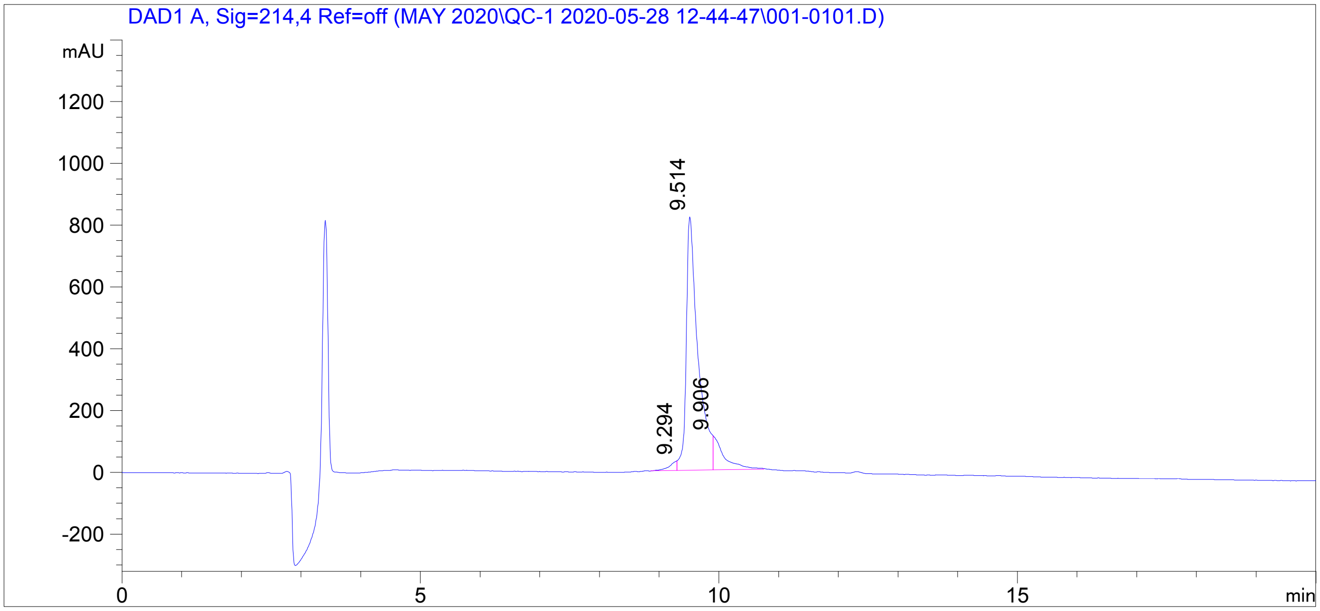

Data file name: C:\CHEM32\1\DATA\MAY 2020\QC-1 2020-05-28 12-44-47\001-0101.D
Acq. Method: C:\Chem32\1\DATA\MAY 2020\QC-1 2020-05-28 12-44-47\20-50-20.M

Column: Phenomenex Luna C18, 5um 250 x 4.6mm P/N: 00G-4252-E0
Buffer A: 0.1% TFA in H2O
Buffer B: 0.1% TFA in ACN
Wavelength: 214 nm
Flow Rate: 1ml/minute
Column Temperature: 25c
Gradient: 20%-50% B in 20 minutes

Peak #	RT [min]	Area	Height	Area %
1	9.294	264.77	30.92	2.04
2	9.514	11345.00	820.97	87.45
3	9.906	1364.01	110.29	10.51

Peptide Content with Elemental Analysis

Analysis: Determination of Peptide Content by Nitrogen Content
Instrument Model: Perkin Elmer Series II CHNS/O Analyser
Sample Name: N2.31.L34.43.L15.43 Gly-28-Gly
Sample ID: GT0632
Lot Number: V321
Sample Testing Date: 6/2/2020

	N%
**Expected Content**	16.96
**Actual Content**	13.84
**Peptide Content (%)**	**81.6**

Performed by:

6/2/2020

Name Date

Reviewed by:

6/2/2020

Name Date

CSBio
20 Kelly Court, Menlo Park, CA 94025 USA
Tel: 650-322-1111 Fax: 650-322-2278 Web: www.csbio.com Email: peptides@csbio.com
