# Supplementary material for: Disulfide-constrained peptide scaffolds enable a robust peptide-therapeutic discovery platform
Source: PLoS One. 2024 Mar 28;19(3):e0300135. doi: 10.1371/journal.pone.0300135 (PMC10977697; doi:10.1371/journal.pone.0300135)
Supplement: S1 File — A zip file contains 51 pdf files with filenames are the same as the “DCP name” listed in the tables. (ZIP) [file pone.0300135.s004.zip › N2L.EET31.43.75.pdf]

Quality Control Record

Product: N2.31.L34.43.L2.75 Gly-28-Gly
Sequence: Gly-Cys-Ile-Lys-Ser-His-Leu-Trp-Cys-Asp-Pro-Arg-Lys-Asp-Cys-Glu-Ala-Gly-Cys-Ile-Cys-Glu-Val-Trp-Ile-Gln-Cys-Gly

Note: Natural Oxidation

Product No.: GT0616   Expected M.W.: 3147.70   Found M.W.: 3148.77   Lot: V481

APPEARANCE: White Powder

MOLECULAR WEIGHT VERIFICATION: Confirmed

PURITY: Instrument: Agilent 1260 — 80.60% (After Lyophilization)
Condition: HPLC column in Base System
Gradient: 0-60% Buffer B in 20 minutes
Buffer A: 0.03% NH_4OH in H_2O
Buffer B: 100% ACN
Wavelength: 214 nm
Column: Phenomenex Luna C18 5µm 100Å, 4.6 x 250 mm

PEPTIDE CONTENT: (By N Elemental Analysis) Pending

ELLMAN'S TEST: Complies

SUGGESTIONS FOR PEPTIDE DISSOLUTION: 0.1% Ammonium Hydroxide in Water

COUNTERIONS PRESENT: TFA Salt

STORAGE: All peptides should be stored dry at -20°C

This material is not listed as hazardous by ∗NIOSH/RTECS. Therefore, no SAFETY DATA SHEET is required. However, the chemical, physical and toxicological properties of this product have not been thoroughly investigated. Therefore, please exercise due care when handling this material. This action is in compliance with State and Federal OSHA standards and regulations.

Quality Control: ____________

Date: August 26, 2020

CSBio
20 Kelly Court, Menlo Park, CA 94025 USA
T: (650) 322 1111 • F: (650) 322 2278
www.csbio.com • peptides@csbio.com

Compound: GT0616 | N2.31.L34.43.L2.75 Gly-28-Gly | Lot Number: V481 | Expected M.W.: 3147.70 | Found M.W.: 3148.77

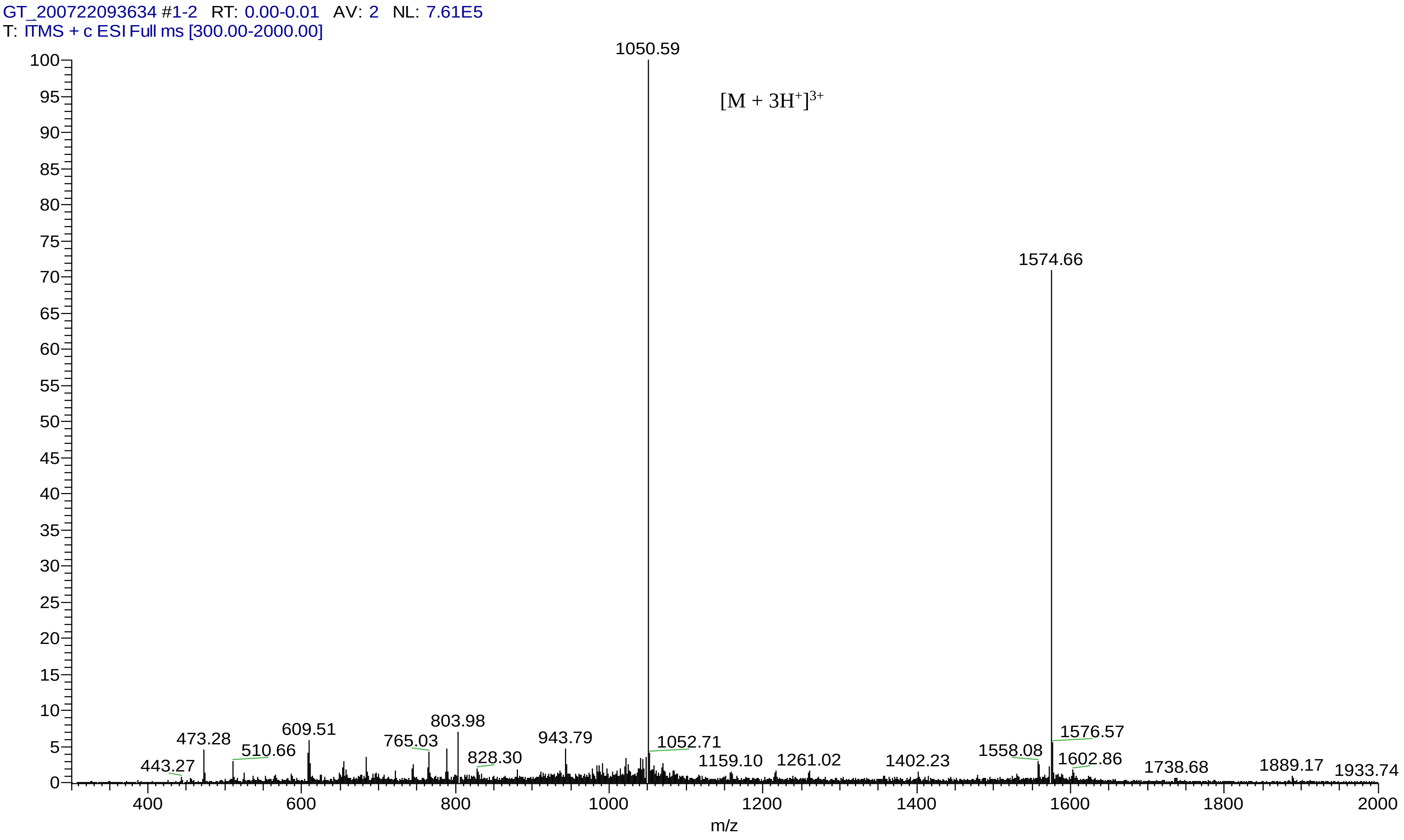

Sample Name: GT0616
Lot# V481
Instrument 1 Agilent 1260
Instrument ID: A004
Injection Date: 7/10/2020
Inj. Volume: 100.0uL

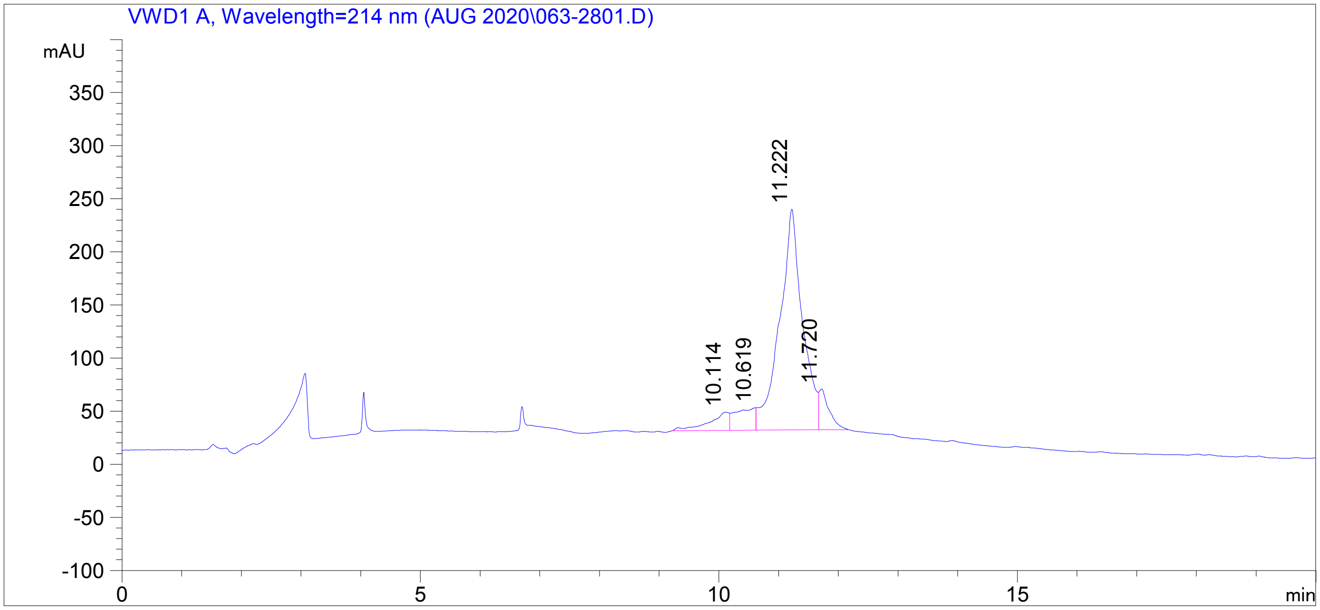

Data file name: C:\CHEM32\1\DATA\AUG 2020\063-2801.D
Acq. Method: C:\Chem32\1\DATA\JUL2020\WG20202020-07-1007-03-58\CD0-60-20.M

Column: Phenomenex Luna C18 5µm 100Å, 250x4.6mm
Buffer A: 0.03% NH4OH in H2O
Buffer B: 100% ACN
Wavelength: 214nm
Flow Rate: 1ml/minute
Column Temperature: 25c
Gradient: 0%-60% B in 20 minutes

Peak #	RT [min]	Area	Height	Area %
1	10.114	399.08	17.13	5.80
2	10.619	489.48	21.24	7.11
3	11.222	5547.23	207.91	80.60
4	11.720	446.52	38.42	6.49
