# Supplementary material for: Disulfide-constrained peptide scaffolds enable a robust peptide-therapeutic discovery platform
Source: PLoS One. 2024 Mar 28;19(3):e0300135. doi: 10.1371/journal.pone.0300135 (PMC10977697; doi:10.1371/journal.pone.0300135)
Supplement: S1 File — A zip file contains 51 pdf files with filenames are the same as the “DCP name” listed in the tables. (ZIP) [file pone.0300135.s004.zip › N2N-AVR-n6.pdf]

Quality Control Record

Product: N2N-AVR-n6 Ala-34-Arg
Sequence: Ala-Phe-Asp-Cys-Leu-Gly-Gln-Cys-Gly-Arg-Cys-Asp-Phe-His-Lys-Leu-Gln-Cys-Val-Trp-Ser-Asn-Arg-His-Ser-Pro-Tyr-Cys-Asn-Ser-Ser-Cys-Thr-Arg

Note: Natural Oxidation

Product No.: GT0367 Expected M.W.: 3917.38 Found M.W.: 3916.20 Lot: V607

APPEARANCE:	White Powder
MOLECULAR WEIGHT VERIFICATION:	Confirmed
PURITY: Instrument: Waters H Class<br>Condition: HPLC column in TFA System<br>Gradient: 20-50% Buffer B in 20 minutes<br>Buffer A: 0.1% TFA in H_2O<br>Buffer B: 0.1% TFA in ACN<br>Wavelength: 214 nm<br>Column: Phenomenex Luna C18 5µm 100Å, 4.6 x 250 mm	95.24%
PEPTIDE CONTENT:<br>(By N Elemental Analysis)	75.9%
ELLMAN'S TEST:	Complies
SUGGESTIONS FOR PEPTIDE DISSOLUTION:	Water
COUNTERIONS PRESENT:	TFA Salt
STORAGE:	All peptides should be stored dry at -20°C

This material is not listed as hazardous by ∗NIOSH/RTECS. Therefore, no SAFETY DATA SHEET is required. However, the chemical, physical and toxicological properties of this product have not been thoroughly investigated. Therefore, please exercise due care when handling this material. This action is in compliance with State and Federal OSHA standards and regulations.

Quality Control: ____________

Date: October 23, 2020

CSBio
20 Kelly Court, Menlo Park, CA 94025 USA
T: (650) 322 1111 • F: (650) 322 2278
www.csbio.com • peptides@csbio.com

Compound: GT0367 N2N-AVR-n6 Ala-34-Arg Lot Number: V607 Expected M.W.: 3917.38 Found M.W.: 3916.20

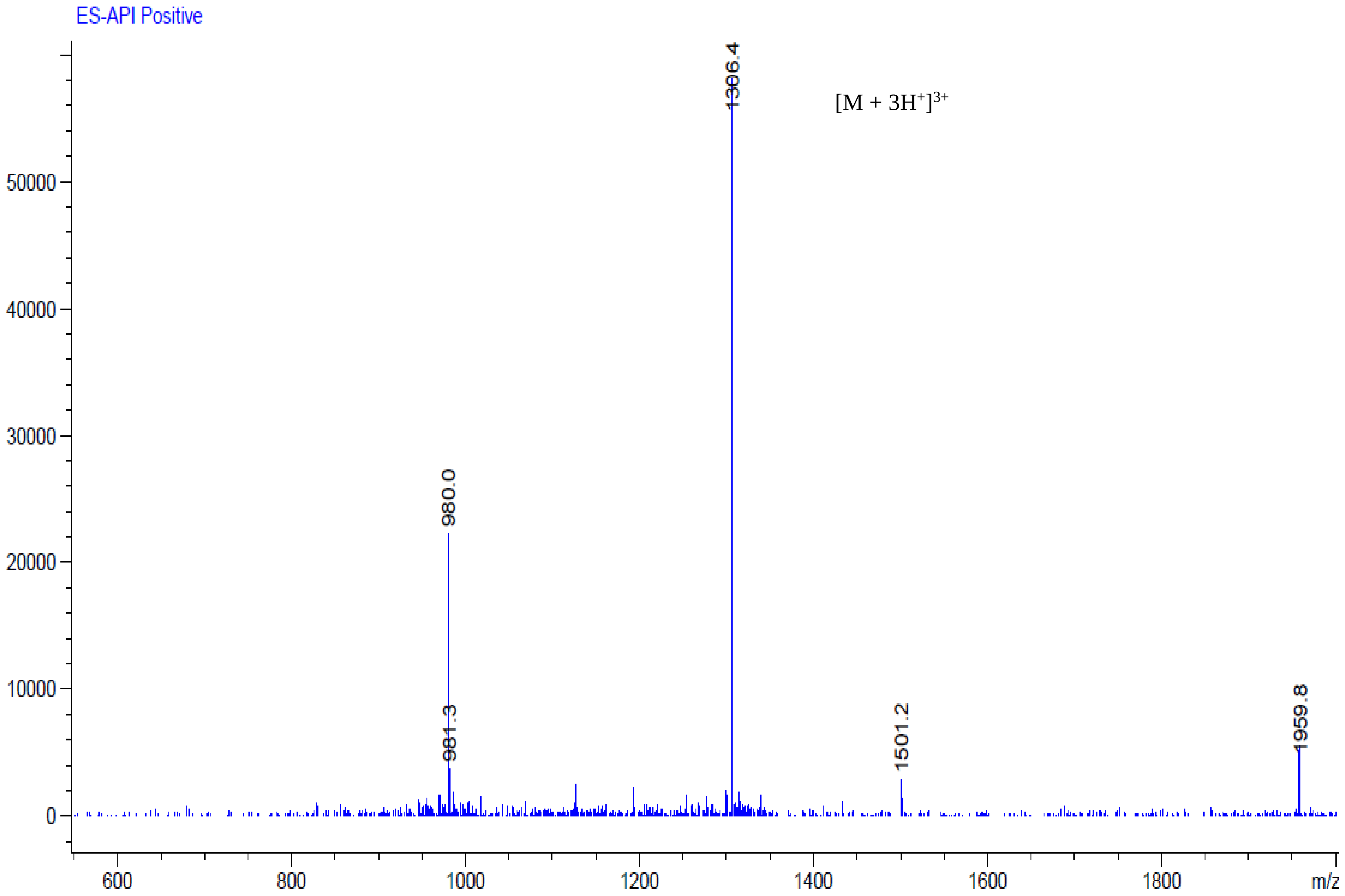

SAMPLE INFORMATION

Sample Name:	GT0367 V607	Acquired By:	RDQC
Sample Type:	Unknown	Sample Set Name	10082020
Vial:	1:B,1	Acq. Method Set:	20_50_20_214
Injection #:	1	Processing Method	RD QC
Injection Volume:	15.00 ul	Channel Name:	PDA Ch1 214nm@4.8nm
Run Time:	20.0 Minutes	Proc. Chnl. Descr.:	PDA Ch1 214nm@4.8nm
Date Acquired:	10/8/2020 11:38:45 AM PDT		
Date Processed:	10/8/2020 1:26:34 PM PDT		

Auto-Scaled Chromatogram

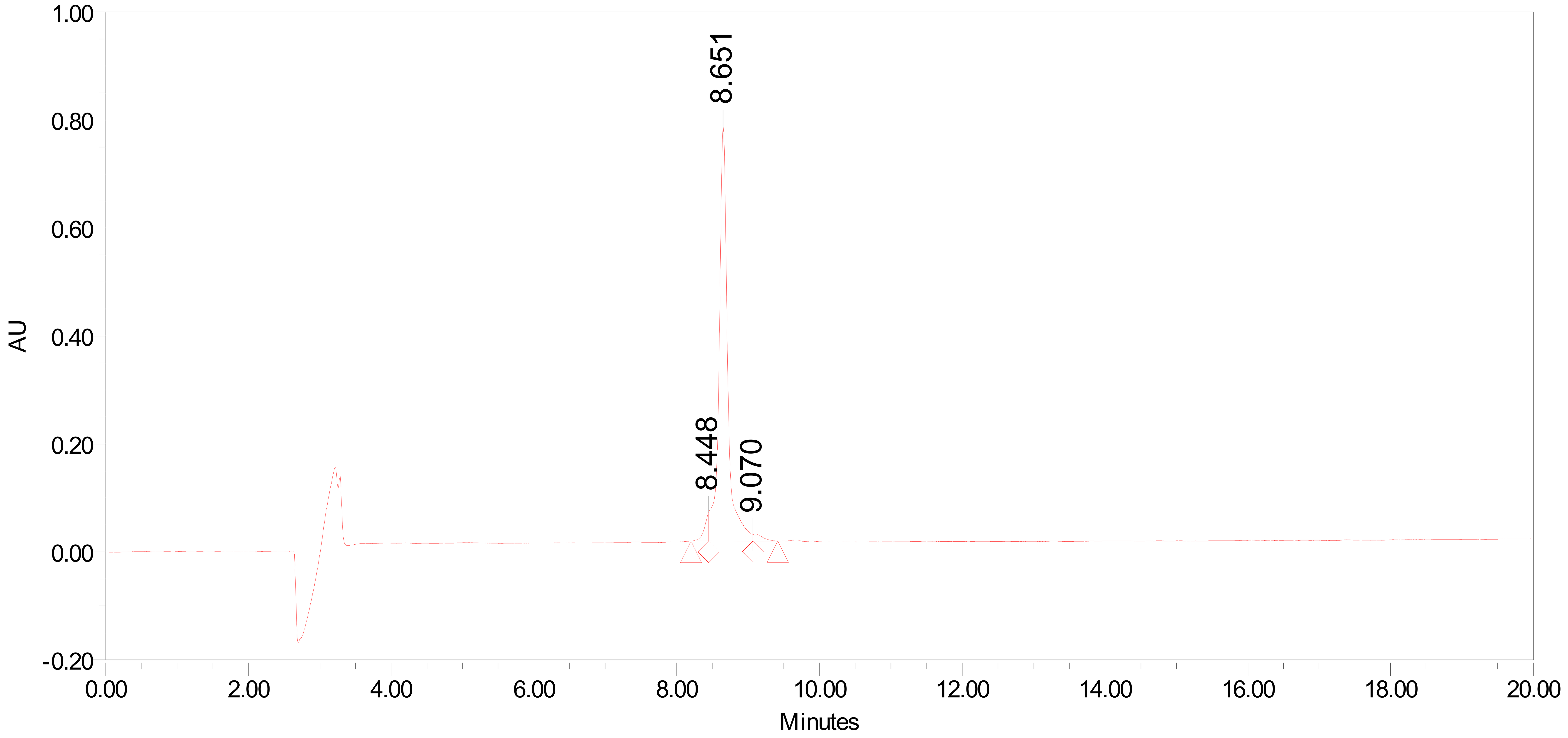

Peak Results

[redacted]					
[redacted]		8.448	209404	53431	3.10
[redacted]		8.651	6429510	769379	95.24
[redacted]		9.070	111920	11965	1.66

Peptide Content with Elemental Analysis

Analysis: Determination of Peptide Content by Nitrogen Content
Instrument Model: Perkin Elmer Series II CHNS/O Analyser 2400
Sample Name: N2N-AVR-n6 Ala-34-Arg
Sample ID: GT0367
Lot Number: V607
Sample Testing Date: 10/16/2020

	N%
**Expected Content**	18.95
**Actual Content**	14.39
**Peptide Content (%)**	**75.9**

Performed by:

10/16/2020

Name Date

Reviewed by:

10/16/2020

Name Date

CSBio
20 Kelly Court, Menlo Park, CA 94025 USA
Tel: 650-322-1111 Fax: 650-322-2278 Web: www.csbio.com Email: peptides@csbio.com
