# Supplementary material for: Disulfide-constrained peptide scaffolds enable a robust peptide-therapeutic discovery platform
Source: PLoS One. 2024 Mar 28;19(3):e0300135. doi: 10.1371/journal.pone.0300135 (PMC10977697; doi:10.1371/journal.pone.0300135)
Supplement: S1 File — A zip file contains 51 pdf files with filenames are the same as the “DCP name” listed in the tables. (ZIP) [file pone.0300135.s004.zip › N2N-EET-33.pdf]

SAMPLE TEST REPORT

Product: N2N-EET-33 Gly-32-Gly
Sequence: Gly-Cys-Gln-Gln-Arg-Arg-Trp-Gly-Gly-Trp-Gln-Gln-Cys-Lys-Gln-Asp-Ser-Asp-Cys-Leu-Ala-Gly-Cys-Val-Cys-Gly-Pro-Asn-Gly-Phe-Cys-Gly

Note: Natural Oxidation

Product No.: GT0263 Expected M.W.: 3442.85 Found M.W.: 3444.45 Lot: U080

APPEARANCE: White Powder

MOLECULAR WEIGHT VERIFICATION: Confirmed

PURITY: Instrument: Agilent 1260 System — 94.15% (Before Lyophilization)
Condition: HPLC column in TFA System
Gradient: 10-40% Buffer B in 20 minutes
Buffer A: 0.1% TFA in H_2O
Buffer B: 0.1% TFA in ACN
Wavelength: 214 nm
Column: Phenomenex Luna C18 5µm 100Å, 4.6 x 250 mm

PURITY: Instrument: Agilent 1260 System — 93.20% (After Lyophilization)
Condition: HPLC column in TFA System
Gradient: 15-45% Buffer B in 20 minutes
Buffer A: 0.1% TFA in H2O
Buffer B: 0.1% TFA in ACN
Wavelength: 214 nm
Column: Phenomenex Luna C18 5µm 100Å, 4.6 x 250 mm

ELLMAN'S TEST: Complies

CS Bio Co.
20 Kelly Court, Menlo Park, CA 94025 USA
T: (650) 322 1111 • F: (650) 322 2278
www.csbio.com • peptides@csbio.com

SAMPLE TEST REPORT

Product: N2N-EET-33 Gly-32-Gly
Sequence: Gly-Cys-Gln-Gln-Arg-Arg-Trp-Gly-Gly-Trp-Gln-Gln-Cys-Lys-Gln-Asp-Ser-Asp-Cys-Leu-Ala-Gly-Cys-Val-Cys-Gly-Pro-Asn-Gly-Phe-Cys-Gly

Note: Natural Oxidation

Product No.: GT0263 Expected M.W.: 3442.85 Found M.W.: 3444.45 Lot: U080

PEPTIDE CONTENT: 83.1%
(By Amino Acid Analysis)

SUGGESTIONS FOR PEPTIDE DISSOLUTION: Acetonitrile / 0.1% TFA in Water

COUNTERIONS PRESENT: TFA Salt

STORAGE: All peptides should be stored dry at -20°C

This material is not listed as hazardous by ∗NIOSH/RTECS. Therefore, no SAFETY DATA SHEET is required. However, the chemical, physical and toxicological properties of this product have not been thoroughly investigated. Therefore, please exercise due care when handling this material. This action is in compliance with State and Federal OSHA standards and regulations.

Quality Control: Xiaosong Jin

Date: October 10, 2018

CS Bio Co.
20 Kelly Court, Menlo Park, CA 94025 USA
T: (650) 322 1111 • F: (650) 322 2278
www.csbio.com • peptides@csbio.com

Compound: GT0263 N2N-EET-33 Gly-32-Gly Lot Number: U080 Expected M.W.: 3442.85 Found M.W.: 3444.45

U080_181005171113 #15-19 RT: 0.26-0.33 AV: 5 NL: 6.39E6
T: + c ESI Full ms [300.00-2000.00]

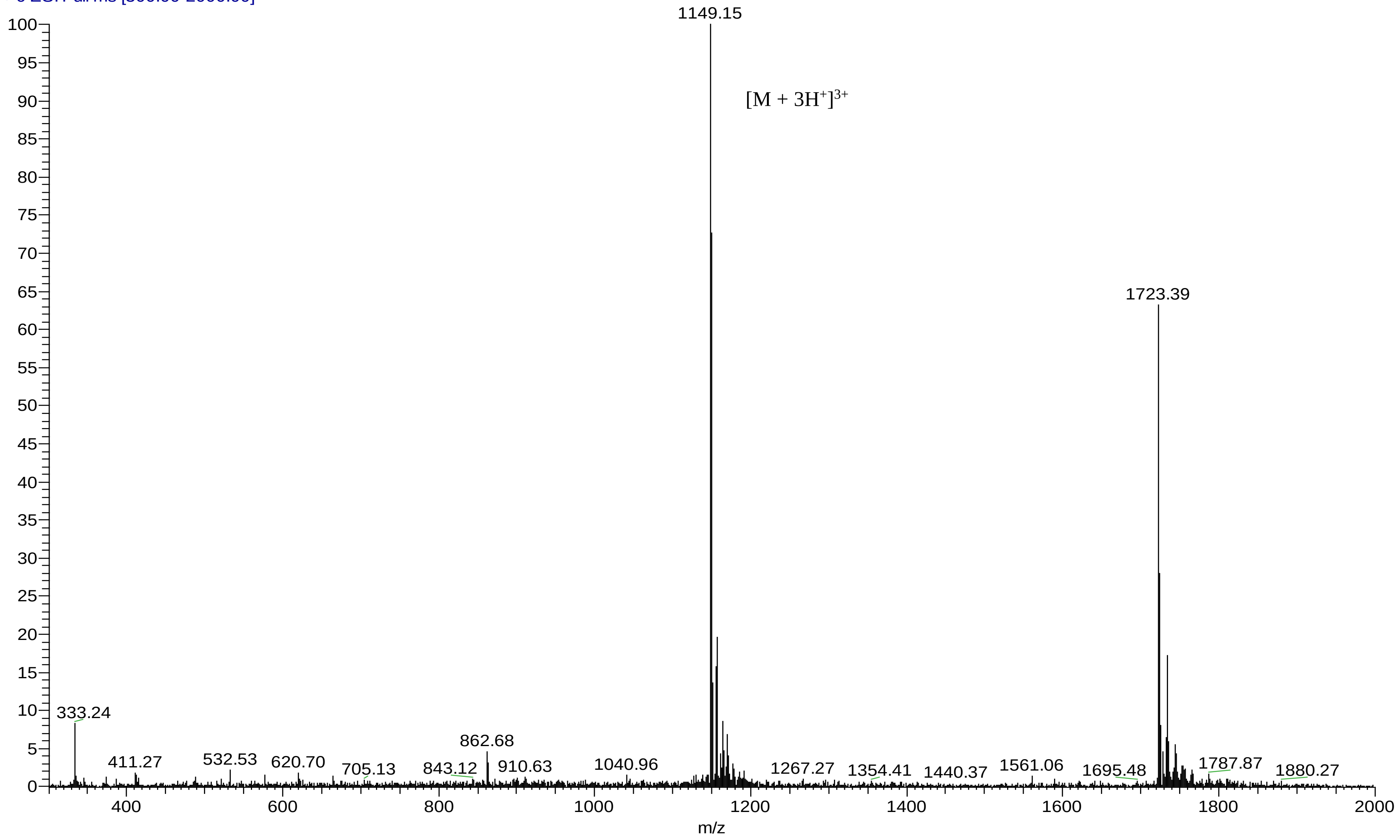

Sample Name: GT0263 (Before Lyophilization)
Lot#: U080
Instrument 1 Agilent 1260
Instrument ID: RD-HPLC 1
Injection Date: 10/1/2018
Inj. Volume: 12.0 uL

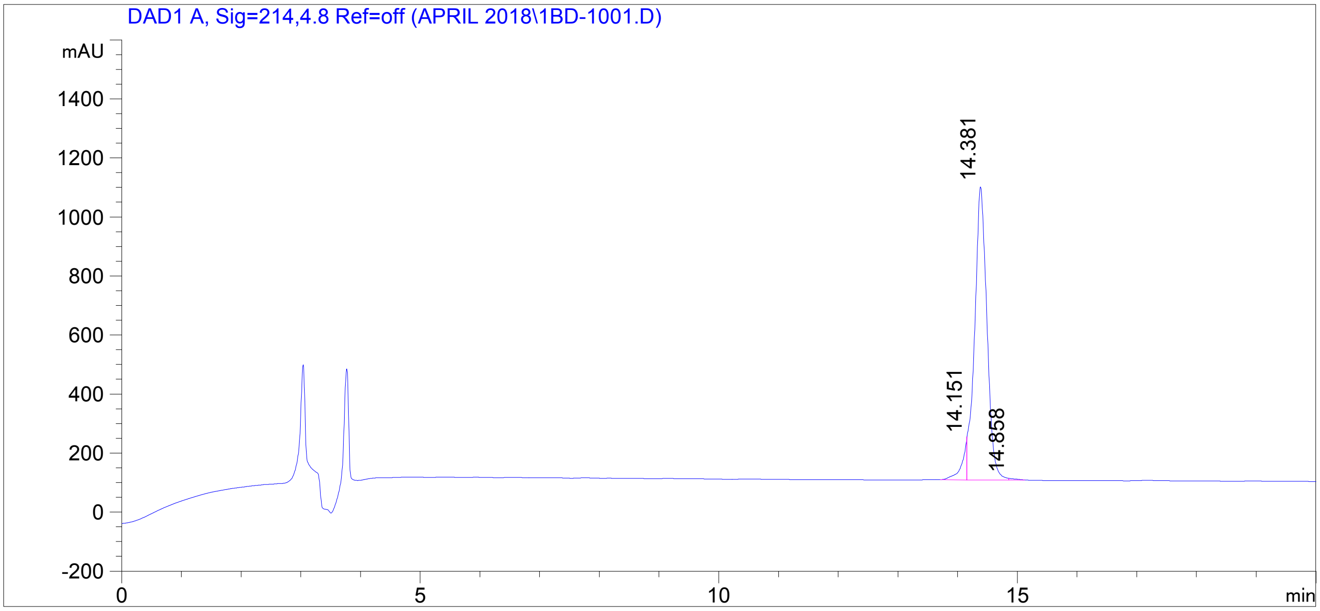

Data file name: C:\CHEM32\1\DATA\APRIL 2018\1BD-1001.D

Acq. Method: C:\Chem32\1\DATA\Sep2018\PURIFICATION 2018-10-01 14-19-01\10-40-20-1-6.M
Column: Phenomenex Luna C18(2) 5u 100A 250x4.6mm P/N: 00G-4252-E0
Buffer A: 0.1% TFA in H2O
Buffer B: 0.1% TFA in ACN
Flow Rate: 1ml/min
Gradient: 10-40% B in 20 min

Peak #	RT [min]	Area	Height	Area %
1	14.151	832.49	139.76	5.53
2	14.381	14163.68	994.33	94.15
3	14.858	46.82	5.83	0.31

Sample Name: GT0263
Lot#: U080
Instrument 1 Agilent 1260
Instrument ID: RD-HPLC 1
Injection Date: 10/5/2018
Inj. Volume: 50.0 uL

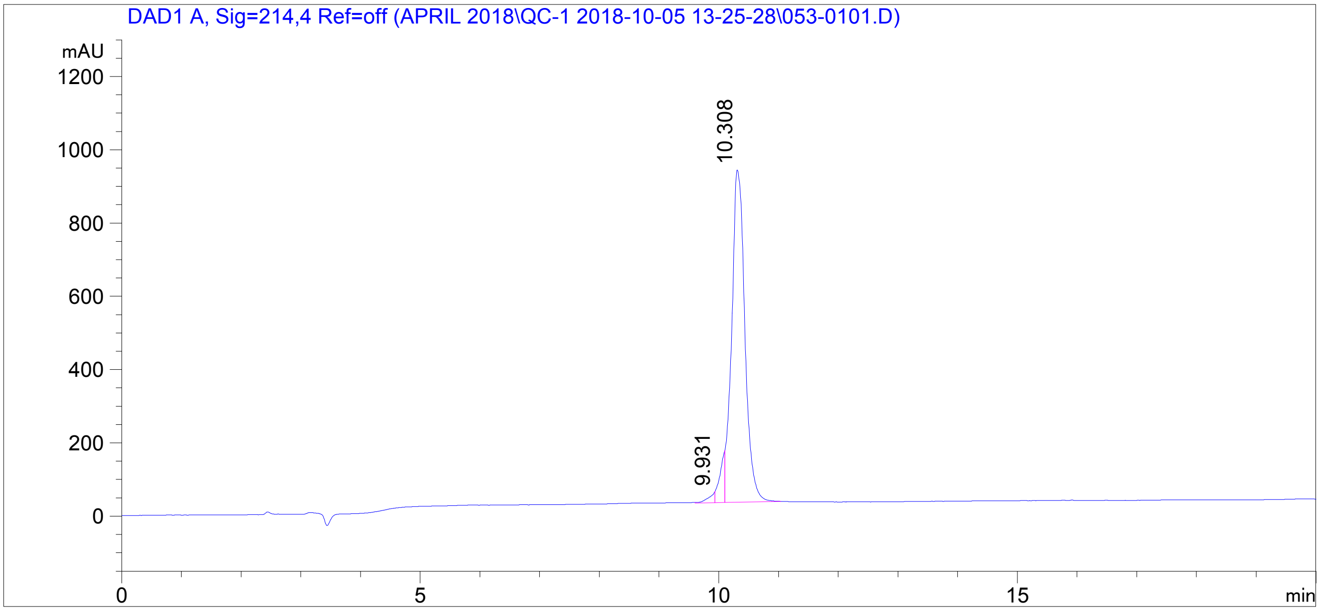

Data file name: C:\CHEM32\1\DATA\APRIL 2018\QC-1 2018-10-05 13-25-28\053-0101.D

Acq. Method: C:\Chem32\1\DATA\April 2018\QC-1 2018-10-05 13-25-28\15-45-20.M

Column: Phenomenex Luna C18 5u 100A 250x4.6mm P/N: 00G-4252-E0
Buffer A: 0.1% TFA in Water
Buffer B: 0.1% TFA in ACN
Flow Rate: 1 ml/min
Gradient: 15 to 45% B in 20 min

Peak #	RT [min]	Area	Height	Area %
1	9.931	207.80	29.31	1.39
2	10.098	810.83	140.37	5.41
3	10.308	13966.19	907.52	93.20

Peptide Content Determination by Amino Acid Analysis

Instrument Model: Waters H Class System
Sample Name: N2N-EET-33 Gly-32-Gly
Sample ID: GT0263
Lot No.: U080
Sample Testing Date: 10/09/2018

**Peptide Content (%)**	**83.1%**

Performed by:

Shilpa Patel · 10/09/2018

Name Date

Reviewed by:

Xiaosong Jin 10/09/2018

Name Date
