# Supplementary material for: Disulfide-constrained peptide scaffolds enable a robust peptide-therapeutic discovery platform
Source: PLoS One. 2024 Mar 28;19(3):e0300135. doi: 10.1371/journal.pone.0300135 (PMC10977697; doi:10.1371/journal.pone.0300135)
Supplement: S1 File — A zip file contains 51 pdf files with filenames are the same as the “DCP name” listed in the tables. (ZIP) [file pone.0300135.s004.zip › N2N-EET-45.pdf]

SAMPLE TEST REPORT

Product: N2N-EET-45 Gly-32-Gly
Sequence: Gly-Cys-Gly-Gln-Gln-Arg-Trp-Gly-Gly-Trp-Pro-Leu-Cys-Lys-Gln-Asp-Ser-Asp-Cys-Leu-Ala-Gly-Cys-Val-Cys-Gly-Pro-Asn-Gly-Phe-Cys-Gly

Note: Natural Oxidation

Product No.: GT0361 Expected M.W.: 3297.67 Found M.W.: 3297.63 Lot: U784

APPEARANCE: White Powder

MOLECULAR WEIGHT VERIFICATION: Confirmed

PURITY: Instrument: Agilent 1260 System — 95.75%
Condition: HPLC column in TFA System
Gradient: 20-50% Buffer B in 20 minutes
Buffer A: 0.1% TFA in H_2O
Buffer B: 0.1% TFA in ACN
Wavelength: 214 nm
Column: Phenomenex Luna C18 5µm 100Å, 4.6 x 250 mm

PEPTIDE CONTENT: (By N Elemental Analysis) Pending

ELLMAN'S TEST: Complies

SUGGESTIONS FOR PEPTIDE DISSOLUTION: Water

COUNTERIONS PRESENT: TFA Salt

STORAGE: All peptides should be stored dry at -20°C

This material is not listed as hazardous by ∗NIOSH/RTECS. Therefore, no SAFETY DATA SHEET is required. However, the chemical, physical and toxicological properties of this product have not been thoroughly investigated. Therefore, please exercise due care when handling this material. This action is in compliance with State and Federal OSHA standards and regulations.

Quality Control: ______________

Date: June 20, 2019

CSBio
20 Kelly Court, Menlo Park, CA 94025 USA
T: (650) 322 1111 • F: (650) 322 2278
www.csbio.com • peptides@csbio.com

Compound: GT0361 | N2N-EET-45 Gly-32-Gly | Lot Number: U784 | Expected M.W.: 3297.67 | Found M.W.: 3297.63

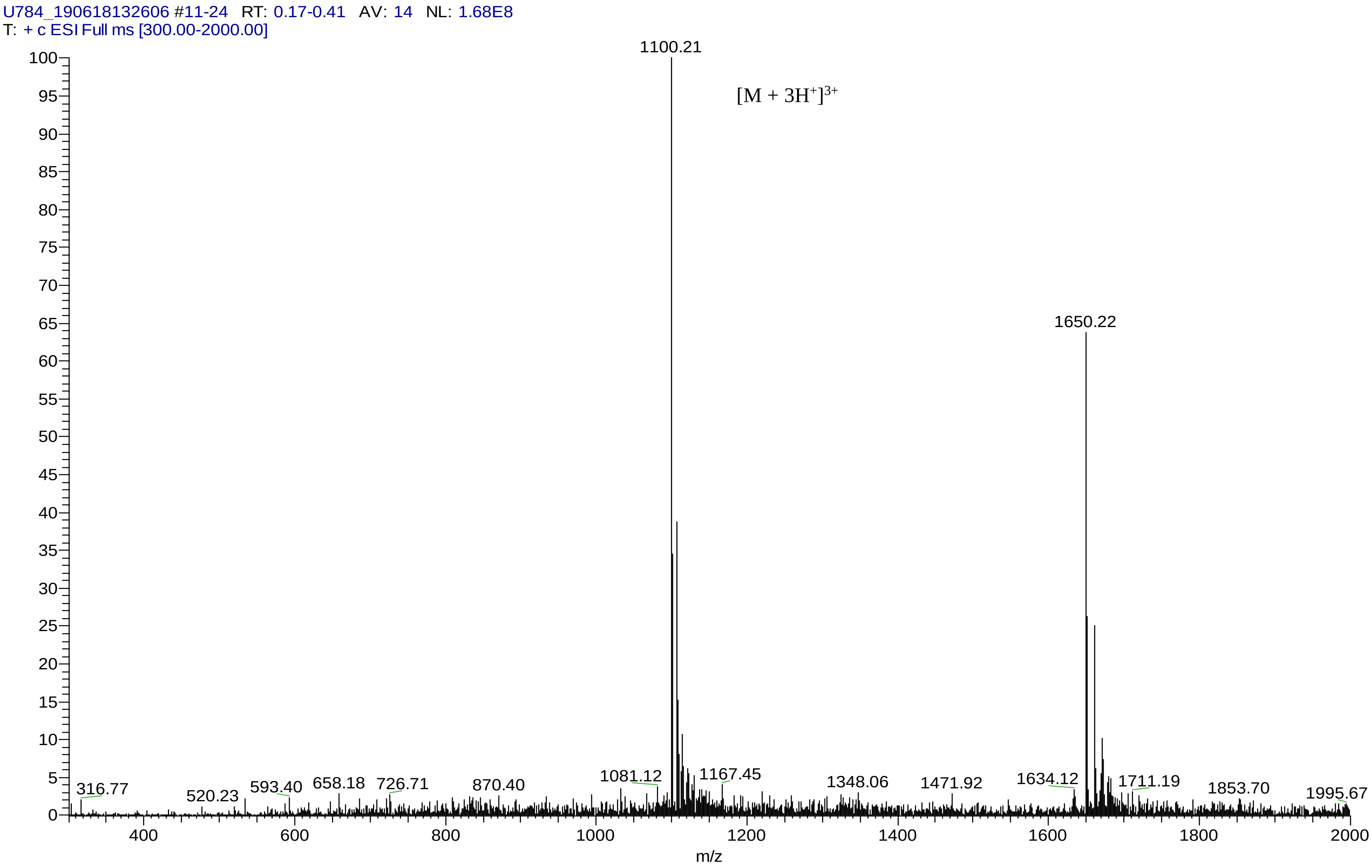

Sample Name: GT0361
Lot#: U784
Instrument 1 Agilent 1260
Instrument ID: RD-HPLC 1
Injection Date: 6/18/2019
Inj. Volume: 50.0 uL

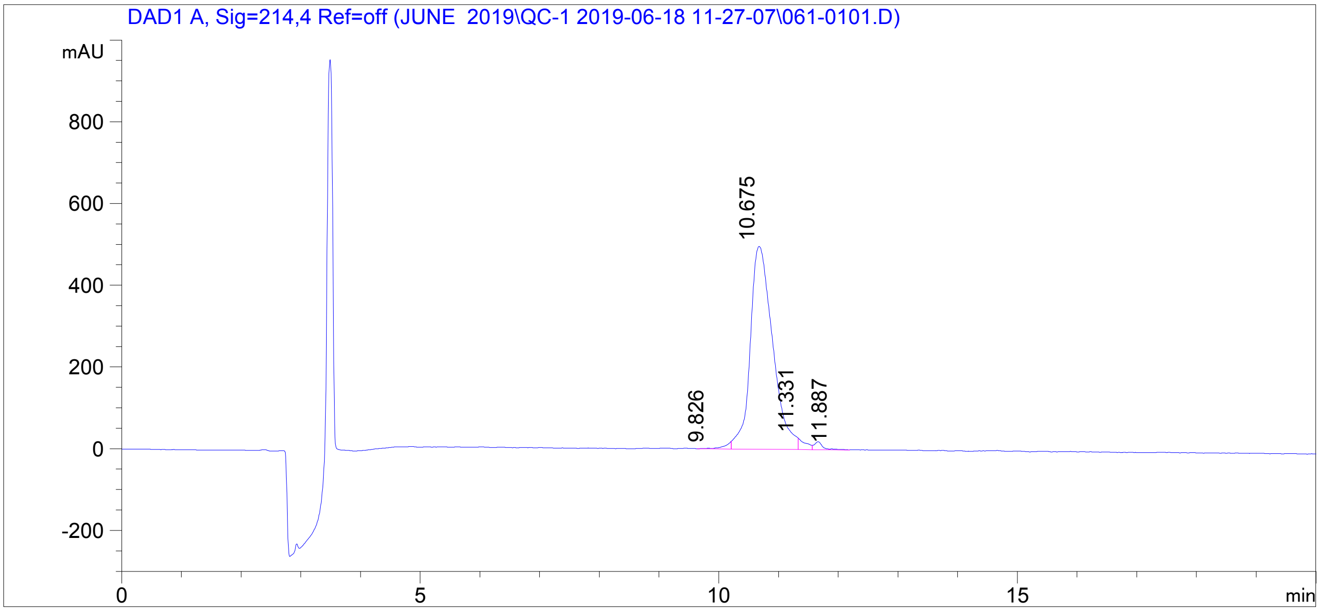

Data file name: C:\CHEM32\1\DATA\JUNE 2019\QC-1 2019-06-18 11-27-07\061-0101.D

Acq. Method: C:\Chem32\1\DATA\JUNE 2019\QC-1 2019-06-18 11-27-07\20-50-20.M

Column: Phenomenex Luna C18, 5um 250 x 4.6mm
Buffer A: 0.1% TFA in H2O
Buffer B: 0.1% TFA in ACN
Flow Rate: 1ml/min
Gradient: 20-50% B in 20 min

Peak #	RT [min]	Area	Height	Area %
1	9.826	13.10	1.33	0.10
2	10.206	129.44	18.29	0.95
3	10.675	12988.13	496.51	95.75
4	11.331	231.79	27.77	1.71
5	11.661	176.63	19.75	1.30
6	11.887	24.98	2.26	0.18
