# Supplementary material for: Disulfide-constrained peptide scaffolds enable a robust peptide-therapeutic discovery platform
Source: PLoS One. 2024 Mar 28;19(3):e0300135. doi: 10.1371/journal.pone.0300135 (PMC10977697; doi:10.1371/journal.pone.0300135)
Supplement: S1 File — A zip file contains 51 pdf files with filenames are the same as the “DCP name” listed in the tables. (ZIP) [file pone.0300135.s004.zip › N2N-EET-57.pdf]

SAMPLE TEST REPORT

Product: N2N-EET-57 Gly-30-Gly
Sequence: Gly-Cys-Arg-Pro-Arg-Trp-Gly-Gly-Trp-His-Cys-Lys-Gln-Asp-Ser-Asp-Cys-Leu-Ala-Gly-Cys-Val-Cys-Gly-Pro-Asn-Gly-Phe-Cys-Gly

Note: Natural Oxidation

Product No.: GT0262 Expected M.W.: 3164.59 Found M.W.: 3165.30 Lot: U003

APPEARANCE: White Powder

MOLECULAR WEIGHT VERIFICATION: Confirmed

PURITY: Instrument: Agilent 1260 System — 98.25%
Condition: HPLC column in TFA System
Gradient: 15-45% Buffer B in 20 minutes
Buffer A: 0.1% TFA in H_2O
Buffer B: 0.1% TFA in ACN
Wavelength: 214 nm
Column: Phenomenex Luna C18 5µm 100Å, 4.6 x 250 mm

ELLMAN'S TEST: Complies

PEPTIDE CONTENT: 76.6%
(By N Elemental Analysis)

SUGGESTIONS FOR PEPTIDE DISSOLUTION: Water

COUNTERIONS PRESENT: TFA Salt

STORAGE: All peptides should be stored dry at -20°C

This material is not listed as hazardous by ∗NIOSH/RTECS. Therefore, no SAFETY DATA SHEET is required. However, the chemical, physical and toxicological properties of this product have not been thoroughly investigated. Therefore, please exercise due care when handling this material. This action is in compliance with State and Federal OSHA standards and regulations.

Quality Control: Xiaosong Jin (signature)

Date: September 12, 2018

CS Bio Co.
20 Kelly Court, Menlo Park, CA 94025 USA
T: (650) 322 1111 • F: (650) 322 2278
www.csbio.com • peptides@csbio.com

Compound: GT0262 N2N-EET-57 Gly-30-Gly Lot Number: U003 Expected M.W.: 3164.59 Found M.W.: 3165.30

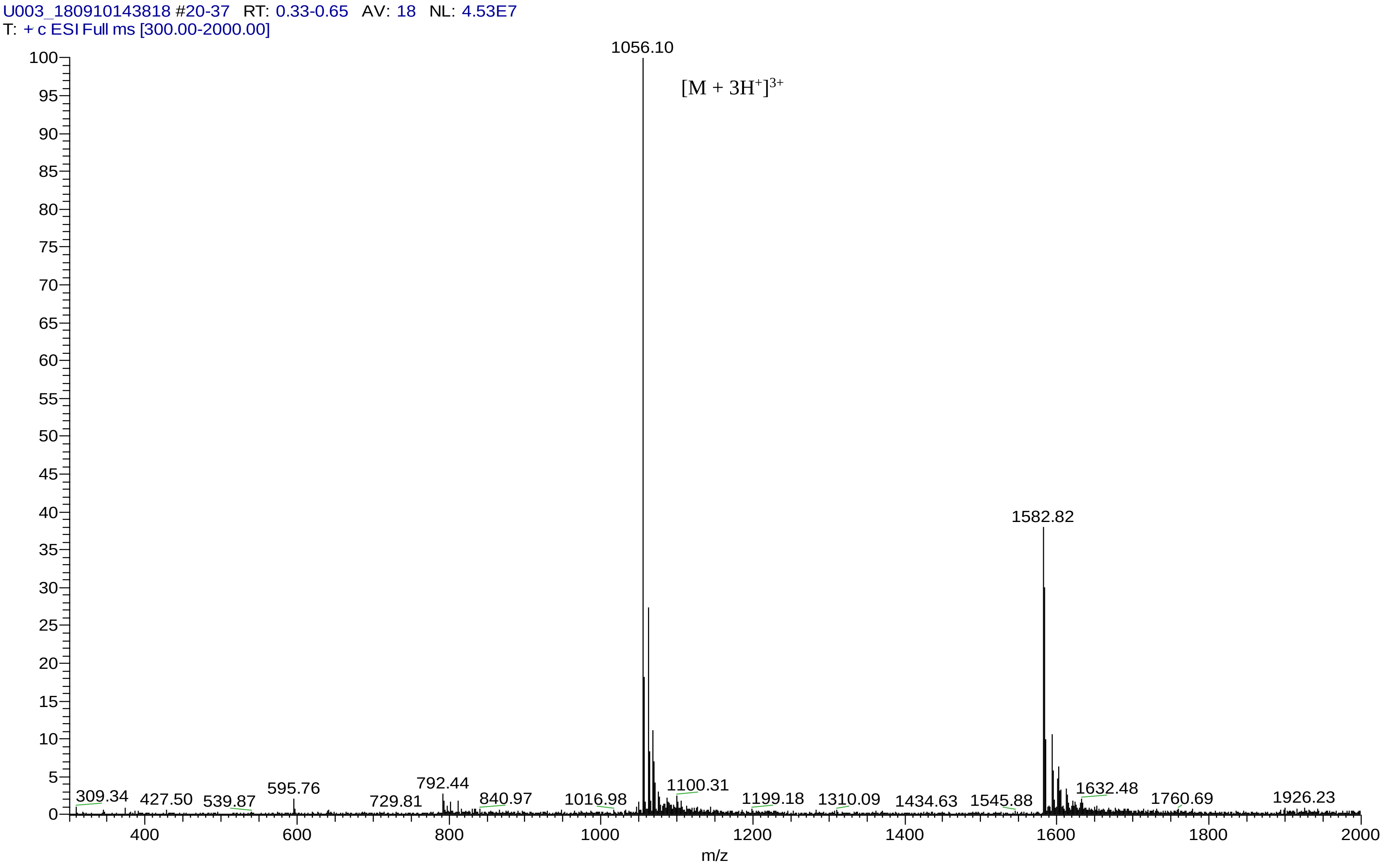

Sample Name: GT0262
Lot#: U003
Instrument 1 Agilent 1260
Instrument ID: RD-HPLC 1
Injection Date: 9/9/2018
Inj. Volume: 50.0 uL

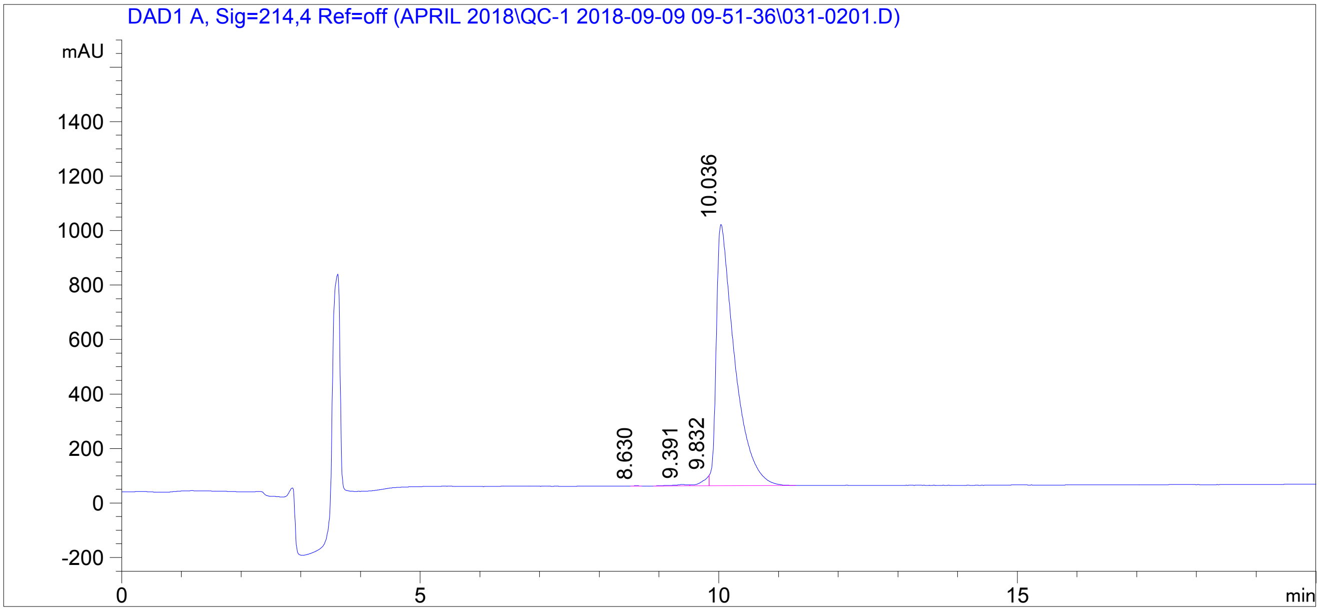

Data file name: C:\CHEM32\1\DATA\APRIL 2018\QC-1 2018-09-09 09-51-36\031-0201.D

Acq. Method: C:\Chem32\1\DATA\April 2018\QC-1 2018-09-09 09-51-36\15-45-20.M

Column: Phenomenex Luna C18 5u 100A 250x4.6mm P/N: 00G-4252-E0
Buffer A: 0.1% TFA in Water
Buffer B: 0.1% TFA in ACN
Flow Rate: 1 ml/min
Gradient: 15 to 45% B in 20 min

Peak #	RT [min]	Area	Height	Area %
1	8.630	6.18	0.93	0.03
2	9.391	90.59	4.93	0.43
3	9.832	268.88	36.86	1.29
4	10.036	20539.03	959.24	98.25

Peptide Content with Elemental Analysis

Analysis: Determination of Peptide Content by Nitrogen Content
Instrument Model: CE-440 Elemental Analyzer
Sample Name: N2N-EET-57 Gly-30-Gly
Sample ID: GT0262
Lot Number: U003
Sample Testing Date: 9/12/2018

	N%
**Expected Content**	19.03
**Actual Content**	14.57
**Peptide Content (%)**	**76.6**

Performed by:

9/12/2018

Name Date

Reviewed by:

9/12/2018

Name Date

CS Bio Co.
20 Kelly Court, Menlo Park, CA 94025 USA
Tel: 650-322-1111 Fax: 650-322-2278 Web: www.csbio.com Email: peptides@csbio.com
