# Supplementary material for: Disulfide-constrained peptide scaffolds enable a robust peptide-therapeutic discovery platform
Source: PLoS One. 2024 Mar 28;19(3):e0300135. doi: 10.1371/journal.pone.0300135 (PMC10977697; doi:10.1371/journal.pone.0300135)
Supplement: S1 File — A zip file contains 51 pdf files with filenames are the same as the “DCP name” listed in the tables. (ZIP) [file pone.0300135.s004.zip › N2N-EET-63.pdf]

SAMPLE TEST REPORT

Product: N2N-EET-63 Gly-30-Gly
Sequence: Gly-Cys-Met-Pro-Arg-Tyr-Gly-Gly-Trp-His-Cys-Lys-Gln-Asp-Ser-Asp-Cys-Leu-Ala-Gly-Cys-Val-Cys-Gly-Pro-Asn-Gly-Phe-Cys-Gly

Note: Natural Oxidation

Product No.: GT0362   Expected M.W.: 3116.50   Found M.W.: 3116.54   Lot: U785

APPEARANCE:	White Powder
MOLECULAR WEIGHT VERIFICATION:	Confirmed
PURITY: Instrument: Agilent 1260 System<br>Condition: HPLC column in TFA System<br>Gradient: 15-45% Buffer B in 20 minutes<br>Buffer A: 0.1% TFA in H_2O<br>Buffer B: 0.1% TFA in ACN<br>Wavelength: 214 nm<br>Column: Phenomenex Luna C18 5µm 100Å, 4.6 x 250 mm	96.56%
PEPTIDE CONTENT:<br>(By N Elemental Analysis)	Pending
ELLMAN'S TEST:	Complies
SUGGESTIONS FOR PEPTIDE DISSOLUTION:	Water
COUNTERIONS PRESENT:	TFA Salt
STORAGE:	All peptides should be stored dry at -20°C

This material is not listed as hazardous by ∗NIOSH/RTECS. Therefore, no SAFETY DATA SHEET is required. However, the chemical, physical and toxicological properties of this product have not been thoroughly investigated. Therefore, please exercise due care when handling this material. This action is in compliance with State and Federal OSHA standards and regulations.

Quality Control: ______________________

Date: June 20, 2019

CSBio
20 Kelly Court, Menlo Park, CA 94025 USA
T: (650) 322 1111 • F: (650) 322 2278
www.csbio.com • peptides@csbio.com

Compound: GT0362 N2N-EET-63 Gly-30-Gly Lot Number: U785 Expected M.W.: 3116.50 Found M.W.: 3116.54

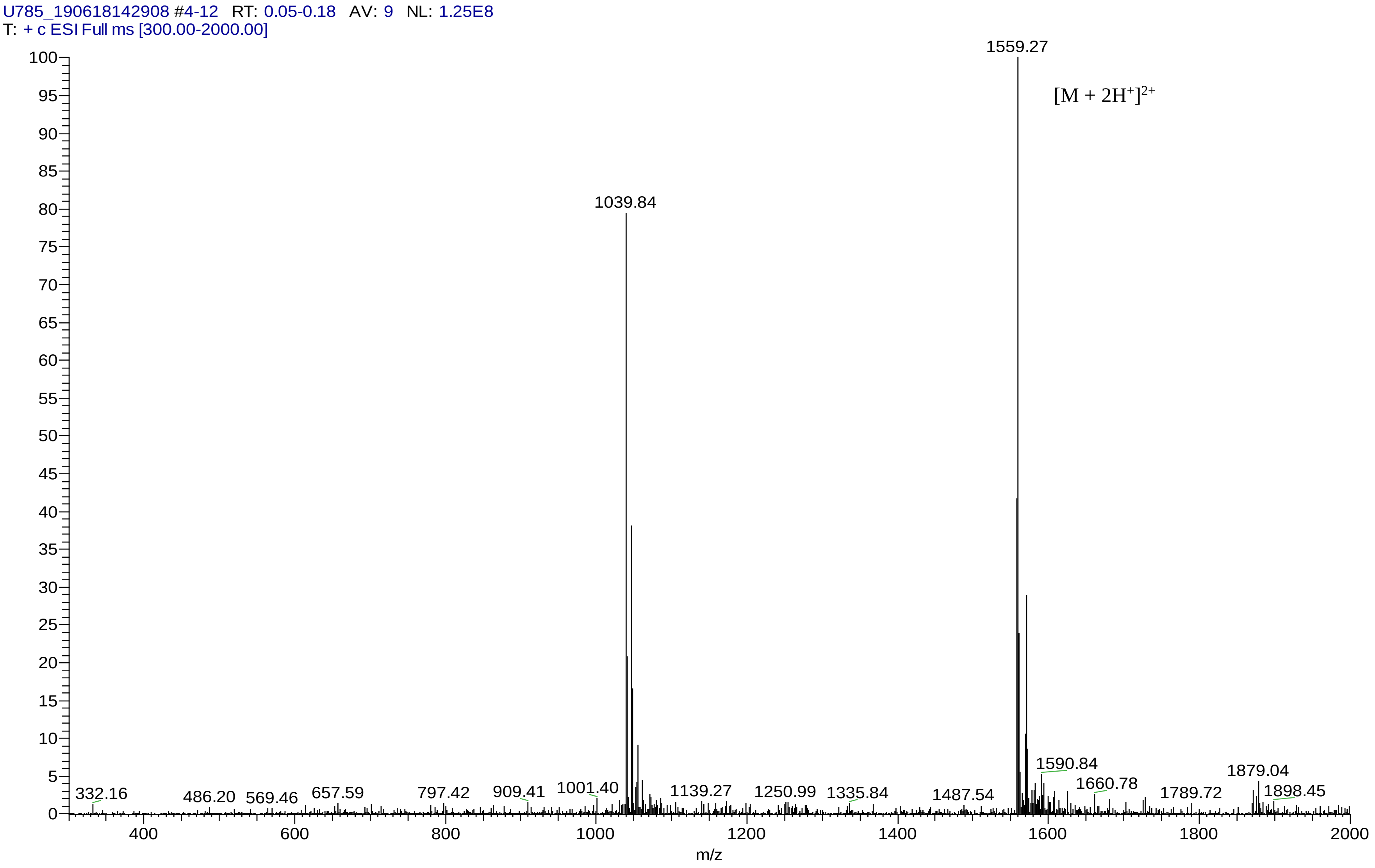

Sample Name: GT0362
Lot#: U785
Instrument 1 Agilent 1260
Instrument ID: RD-HPLC 1
Injection Date: 6/18/2019
Inj. Volume: 20.0 uL

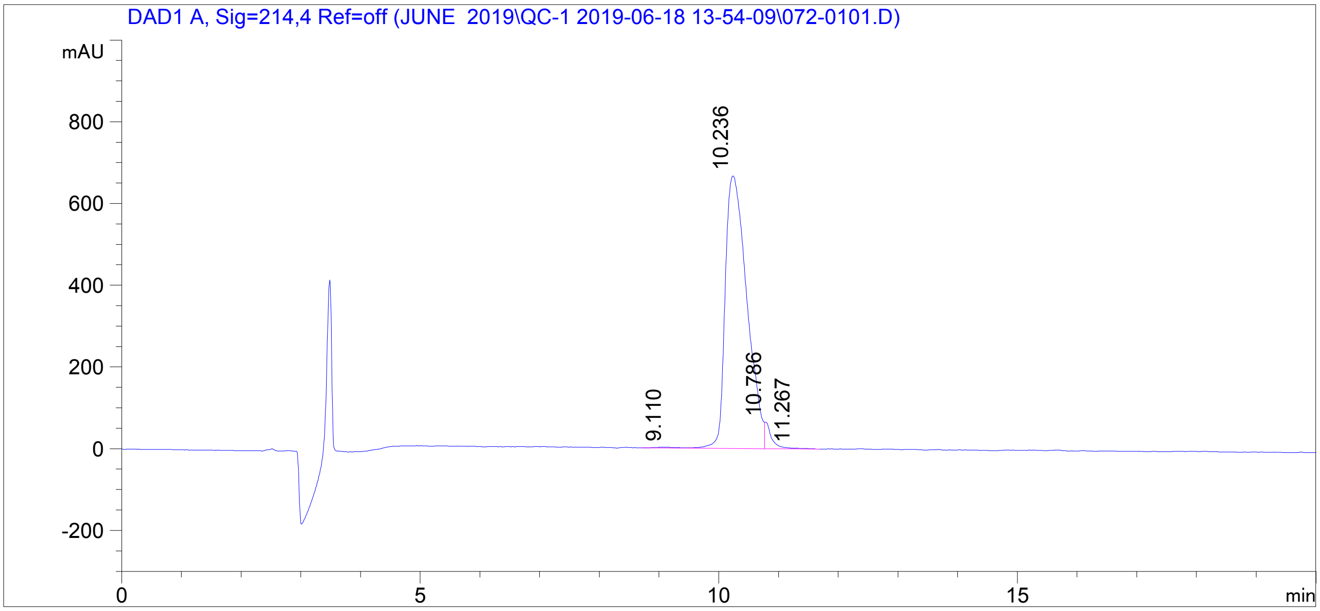

Data file name: C:\CHEM32\1\DATA\JUNE 2019\QC-1 2019-06-18 13-54-09\072-0101.D

Acq. Method: C:\Chem32\1\DATA\JUNE 2019\QC-1 2019-06-18 13-54-09\15-45-20.M

Column: Phenomenex Luna C18 5u 100A 250x4.6mm P/N: 00G-4252-E0
Buffer A: 0.1% TFA in Water
Buffer B: 0.1% TFA in ACN
Flow Rate: 1 ml/min
Gradient: 15 to 45% B in 20 min

Peak #	RT [min]	Area	Height	Area %
1	9.110	70.30	2.35	0.40
2	10.236	16873.62	667.13	96.56
3	10.786	505.93	65.09	2.90
4	11.267	25.59	2.05	0.15
