# Supplementary material for: Disulfide-constrained peptide scaffolds enable a robust peptide-therapeutic discovery platform
Source: PLoS One. 2024 Mar 28;19(3):e0300135. doi: 10.1371/journal.pone.0300135 (PMC10977697; doi:10.1371/journal.pone.0300135)
Supplement: S1 File — A zip file contains 51 pdf files with filenames are the same as the “DCP name” listed in the tables. (ZIP) [file pone.0300135.s004.zip › PCSK9FL_CBD1.pdf]

Quality Control Record

Product: PCSK9FL_CBD1
Sequence: Gly-Pro-Thr-Gln-Ser-Asn-Tyr-Gly-Met-Cys-Gly-Gly-Ile-Gly-Tyr-Ser-Gly-Pro-Thr-Val-Cys-Ala-Ser-Gly-Thr-Thr-Cys-Gln-Val-Leu-Tyr-Pro-Thr-Thr-Ser-Arg-Cys-Leu-Pro-Gly-Ala-Ser

Note: Natural Oxidation

Product No.: GT1311 Expected M.W.: 4140.60 Found M.W.: 4140.00 Lot: X099

APPEARANCE: White Powder

MOLECULAR WEIGHT VERIFICATION: Confirmed

PURITY: Instrument: Agilent 1290 — 97.65%
Condition: HPLC column in TFA System
Gradient: 20-50% Buffer B in 20 minutes
Buffer A: 0.1% TFA in H2O
Buffer B: 0.1% TFA in ACN
Wavelength: 214 nm
Column: Phenomenex Luna C18 5µm 100Å, 4.6 x 250 mm

PEPTIDE CONTENT: (By N Elemental Analysis) — 88.6%

ELLMAN'S TEST: Complies

SUGGESTIONS FOR PEPTIDE DISSOLUTION: 0.1% TFA in Water

COUNTERIONS PRESENT: TFA Salt

STORAGE: All peptides should be stored dry at -20°C

This material is NOT FOR HUMAN USE. This material is not listed as hazardous by ∗NIOSH/RTECS. Therefore, no SAFETY DATA SHEET is required. However, the chemical, physical and toxicological properties of this product have not been thoroughly investigated. Therefore, please exercise due care when handling this material. This action is in compliance with State and Federal OSHA standards and regulations.

Quality Control: ____________

Date: February 4, 2022

CSBio
20 Kelly Court, Menlo Park, CA 94025 USA
T: (650) 322 1111 • F: (650) 322 2278
www.csbio.com • peptides@csbio.com

Compound: GT1311 PCSK9FL_CBD1 Lot Number: X099 Expected M.W.: 4140.60 Found M.W.: 4140.00

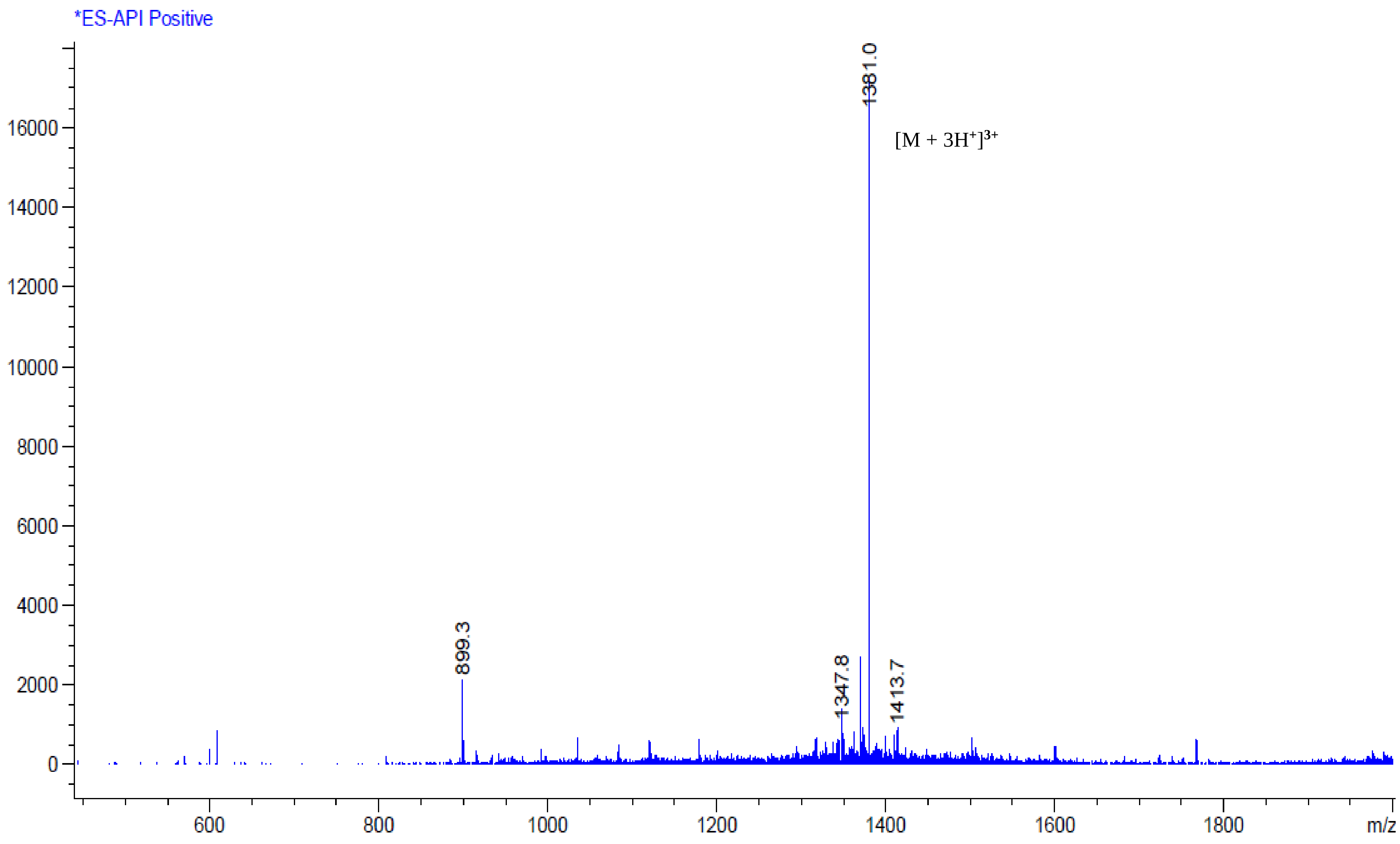

Sample Name: GT1311
Lot# X099
Instrument 1 Agilent 1290
Instrument ID: E195
Injection Date: 1/28/2022
Inj. Volume: 30.0 uL

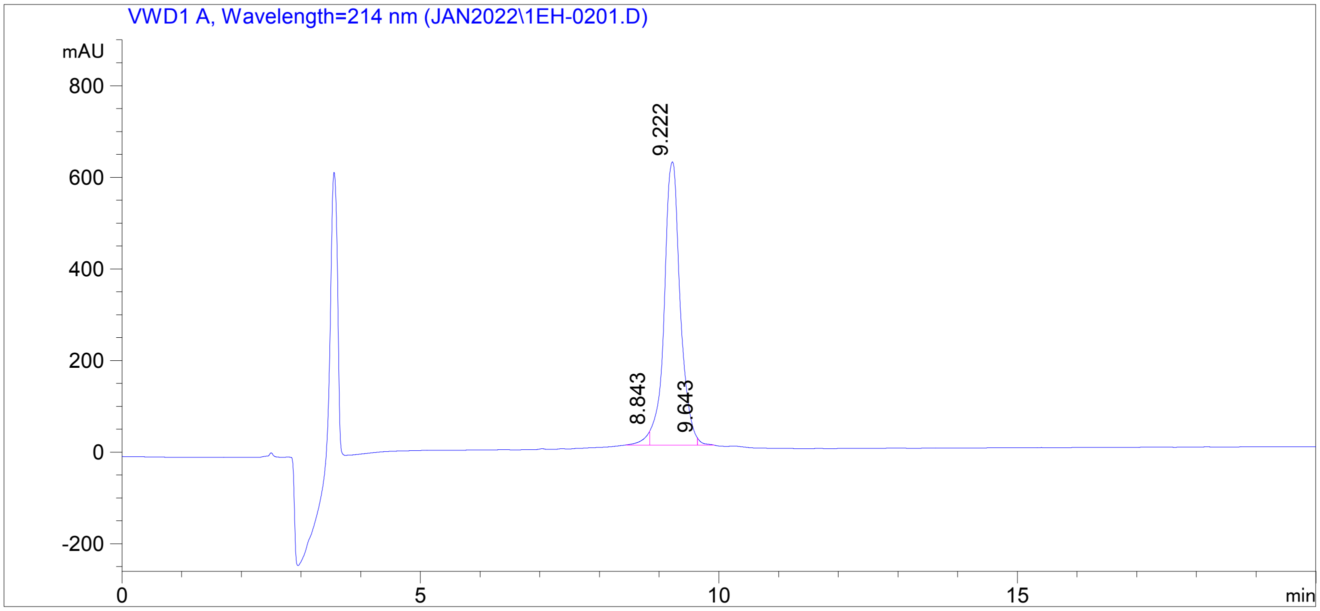

Data file name: C:\CHEM32\1\DATA\JAN2022\1EH-0201.D
Acq. Method: C:\Chem32\1\DATA\JAN2022\QC 2022-01-28 09-18-14\20-50-20-1-2.M

Column: Phenomenex Luna C18, 5um 250 x 4.6mm P/N: 00G-4252-E0
Buffer A: 0.1% TFA in H2O
Buffer B: 0.1% TFA in ACN
Wavelength: 214 nm
Flow Rate: 1ml/minute
Column Temperature: 25c
Gradient: 20%-50% B in 20 minutes

Peak #	RT [min]	Area	Height	Area %
1	8.843	215.71	30.91	1.83
2	9.222	11507.42	618.40	97.65
3	9.643	61.77	14.68	0.52

Peptide Content with Elemental Analysis

Analysis: Determination of Peptide Content by Nitrogen Content
Instrument Model: Perkin Elmer Series II CHNS/O Analyser 2400
Sample Name: PCSK9FL_CBD1
Sample ID: GT1311
Lot Number: X099
Sample Testing Date: 2/4/2022

	N%
**Expected Content**	16.24
**Actual Content**	14.38
**Peptide Content (%)**	**88.6**

Performed by:

2/4/2022

Name Date

Reviewed by:

2/4/2022

Name Date

CSBio
20 Kelly Court, Menlo Park, CA 94025 USA
Tel: 650-322-1111 Fax: 650-322-2278 Web: www.csbio.com Email: peptides@csbio.com
