# Supplementary material for: Disulfide-constrained peptide scaffolds enable a robust peptide-therapeutic discovery platform
Source: PLoS One. 2024 Mar 28;19(3):e0300135. doi: 10.1371/journal.pone.0300135 (PMC10977697; doi:10.1371/journal.pone.0300135)
Supplement: S1 File — A zip file contains 51 pdf files with filenames are the same as the “DCP name” listed in the tables. (ZIP) [file pone.0300135.s004.zip › PCSK9FL_CnTx1.pdf]

Quality Control Record

Product: PCSK9FL_CrTx1
Sequence: Glu-Cys-Lys-Gly-Lys-Gly-Ala-Lys-Cys-Ser-Tyr-Tyr-Met-Gly-Ile-Asp-Lys-Gly-Tyr-Val-Asn-Tyr-Cys-Cys-Thr-Gly-Ser-Cys-Arg-Ser-Gly-Lys-Cys

Note: Natural Oxidation

Product No.: GT1308 Expected M.W.: 3558.14 Found M.W.: 3557.97 Lot: X146

APPEARANCE: White Powder

MOLECULAR WEIGHT VERIFICATION: Confirmed

PURITY: Instrument: Agilent 1290 — 93.60%
Condition: HPLC column in TFA System
Gradient: 15-45% Buffer B in 20 minutes
Buffer A: 0.1% TFA in H2O
Buffer B: 0.1% TFA in ACN
Wavelength: 214 nm
Column: Phenomenex Luna C18 5µm 100Å, 4.6 x 250 mm

PEPTIDE CONTENT: (By N Elemental Analysis) Pending

ELLMAN'S TEST: Complies

SUGGESTIONS FOR PEPTIDE DISSOLUTION: 0.1% TFA in Water

COUNTERIONS PRESENT: TFA Salt

STORAGE: All peptides should be stored dry at -20°C

This material is NOT FOR HUMAN USE. This material is not listed as hazardous by ∗NIOSH/RTECS. Therefore, no SAFETY DATA SHEET is required. However, the chemical, physical and toxicological properties of this product have not been thoroughly investigated. Therefore, please exercise due care when handling this material. This action is in compliance with State and Federal OSHA standards and regulations.

Quality Control: ____________________

Date: February 8, 2022

CSBio
20 Kelly Court, Menlo Park, CA 94025 USA
T: (650) 322 1111 • F: (650) 322 2278
www.csbio.com • peptides@csbio.com

Compound: GT1308 PCSK9FL_CrTx1 Lot Number: W146 Expected M.W.: 3558.14 Found M.W.: 3557.97

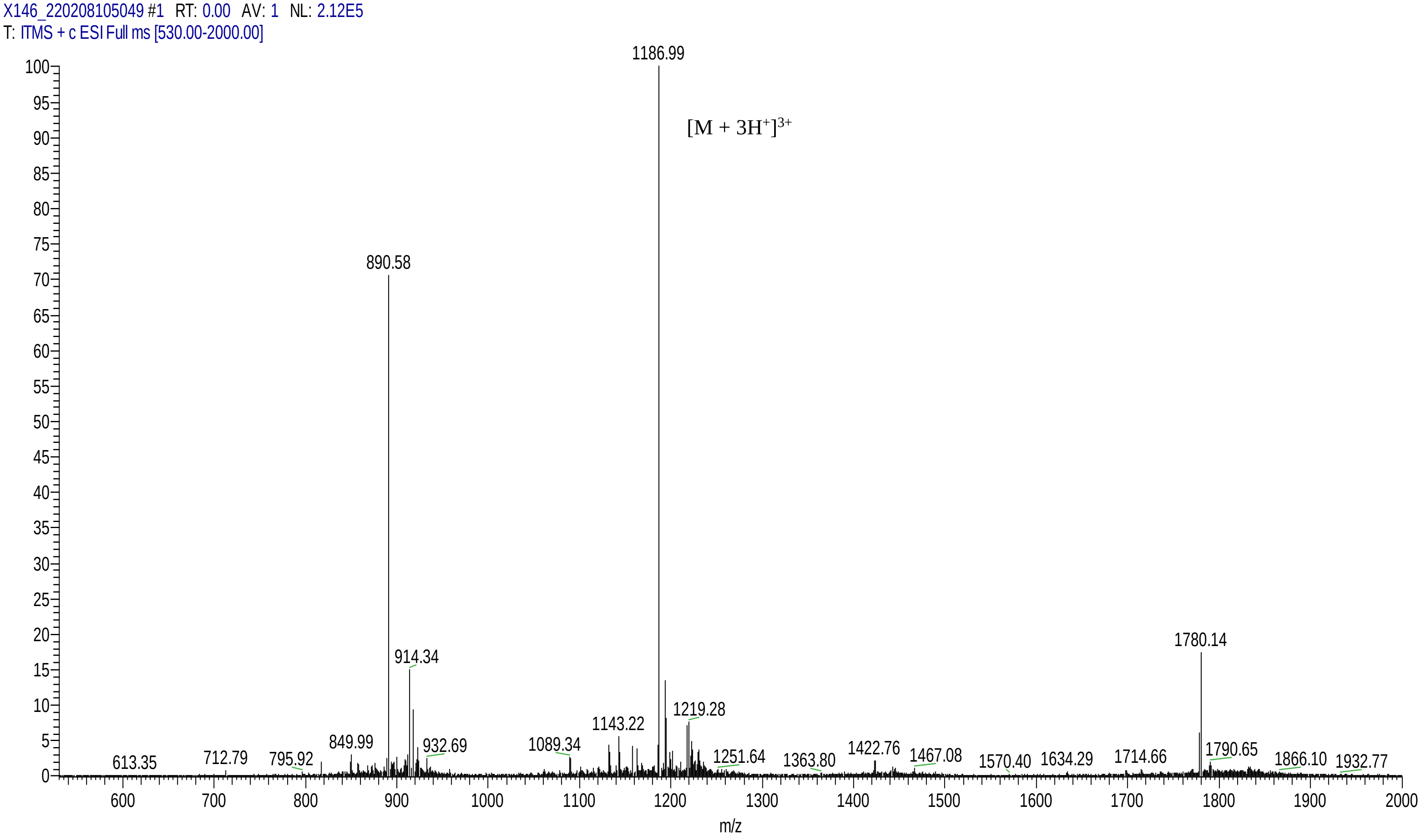

Sample Name: GT1308
Lot# X146
Instrument 1 Agilent 1290
Instrument ID: E195
Injection Date: 2/7/2022
Inj. Volume: 10.0 uL

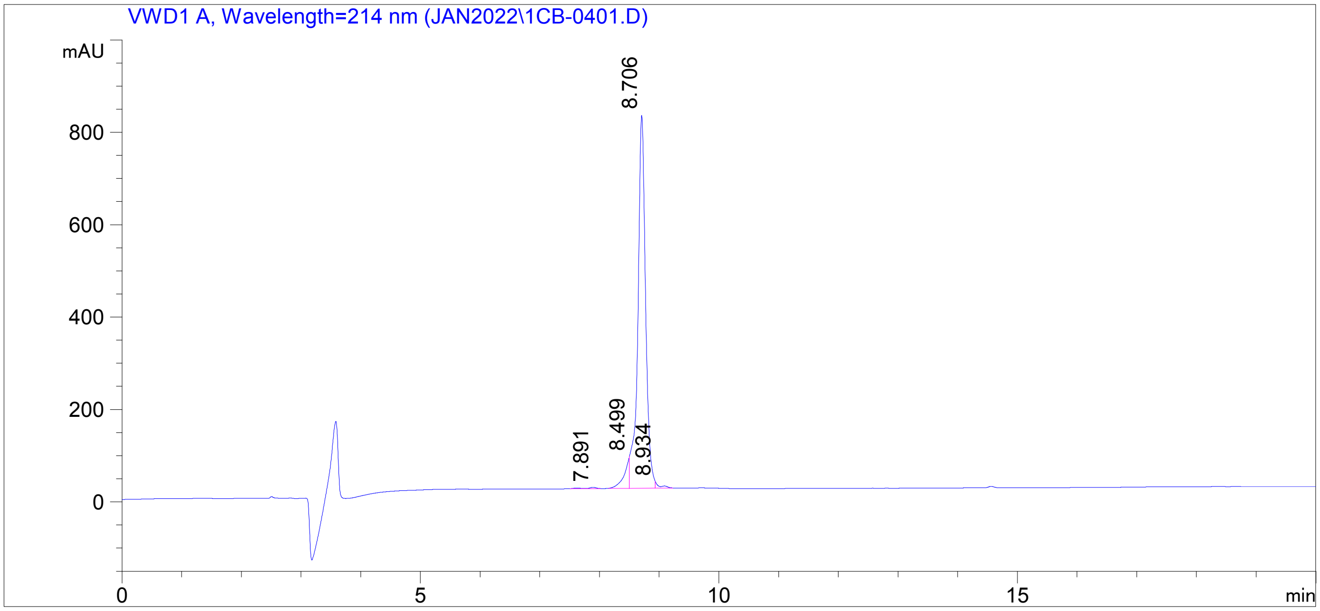

Data file name: C:\CHEM32\1\DATA\JAN2022\1CB-0401.D
Acq. Method: C:\Chem32\1\DATA\FEB2022\QC 2022-02-07 18-10-49\15-45-20.-1-2.M

Column: Phenomenex Luna C18 5u 100A 250x4.6mm P/N: 00G-4252-E0
Buffer A: 0.1% TFA in Water
Buffer B: 0.1% TFA in ACN
Wavelength: 214 nm
Flow Rate: 1 ml/minute
Column Temperature: 25c
Gradient: 15% to 45% B in 20 minutes

Peak #	RT [min]	Area	Height	Area %
1	7.891	33.51	2.79	0.43
2	8.499	396.56	68.31	5.10
3	8.706	7273.18	807.70	93.60
4	8.934	66.90	14.15	0.86
