# Supplementary material for: Disulfide-constrained peptide scaffolds enable a robust peptide-therapeutic discovery platform
Source: PLoS One. 2024 Mar 28;19(3):e0300135. doi: 10.1371/journal.pone.0300135 (PMC10977697; doi:10.1371/journal.pone.0300135)
Supplement: Attachment — Submitted filename: RespToRevLetter022124.docx [file pone.0300135.s008.docx]

Dear Editor:

Thank you for your careful review of our revised manuscript titled “Disulfide-constrained peptide scaffolds enable a robust peptide-therapeutic discovery platform” (PONE-D-23-32053R1). We appreciated the positive feedbacks and comments from both reviewers. Following are our response to one point raised by Reviewer1. The reviewer’s comment is in bold font and our response are in regular font:

**Reviewer #1: On the whole I am happy that the authors have addressed mine and the other reviewers comments. However I disagree with one response:

Original reviewing comment: - similar library approaches have been applied with mRNA display e.g. Liu et al
JACS 143 18481 2021 and this work should be mentioned and cited in the
introduction.
Response: Add “peptide library approaches have been applied with mRNA display” at line 44 and
the reference is included.

Further comment: This sentence has been added before the comments on DCPs as if it refers only to general mRNA display screens. My point was rather that mRNA display has been used to create modified DCP peptides in a similar manner (using a cyclotide MCoTI-II scaffold) to the screens in this paper (as shown in the cited paper) and this is what I think should be made clear in the introduction.**

We have modified the Introduction to clarify that mRNA display had been successfully applied to DCP library strategies and our phage display approach has extended the applicability of the strategy. From line 43-49, the new text is:

“To overcome some of these peptide-intrinsic shortcomings, we considered using naturally occurring and highly stable disulfide constrained peptides (DCPs) as frameworks coupled with display technology aimed at generating de novo binders of therapeutically relevant target proteins [3]. Notebly, DCP library strategies has been effectively employed using mRNA display with MCoTI-II as a scaffold [4]. In this study, we employed phage display across seven distinct scaffolds to extent the applicability of this approach. “

Yours truly,

Yingnan Zhang, Ph.D. and Rami N. Hannoush, Ph.D.
